# Further New Diterpenoids as PTP1B Inhibitors from the Xisha Soft Coral *Sinularia polydactyla*

**DOI:** 10.3390/md16040103

**Published:** 2018-03-25

**Authors:** Fei Ye, Zheng-Dan Zhu, Yu-Cheng Gu, Jia Li, Wei-Liang Zhu, Yue-Wei Guo

**Affiliations:** 1 State Key Laboratory of Drug Research, Shanghai Institute of MateriaMedica, Chinese Academy of Sciences, 555 Zu Chong Zhi Road, Zhangjiang Hi-Tech Park, Shanghai 201203, China; simmyefei@simm.ac.cn (F.Y.); zdzhu@simm.ac.cn (Z.-D.Z.); jli@simm.ac.cn (J.L.); wlzhu@simm.ac.cn (W.-L.Z.); 2 University of Chinese Academy of Sciences, No. 19A Yuquan Road, Beijing 100049, China; 3 Syngenta, Jealott’s Hill International Research Centre, Bracknell, Berkshire RG42 6EY, UK; yucheng.gu@syngenta.com; 4 Open Studio for Druggability Research of Marine Lead Compounds, Qingdao National Laboratory for Marine Science and Technology, 1 Wenhai Road, Aoshanwei, Jimo, Qingdao 266237, China

**Keywords:** *Sinularia polydactyla*, diterpenoids, PTP1B inhibitory activity

## Abstract

A new prenyleudesmane type diterpene, sinupol (**8**), and a new capnosane type diterpenoid, sinulacetate (**9**), were isolated from the Xisha soft coral *Sinularia polydactyla* along with five known related diterpenes (**4**–**7** and **10**). Their structures, including absolute configurations, were determined by extensive spectroscopic analysis, the comparison of their NMR data with those of related compounds, and time-dependent density functional theory electronic circular dichroism (TDDFT ECD) calculations. Both new compounds (**8** and **9**) exhibited promising inhibitory activity against protein tyrosine phosphatase 1B (PTP1B), a potential drug target for the treatment of type II diabetes and obesity.

## 1. Introduction

Soft corals of the genus *Sinularia*, belonging to the phylum Cnidaria, class Alcyonaria, and family Alcyoniidae, are a prolific source of structurally diverse (such as diterpenoids, polyhydroxylated steroids, alkaloids, polyamines, etc.) and biologically active metabolites. These organisms have been the subjects of intensive research by natural product chemists and pharmacologists for their chemical diversities and broad pharmacological activity, such as cytotoxic, antiviral, and anti-inflammatory properties [1,2]. To date, more than 50 species of *Sinularia* have been chemically investigated, with over 500 diverse and bioactive metabolites being elucidated [3]. 

**Figure 1:**
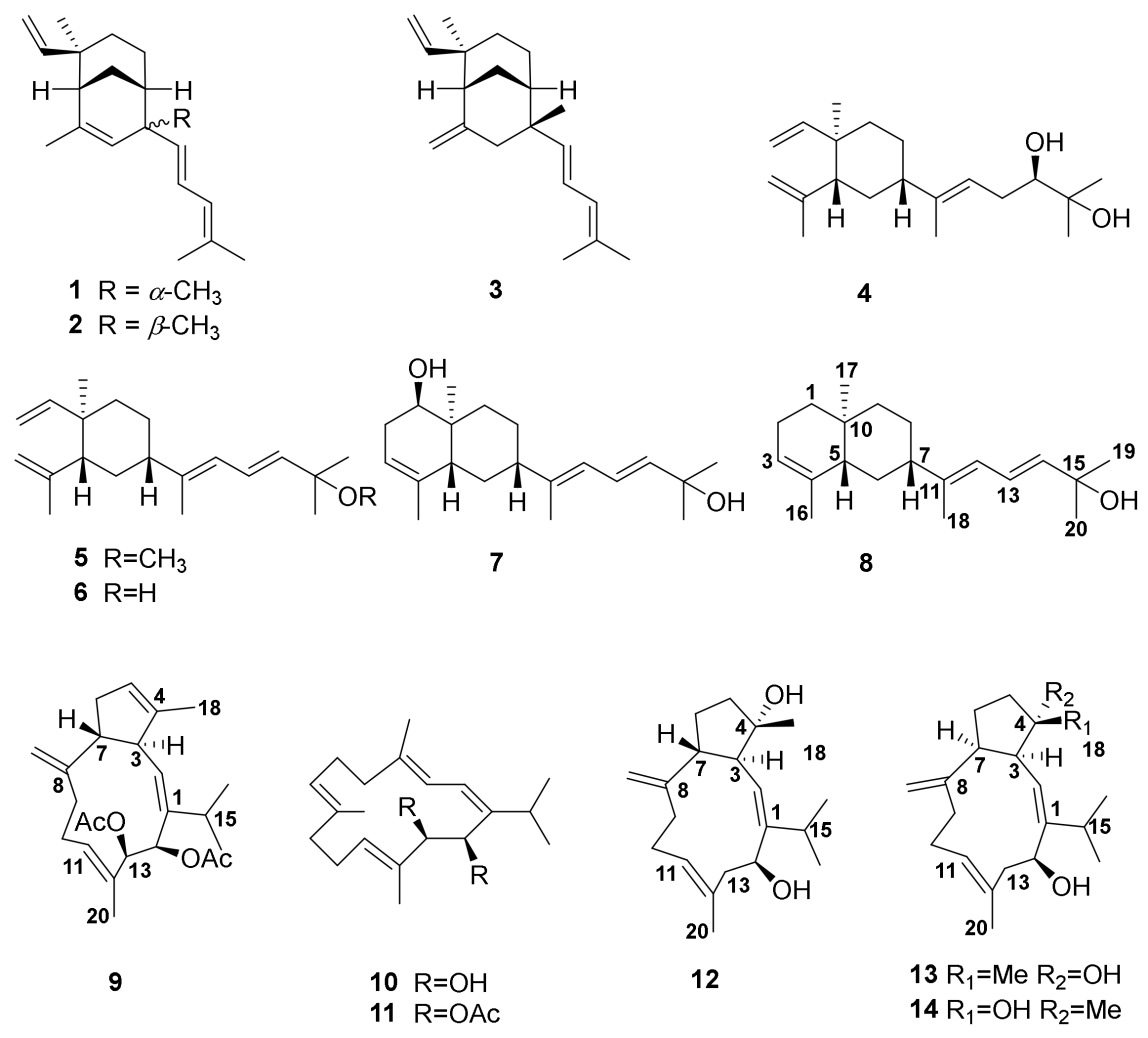
Structures of compounds **1**–**14**.

In the course of our ongoing project searching for novel and bioactive compounds from Chinese soft corals [3,4,5,6,7,8,9], the title animal *Sinularia polydactyla* was collected from the Xisha Islands in the South China Sea, Hainan Province, China. Among all of the *Sinularia* species, *S. polydactyla* in the Red Sea has been extensively investigated, while there is only one report regarding the chemical constituents of *S. polydactyla* from the South China Sea, with only two sesquiterpenoids being characterized [3]. Our previous chemical investigation of *S. polydactyla* revealed the rich chemical diversity of this biological material and led to the discovery of three uncommon novel diterpenes with unprecedented carbon skeletons (**1**–**3**) [3] displaying an interesting dose-dependent promotion effect on ConA-induced T lymphocyte proliferation. Stimulated by this discovery, and in order to find more chemically interesting and biologically active metabolites, especially trace components, we recently carried out a further chemical investigation of the Et_2_O-soluble fraction of the title animal. This investigation resulted in the isolation and characterization of a new prenyleudesmane diterpene, sinupol (**8**), and a new capnosane diterpene, sinulacetate (**9**), together with five known related diterpenoids (**4**–**7** and **10**) (Figure 1). The structure, including the stereochemistry of sinulacetate (**9**), was determined by a comparison of the NMR data with those of the model compounds **11**–**14**. Described herein are the isolation, structure elucidation, and PTP1B inhibitory activity of these newly isolated compounds.

## 2. Results

On the basis of a detailed analysis of all of the isolated molecules and by comparison of their ^1^H NMR and ^13^C NMR spectral data and [α]_D_ values with those reported in the literature, **4**–**7** and **10** were readily identified as a lobane-type diterpenoid, loba-8,10,13(15)-triene-17,18-diol (**4**) [10], lobatetraene (**5**) [10], fuscol (**6**) [9,10], **7**, an unnamed prenyleudesmane diterpene [11], and sarcophytol B (**10**) [12], respectively.

**Figure 2:**
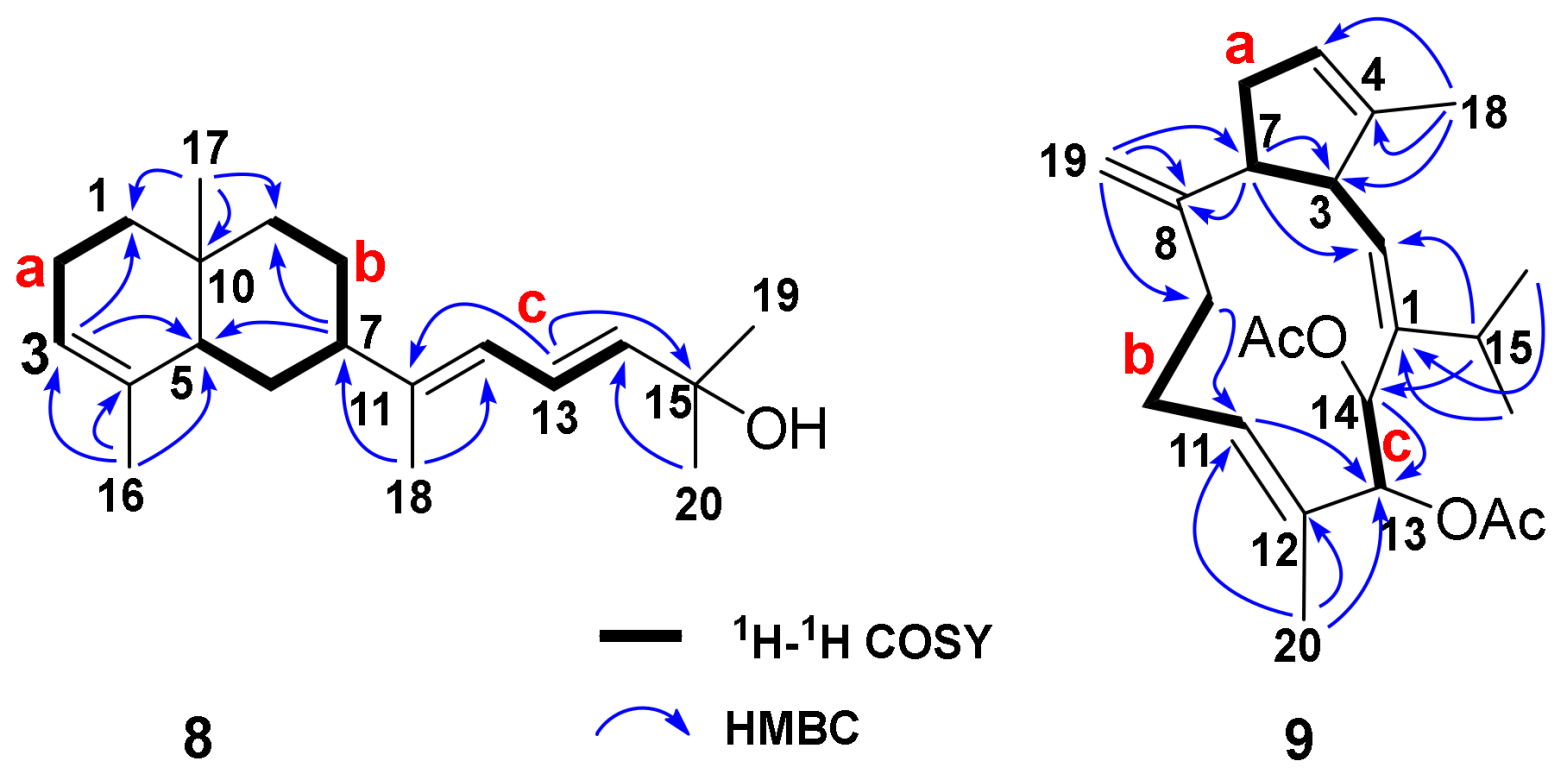
Key HMBC and ^1^H–^1^H COSY correlations of compounds **8** and **9**.

Sinupol (**8**) was obtained as a colorless oil. Its molecular formula was established as C_20_H_32_O by (+)-HR-EIMS, indicating five degrees of unsaturation in the molecule. The ^13^C NMR and HSQC spectra of **8** exhibited the presence of 20 carbon resonances, including five methyl groups, five sp^3^ methylene, two sp^3^ methine, two sp^3^ quaternary, four sp^2^ methine, and two sp^2^ quaternary carbons. A detailed comparison of the NMR data of **8** with those of **7** suggested that **8** is a 1-dehydroxyl product of **7** for the identical chemical shifts in the side chains and significant carbon signal differences (*δ*_C-1_ 76.9, *δ*_C-2_ 33.0, and *δ*_C-10_ 38.5 for **7**, *δ*_C-1_ 38.1, *δ*_C-2_ 23.2, and *δ*_C-10_ 32.4 for **8**) around C-1. To fully elucidate the structure of **8**, its two-dimensional (2D) NMR spectra were extensively analyzed. The ^1^H and ^13^C NMR resonances revealed the presences of one disubstituted and two trisubstituted double bonds, including a conjugated diene fragment (Figure 2). Since the three double bonds accounted for three degrees of unsaturation, the remaining two degrees of unsaturation were attributed to a bicyclic ring system in **8**. Further analysis of the ^1^H–^1^H COSY spectrum revealed the presence of three coupling systems (**a**–**c**) from H_2_-1 to H-3, H-5 to H_2_-9, and H-12 to H-14 (Figure 2).The key HMBC correlations from H_3_-16 to C-3/C-4/C-5, from H_3_-17 to C-1/C-5/C-10/C-9, and from H_2_-6 to C-4/C-5/C-10/C-11 confirmed the presence of the bicyclic ring moiety. The side chain was connected to C-7 of the decalin ring moiety through C-11, and was supported by crosspeaks from H_3_-18 to C-7/C-11/C-12 and from H_3_-19/H_3_-20 to C-14/C-15 in the HMBC spectrum. Thus, the planar structure of **8** was confirmed (Figure 2).

**Figure 3:**
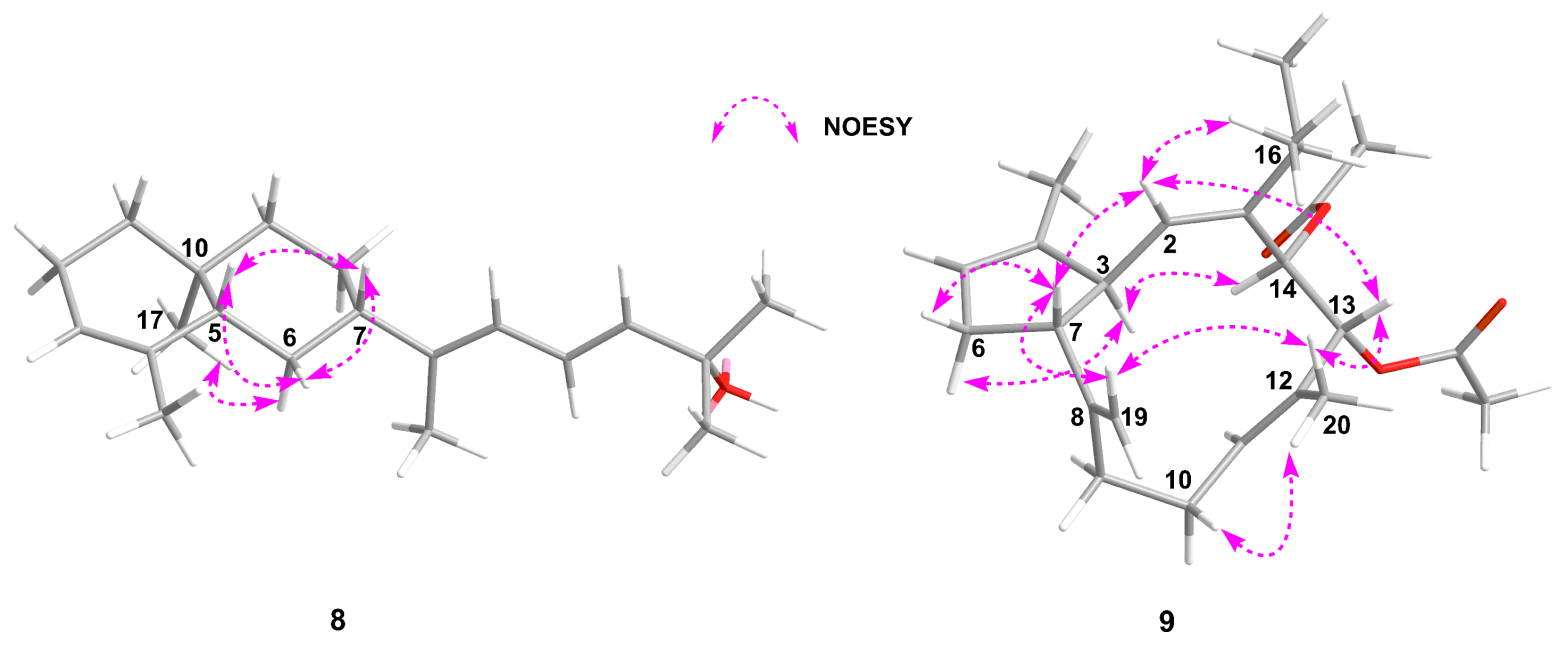
Key NOE correlations of compounds **8** and **9**.

As for the relative configuration of **8**, the *E* geometry of Δ^11,12^ and Δ^13,14^ was established by the NOE correlation from H-13 to CH_3_-18 and the coupling constants between H-13 (*δ*_H_ 6.96, dd, *J* = 11.2, 15.3 Hz) and H-14 (*δ*_H_ 6.13, d, *J* = 15.3 Hz) (Table 1). On the basis of the large coupling constant of H-5 (*δ*_H_ 1.90, dd, *J* = 12.7, 1.1 Hz), the proton of H-5 was determined as an axial orientation. The correlations from H-6β to H-5 and H-7 in the NOESY spectrum defined the *cis*-configuration of H-5 and H-7 (Figure 3). Additionally, the H_3_-17 correlated with H-6α, which confirmed the *trans*-configuration between CH_3_-17 and H-5 (Figure 3). The relative configuration of the C-5, C-7, and C-10 in **8** was thus confirmed as S*, S*, S*, respectively.

Sinulacetate (**9**) was obtained as a colorless oil. An (+)-HR-EIMS data analysis of **9** indicated the molecular formula C_24_H_34_O_4_, which suggested the presence of eight degrees of unsaturation. By inspection of the ^13^C and ^1^H NMR spectra, the presence of two acetoxyl groups was immediately recognized (*δ*_C_ 169.8 and 170.5, respectively). Eight carbon signals in the downfield implied the occurrence of four double bonds in the molecule, including one terminal sp^2^ methylene (*δ*_C_ 113.3). The remaining two degrees of unsaturation were due to the presence of two fused rings in **9**. Detailed analysis of the ^1^H–^1^H COSY spectrum of **9** readily characterized three coupling systems (**a**–**c**) (Figure 2) by the strong correlations of H-2/H-3/H-7/H_2_-6/H-5, H_2_-9/H_2_-10/H-11, and H-13/H-14, respectively. The key HMBC correlations from H_3_-18 to C-3/C-5, H-7 to C-2/C-3/C-8/C-19, H_2_-19 to C-8/C-9, H_2_-10 to C-12, H-11 to C-20/C-13, H_3_-20 to C-11/C-12/C-13, H-14 to C-1/C-13/C-12, H-15 to C-1/C-2/C-14, and H_3_-16/H_3_-17 to C-15/C-1 confirmed the connections of these coupling systems. In view of the above evidences, **9** belongs to a capnosane-type of diterpenoids. Further analysis of the HMBC crosspeaks from H-13 to the carbonyl group at *δ*_C_ 169.8 and H-14 to the carbonyl at *δ*_C_ 170.5 indicated that the acetoxyl acetate groups were on C-14 and C-13, respectively.

The relative stereochemistry of **9** was deduced by analysis of the NMR data and correlations from ROESY spectra. Correlations from H_2_-10 to CH_3_-20 and from H-2 to CH_3_-16 in the ROESY spectrum confirmed the *E* geometry of Δ^11,12^ and the *Z* geometry of Δ^1,2^ in **9**. A typical triplet with *J*_H–H_ values of 9.5 Hz of H-3 indicated that the dihedral angles between H-3 and H-7 and between H-3 and H-2 were approximately 0° or 180°. The ROESY correlations from H-6β to H-7 and from H-6α to H-3 implied the *trans*-configuration of H-3 and H-7. The *thans*-configuration of H-3 and H-7 in **9** was further confirmed by the similar multiplicity and coupling constants of H-3 and H-7 with those of sarcophytol L (**12**) [12], as well as the quite different coupling constants from those of trocheliophols H (**13**) and I (**14**) [13] possessing the *cis*-configuration of H-3 and H-7 (Table 2). The absence of a crosspeak between H-3 and H-7 in the ROESY spectrum in **9** was also consistent with the above conclusion. As for the relative configurations at C-13 and C-14, the similar carbon chemical shift values between **9** (C-13 (*δ*_C_ 77.1) and C-14 (*δ*_C_ 73.6)) (Table 1) and **11** (C-13 (*δ*_C_ 77.8) and C-14 (*δ*_C_ 74.2)) together with the similar coupling constants for protons of **9** (H-13, 4.95, d, *J* = 10.2 Hz; H-14, 6.02, d, *J* = 10.2 Hz) and **11** (H-13, 5.34, d, *J* = 10.0 Hz; H-14, 6.15, d, *J* = 10.0 Hz) [14] indicated the *threo* relative stereochemistry at C-13 and C-14 in **9**. Key crosspeaks from H_2_-19 to H-7/H_3_-20 and from H-13 to H-2/H_3_-20 in the ROESY spectrum indicated the same orientation (*β*) of the protons at H-7, H_2_-19, H_3_-20, H-13, and H-2 in the three-dimensional (3D) model (Figure 3). The *α*-orientation of H-14 was determined by the strong ROESY correlation from H-3 to H-14 (Figure 3). Thus, the relative configuration of the carbons in **9** was determined as 3S*, 7R*, 13R*, 14R*.

**Figure 4:**
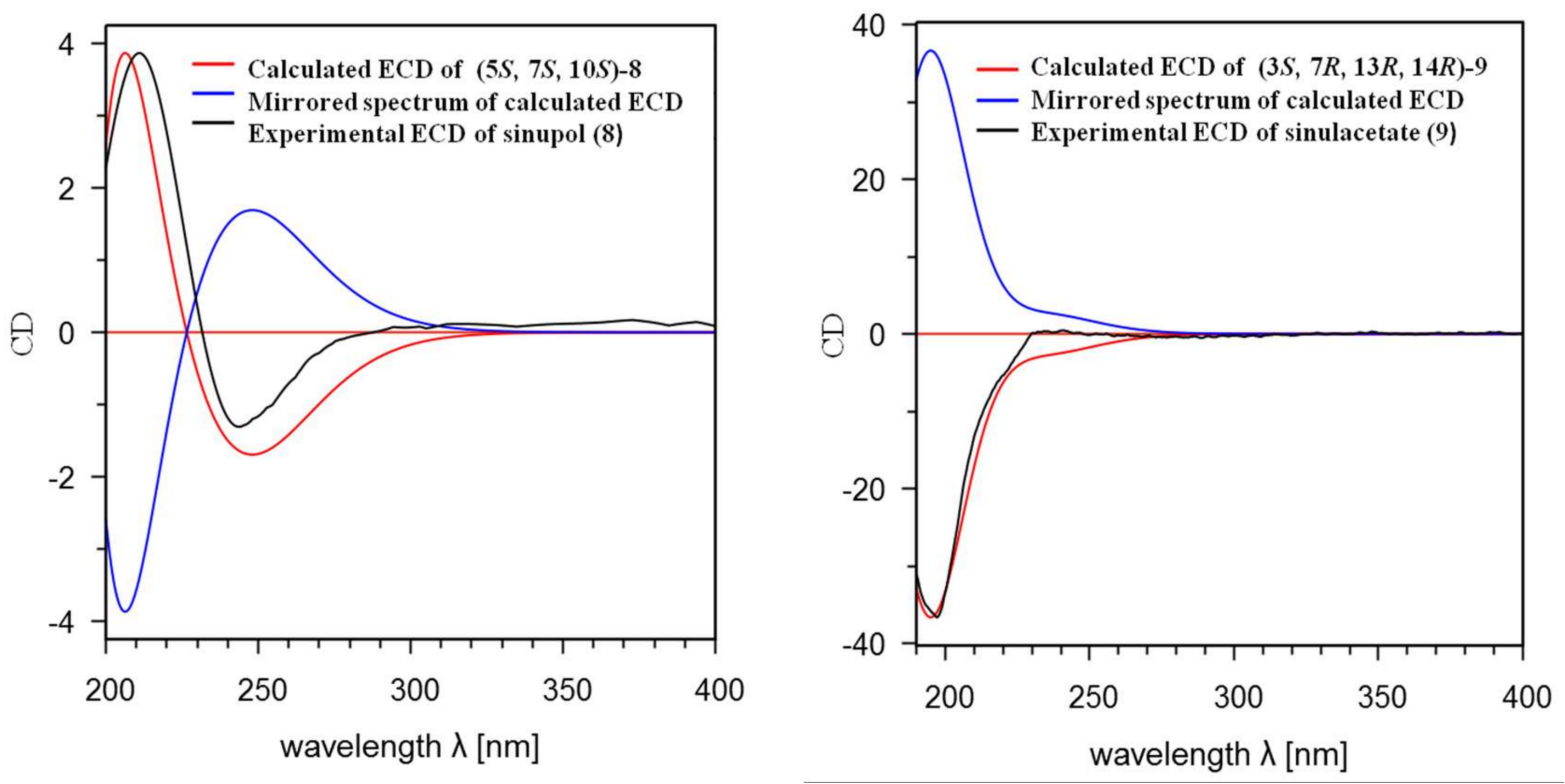
Experimental electronic circular dichroism (ECD) spectra of **8** and **9** (black), the calculated ECD spectra of **8** and **9** (red), and their enantiomers (blue), respectively.

With the establishment of the relative configurations of the chiral carbons of **8** and **9**, the remaining task was to determine their absolute configurations (ACs). Due to the minute amount of the new compounds and unsuccessful efforts on crystallization, a reliable and powerful approach, the time-dependent density functional theory electronic circular dichroism (TDDFT ECD) calculation was applied to establish their absolute configurations. As shown in Figure 4, the experimental ECD spectrum (MeCN) of **8** showed a negative cotton effect (CE) at 234 nm (Δε −1.98) and a positive CE at 211 nm (Δε +4.83). The experimental ECD spectrum (MeCN) of **9** showed a negative cotton effect (CE) at 198 nm (Δε −2.56). The recognizable files for Schrödinger LLC, 2015-4 [15] with the format of “mol2” were readily set out and then the initial torsional sampling (MCMM) and OPLS_2005 force field conformational searches of (5*S*, 7*S*, 10*S*)-**8** and (3*S*, 7*R*, *13R*, 14*R*)-**9** were carried out by the Macromodel in Schrödinger LLC, 2015-4 [Ligprep, force field OPLS_2005; generate possible states at targets PH 7.0 ± 2.0; conformational search, force field OPLS_2005; dielectric constant, 1.0; method, torsional sampling (MCMM); customize the search with torsion sampling options: intermediate, with maximum number of steps: 1000, use 100 steps per rotatable bond; energy window, 21 KJ/mol; eliminate redundant conformers with maximum atom deviation cutoff: 0.5 Å; others were the default parameters in Schrödinger LLC, 2015-4], which afforded 67 and 15 conformers, respectively. The Boltzmann populations of the conformers were obtained based on the potential energy provided by the OPLS_2005 force field, leading to 20 conformers for **8** and eight conformers for **9** above 1% population for further optimization [Supplementary Materials, Figures S1 and S3] with the output files format “sdf”. The above output files were transformed manually into the files with the format of “gjf” by text editor, which were recognizable for Gaussian 09 revision C.01 The re-optimization of the resulting geometries were all performed with Gaussian 09 revision C.01 [16] at the B3LYP/6-311G(d, p) level with IEFPCM (the Polarizable Continuum Model (PCM) using the integral equation formalism variant solvent model for MeCN # opt freq B3LYP/6-311g(d, p) scrf (solvent = acetonitrile), the input model used in the calculation was provided (Scheme S1)), and frequency analysis was performed as well to confirm that the re-optimized geometries were at the energy minima. Differences among all of the conformers for each compound were further illustrated through the figures with superimposed conformers (Figures S2 and S4). The specific splitting patterns and ^1^H-^1^H coupling constants for the most populated conformer of **9** were also compared with experimental ones and a high similarity was observed (Table S2 and Figure S5). The ECD spectra for all re-optimized geometries were then obtained by the TDDFT calculations with Gaussian 09 Revision C.01 ((B3LYP/6-311G (d, p) scrf (solvent = acetonitrile) td (nstates = 30, singlets), the input model used in the calculation was also provided (Scheme S2). Finally, SpecDis1.62 software was used to obtain the Boltzmann-averaged ECD spectra of (5*S*, 7*S*, 10*S*)-**8** and (3*S*, 7*R*, 13*R*, 14*R*)-**9**, which highly matched the experimental ones, whereas those of their enantiomers showed completely opposite curves (Figure 4). The calculated ECD spectrum for (5*S*, 7*S*, 10*S*)-**8** showed a positive and a negative CE at 208 nm and 243 nm, respectively, which were highly similar to the experimental results. Similarly, the calculated ECD curve for (3*S*, 7*R*, 13*R*, 14*R*)-**9** displayed a negative CE at 201 nm, indicating that had almost the same CE as the experimental result for **9**. Consequently, the absolute configurations of sinupol (**8**) and sinulacetate (**9**) were determined to be 5*S*, 7*S*,10*S* and 3*S*, 7*R*, 13*R*, 14*R*, respectively*.*

**Scheme 1:**
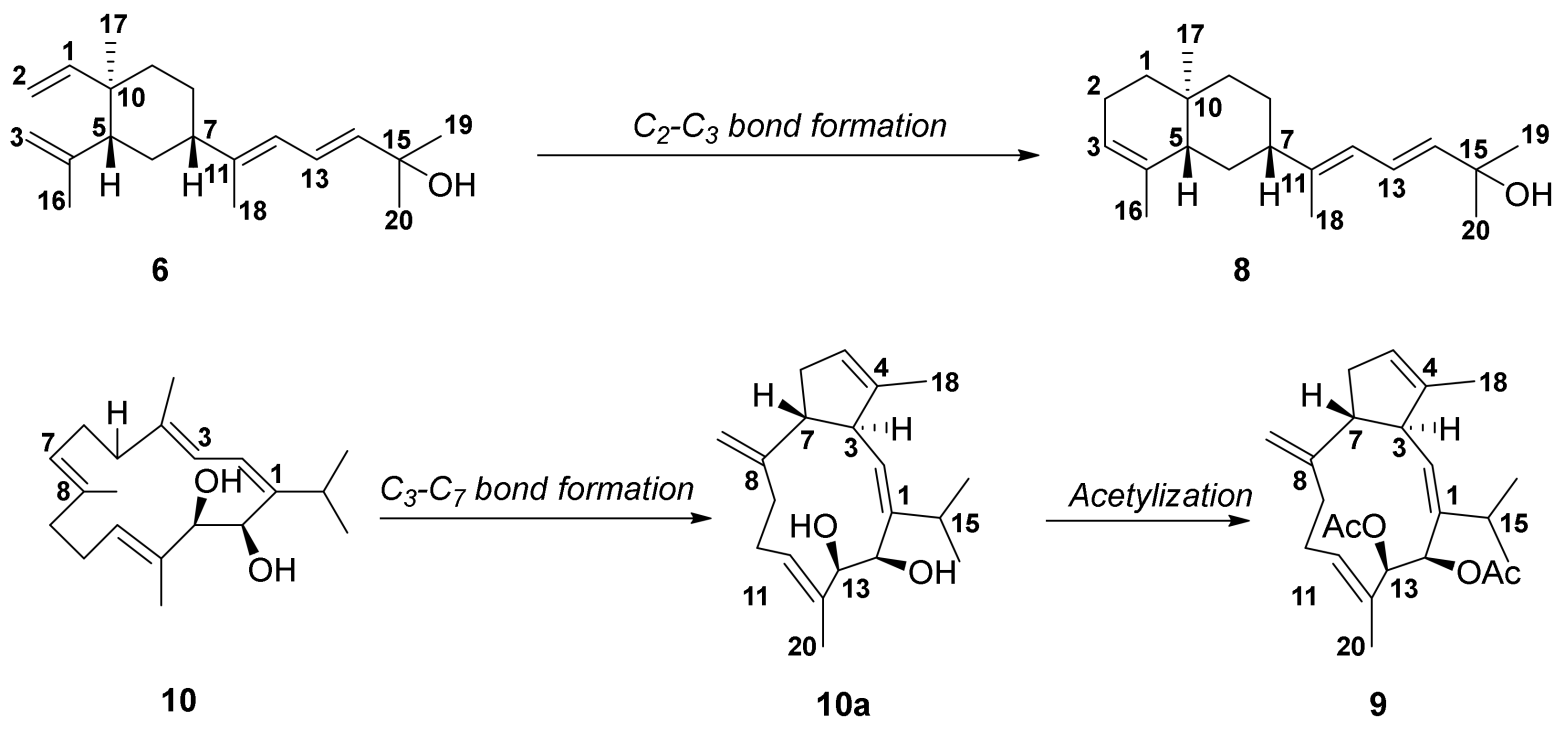
Proposed biosynthetic pathway of **8** and **9** from their parent precursors.

Although sinupol (**8**) and sinulacetate (**9**) formally displayed different carbon skeletons from the co-occurred fuscol (**6**) and sarcophytrol B (**10**), they were structurally related as they shared some common moieties, such as the conjugated diene chains (for **8** and **6**) as well as isopropyl groups and 14-membered rings (for **9** and **10**). To explain their possible biogenetic relationships, plausible biosynthetic pathways were proposed (Scheme 1). Compound **8** was proposed to be a cyclization product of fuscol (**6**). This hypothesis was also in agreement with the experimental results reported by Barry and co-workers [17]. A capnosane diterpenoid, sinulacetate (**9**), with 5:11-fused carbobicyclic skeleton, could be derived from the co-occurring cembranoid **10**. As outlined in Scheme 1, the formation of a C-C bond between C-3 and C-7 followed by acetylization at C-13 and C-14 generated **9**.

All of the isolates were evaluated for antibacterial and cytotoxic activities. Unfortunately, they were all inactive. Interestingly, in PTP1B (a recognized target for diabetes and obesity) inhibitory activity assay [18], compounds **7**, **8**, and **9** exhibited inhibitory activity with IC_50_ values of 75.5 μM, 63.9 μM, and 51.8 μM, respectively, as compared to the positive control of oleanolic acid (IC_50_ = 2.56 μM).

## 3. Discussion 

Although chemical investigation of the title animal growing in the Red Sea has been conducted extensively, only two sesquiterpenoids have been characterized from *S. polydactyla* growing in the South China Sea [19]. This is the second report about the chemical constituents produced by the title animal growing in the South China Sea, further elucidating four types of diterpenoids. Three novel diterpenes with an undescribed bicyclic[3.3.1]nonane nucleus have been isolated previously from *S. polydactyla* [3], while another two new diterpenoids belonging to capnosane and prenyleudesmane types, respectively, together with five previously described molecules with cembrane and lobane type compounds were reported in this paper, which indicates the rich chemical diversity and high complexity of metabolites from the title animal. To the best of our knowledge, this is also the first report about the capnosane diterpenoid isolated from *Sinularia* sp. The diterpenes of lobane and cembrane types could be the precursors of those with prenyleudesmane and capnosane skeletons, respectively. Further chemical correlations or synthetic studies should be conducted to verify this hypothesis. Interestingly, the new compounds showed moderate inhibitory activity against PTP1B. This finding regarding the chemical diversity and pharmaceutical potentials should stimulate chemists and biologists to conduct further studies. The evaluation of these compounds’ biological roles in the lifecycle of soft coral and their physiological functions should also be further investigated.

## 4. Materials and Methods

### 4.1. General Experimental Procedures

All of the chemicals were obtained from commercial sources. Circular dichroism (CD) spectra were measured on a JASCO J-810 instrument (JASCO, Tokyo, Japan). Optical rotations were measured on Autopol VI Polarimeter (Rudolph, NJ, USA). NMR spectra were measured on a Bruker DRX-400 or Bruker DRX-500 spectrometer (BrukerBiospin AG, Fällanden, Germany). Chemical shifts (*δ*) are reported in ppm with reference to the solvent signals, and coupling constants (*J*) are in Hz. MS spectra were recorded on a Finnigan-MAT-95 mass spectrometer (FinniganMAT, San Jose, CA, USA). Commercial silica gel (Qingdao Haiyang Chemical Group Co., Ltd., Qingdao, China, 200–300 and 400–600 mesh), and Sephadex LH-20 gel (Amersham Biosciences, Little Chalfont, UK) were used for column chromatography, and precoated silica gel plates (Yan Tai Zi Fu Chemical Group Co., Yantai, China, G60 F-254) were used for analytical TLC (thin layer chromatography). Reversed-phase (RP) HPLC was performed on an Agilent 1260 series liquid chromatography equipped with a DAD G1315D detector at 210 and 254 nm. A semi-preparative ODS-HG-5 column (5 µm, 250 × 9.4 mm) was employed for the purifications. All solvents used for CC and HPLC were of analytical grade (Shanghai Chemical Reagents Co., Ltd., Shanghai, China) and chromatographic grade (Dikma Technologies Inc., Lake Forest, CA, USA), respectively.

### 4.2. Biological Materials

The biological samples of *Sinularia polydactyla* were collected by SCUBA diving at Xisha Islands, Hainan province, People’s Republic of China, in October 2013, and identified by Professor Hui Huang of the South China Sea Institute of Oceanology, Chinese Academy of Sciences. The biological material was frozen immediately after collection. A voucher specimen (NO. 13-XS-23) was deposited at the Shanghai Institute of Materia Medica, Chinese Academy of Sciences.

### 4.3. Isolation of Diterpenoids from *Sinularia Polydactyla*

Pieces of the freshly collected title animal (430 g, dry weight) were extracted exhaustively with acetone at room temperature (6 × 2.0 L). The organic extract was evaporated to give a residue, which was successively partitioned between Et_2_O and H_2_O, *n*-BuOH and H_2_O. The Et_2_O solution was concentrated under reduced pressure to give a dark brown residue (13.8 g), which was subjected to silica gel column chromatography and eluted with petroleum ether-Et_2_O (0–100% Et_2_O in petroleum ether (PE), gradient), yielding 11 fractions (*Frs.* 1–11). Fraction 6 (eluent: PE/Et_2_O, 9:1; components with an *R*_f_ from 0.3 to 0.5 (elution solvent for TLC, PE/Et_2_O = 1:1)) was then eluted with a gradient PE-Et_2_O (19:1 to 1:1, gradient) to yield four sub-fractions (6A–6D). Sub-fraction 6C (eluent: PE/Et_2_O, 7:3; *R*_f_ around 0.4 (elution solvent for TLC, PE/Et_2_O = 1:1)) was further purified using RP-HPLC (MeOH/H_2_O (90:10), 3.0 mL/min) to give compound **6** *^a^* (4.7 mg, *t*_R_ 13.4 min). Fraction 7 (eluent: PE/Et_2_O, 6:4 to 4:6; components with an *R*_f_ value from 0.3–0.8 (elution solvent for TLC, PE/Et_2_O = 3:7)) was subjected to Sephadex LH-20 CC (PE/CH_2_Cl_2_/MeOH, 2:1:1) to give five sub-fractions (7A–7E). Sub-fraction 7C (with an *R*_f_ value from 0.5 to 0.7 (elution solvent for TLC, PE/Et_2_O = 3:7)) was separated by RP-HPLC (MeCN/H_2_O (85:15), 3.0 mL/min) to give compounds **5** *^a^* (2.1 mg, *t*_R_ 17.6 min) and **8** *^a^* (2.4 mg, *t*_R_ 14.5 min). Similarly, sub-fraction 7D (with an *R*_f_ value around 0.7 (elution solvent for TLC, PE/Et_2_O = 3:7)) was purified by RP-HPLC (MeOH/H_2_O (90:10), 3.0 mL/min) to give compound **9** *^b^* (0.9 mg), and sub-fraction 7E (with an *R*_f_ value from 0.3 to 0.5 (elution solvent for TLC, PE/Et_2_O = 3:7)) was chromatographed by RP-HPLC (MeOH/H_2_O (70:30), 3.0 mL/min) to give compounds **4** *^c^* (3.9mg, *t*_R_ 15.6 min), **7** *^a^* (3.7 mg, *t*_R_ 12.3 min), and **10** *^d^* (3.5mg, *t*_R_ 18.3 min), respectively. (Note: *^a^* spots could be detected on the TLC under UV light (254 nm) and displayed blue through heating after spraying with anisaldehyde H_2_SO_4_ reagent; *^b^* spots displayed purple through heating after spraying with anisaldehyde H_2_SO_4_ reagent; *^c^* spots displayed blue through heating after spraying with anisaldehyde H_2_SO_4_ reagent; *^d^* spots could be detected on the TLC under UV light (254 nm) and displayed purple through heating after spraying with anisaldehyde H_2_SO_4_ reagent).

Compound **8**: colorless oil; [α${]}_{D}^{25}$+13.1 (*c* 0.1, CH_3_OH); IR (KBr): 3390, 1620, 834 cm^−1^; UV λ_max_ (logε) 255 (3.5) nm; ^1^H and ^13^C NMR (C_5_D_5_N, 500 and 125 MHz), see Table 1 and Supplementary Materials; (+)-HREIMS *m*/*z* 288.2454 [M]*^+^* (calcd. for C_20_H_32_O, *m*/*z* 288.2453).

Compound **9**: colorless oil; [α${]}_{D}^{25}$ −48.5 (*c* 0.1, CHCl_3_); IR (KBr): 1751, 1748, 883, 801 cm^−1^; ^1^H and ^13^C NMR (CDCl_3_, 500 and 125 MHz), see Table 1 and Supplementary Materials; (+)-HREIMS *m*/*z* 386.2449 [M]^+^ (calcd. for C_24_H_34_O_4_, *m*/*z* 386.2457).

### 4.4. Determination of the Absolute Configurations of Compounds **8** and **9** by TDDFT ECD Calculations

Conformational searches were carried out using the torsional sampling (MCMM) method and OPLS_2005 force field. Conformers above 1% population were re-optimized at the B3LYP/6-311G (d, p) level with IEFPCM (Polarizable Continuum Model using the Integral Equation Formalism variant) solvent model for acetonitrile [15]. For the resulting geometries, ECD spectra were obtained by TDDFT calculations performed with the same functional basis set and solvent model as the energy optimization. Finally, the Boltzmann-averaged ECD spectra of the two compounds were obtained with SpecDis1.62 [20].

### 4.5. Bioactivity Assay

Recombinant PTP1B catalytic domain was expressed and purified according to a previous report [16]. The enzymatic activities of the PTP1B catalytic domain were determined at 30 °C by monitoring the hydrolysis of *p*NPP. The dephosphorylation of *p*NPP generated product *p*NP, which was monitored at an absorbance of 405 nm by the EnVision multi label plate reader (Perkin Elmer Life Sciences, Boston, MA). In an atypical 100 *µ*L assay mixture containing 50 mmol/L 3-[*N*-morpholino] propanesulfonic acid (MOPs), pH 6.5, 2 mmol/L *p*NPP, and 30 nmol/L recombinant PTP1B, activities were continuously monitored and the initial rate of hydrolysis was determined using the early linear region of the enzymatic reaction kinetic curve. The IC_50_ was calculated with Prism 4 software (Graphpad, San Diego, CA). Compounds **5**–**10** were tested in vitro and compounds **5**, **6**, and **10** were inactive. Interestingly, **7**–**9** exhibited moderate inhibitory activity against PTP1B with IC_50_ values 72.4 μM, 63.9 μM, and 51.8 μM, respectively, with the positive control of oleanolic acid (IC_50_ = 2.56 μM).

## Figures and Tables

**Table 1:** ^1^H (500 MHz) and ^13^C (125 MHz) NMR data (*δ* in ppm, *J* in Hz) for compounds **8** and **9**.

Position	8		9	
	*δ*_H_ *^a^*	*δ*_C_ *^a^*	*δ*_H_ *^b^*	*δ*_C_ *^b^*
1	1.33, m	38.1	-	140.9
2	1.97, m 2.10, m	23.2	5.09, d (9.5)	132.9
3	5.35, brs	121.2	3.48, t (9.5)	49.8
4	-	135.9	-	142.1
5	1.90,dd (12.7, 1.1)	46.8	5.34, t (3.5)	123.9
6α 6β	1.28, ddd (12.7, 11.0, 10.4) 1.76, dt (11.0, 1.1)	29.0	2.22, m 2.17, m	37.1
7	2.02, td (10.4, 3.9)	48.9	2.69, dt (9.5, 8.9)	58.2
8	1.53, m 1.62, m	26.8	-	150.5
9	1.40, dt (12.9, 3.9) 1.16, dt (12.9, 4.2)	40.3	2.19, m	28.9
10	-	32.4	2.22, m	28.7
11	-	142.2	5.65, dd (6.5, 10.1)	129.7
12	6.17, d (11.2)	123.8	-	132.5
13	6.96, dd (11.2, 15.3)	123.0	4.95, d (10.2)	77.1
14	6.13, d (15.3)	141.9	6.02, d (10.2)	73.6
15	-	69.9	2.50, m	28.6
16	1.63, s	21.2	1.08, s	23.7
17	0.84, s	15.7	1.03, s	26.4
18	1.78, s	14.9	1.55, s	15.5
19	1.59, s	30.9	4.87, brs 4.89, brs	113.3
20	1.59, s	30.9	1.66, s	17.8
13-OAc (CO)	-	-	-	170.5
13-OAc (CH_3_)	-	-	2.01, s	21.3
14-OAc (CO)	-	-	-	169.8
14-OAc (CH_3_)	-	-	2.05, s	21.3

*^a^* Means measured in C_5_D_5_N; *^b^* means measured in CDCl_3_.

**Table 2:** Comparison of multiplicity and coupling constants of H-3 and H-7 in **9** and **12**–**14**.

	9	12	13	14
	*δ*_H_, mult. (*J*, Hz)	*δ*_H_, mult. (*J*, Hz)	*δ*_H_, mult. (*J*, Hz)	*δ*_H_, mult. (*J*, Hz)
H-3	3.48 t (9.5)	2.48 dd (11.0, 9.5)	2.45 dd (11.6, 5.4)	2.41 dd (11.3, 5.8)
H-7	2.69 dt, (9.5, 8.9)	2.38 dt (11.0, 8.0)	2.97 dt (5.4, 11.3)	2.50 dt (5.8, 11.7)

NMR data were recorded in CDCl_3._

## Supplementary Materials

The following are available online at http://www.mdpi.com/1660-3397/16/4/103/s1, ^1^H and ^13^C NMR spectra of **8** and **9**, and 2D (COSY, HSQC, HMBC, NOESY) NMR spectra of **8** and **9**.

## References

[1] Chen W.-T., Li Y., Guo Y.-W. (2012). Terpenoids of *Sinularia* soft corals: Chemistry and bioactivity. Acta Pharm. Sin. B.

[2] Liang L.-F., Li Y.-F., Liu H.-L., Guo Y.-W. (2013). Research advance on the chemistry and bioactivity of secondary metabolites from the soft corals of the genus *Sinularia*. J. Int. Pharm. Res..

[3] Ye F., Zhu Z.-D., Chen J.-S., Li J., Gu Y.-C., Zhu W.-L., Li X.-W., Guo Y.-W. (2017). Xishacorenes A-C, diterpenes with bicyclo[3.3.1]nonane nucleus from the Xisha soft coral Sinularia polydactyla. Org. Lett..

[4] Liang L.-F., Kurtán T., Mándi A., Gao L.-X., Li J., Zhang W., Guo Y.-W. (2014). Sarsolenane and capnosane diterpenes from the Hainan soft coral *Sarcophyton trocheliophorum* Marenzeller as PTP1B inhibitors. Eur. J. Org. Chem..

[5] Liang L.-F., Lan L.-F., Orazio T.-S., Guo Y.-W. (2013). Sartrolides A-G and bissartrolide, new cembranolides from the South China Sea soft coral *Sarcophyton trocheliophorum* Marenzeller. Tetrahedron.

[6] Jia R., Kurtán T., Mándi A., Yan X.-H., Zhang W., Guo Y.-W. (2013). Biscembranoids formed from an α, β-Unsaturated γ-lactone ring as a dienophile: Structure revision and establishment of their absolute configurations using theoretical calculations of electronic circular dichroism spectra. J. Org. Chem..

[7] Cai Y.-S., Yao L.-G., Antonio D.P., Carlo I., Ernesto M., Orazio T.-S., Guo Y.-W. (2013). Polyoxygenated diterpenoids of the eunicellin-type from the Chinese soft coral *Cladiellakrempfi*. Tetrahedron.

[8] Liang L.-F., Kurtán T., Mándi A., Yao L.-G., Li J., Zhang W., Guo Y.-W. (2013). Unprecedented diterpenoids as a PTP1B inhibitor from the Hainan soft coral *Sarcophyton trocheliophorum* Marenzeller. Org. Lett..

[9] Li L., Sheng L., Wang C.-Y., Zhou Y.-B., Huang H., Li X.-B., Li J., Ernesto M., Margherita G., Guo Y.-W. (2011). Diterpenes from the Hainan soft coral *Lobophytum cristatum* Tixier-Durivault. J. Nat. Prod..

[10] Angelie E.R., Proksch P., Wray V., Witte L., Ofwegen L.V. (1998). Four new bioactive lobane diterpenes of the soft coral *Lobophytum pauciflorum* from Mindoro, Philippines. J. Nat. Prod..

[11] Coll J.C., Bowden B.F., König G.M., Braslau R., Price I.R. (1986). Studies of Australian soft corals. XXXX. The natural products chemistry of alcyonacean soft corals with special reference to the genus Lobophytum. Bull. Soc. Chim. Belg..

[12] Kobayashi M., Osabe K. (1989). Marine terpenes and terpenoids. VIII. Transannular cyclization of 3,4-epoxy-1,7,11-cembratriene system. Chem. Pharm. Bull..

[13] Liu Z., Cheng W., Liu D., Ofwegen L.V., Proksch P., Lin W.-H. (2014). Capnosane-type cembranoids from the soft coral *Sarcophyton trocheliophotrum* with antibacterial effects. Tetrahedron.

[14] Kobayashi M., Nakagawa T., Mitsuhashi H. (1979). Marine terpenes and terpenoids. I. Structures of four cembrane-type diterpenes; sarcophytol-A, sarcophytol-A acetate sarcophytol-B, and sarcophytonin-A, from the soft coral, *Sarcophyton glaucum*. Chem. Pharm. Bull..

[15] MacroModel.

[16] Frisch M.J., Trucks G.W., Schlegel H.B., Scuseria G.E., Robb M.A., Cheeseman J.R., Scalmani G., Barone V., Mennucci B., Petersson G.A. (2009). Gaussian 09.

[17] Williams F., Guo Q.-X. (1989). ESR evidence for the stereospecific formation of the chair cyclohexane- 1,4-diyl radical cation from both bicyclo[2.2.0]hexane and 1,5-hexadiene. J. Am. Chem. Soc..

[18] Yang X., Li J., Zhou Y., Shen Q., Chen J., Li J. (2005). Discovery of novel inhibitor of human leukocyte common antigen-related phosphatase. Biochim. Biophys. Acta Gen. Subj..

[19] Zhang C.-X., Zhu C.-C., Yan S.-J., Li J., Su J.-Y., Liang Y.-J., Yang X.-P., Zheng K.-C., Zeng L.-M. (2008). Two new sesquiterpenoids from the soft coral *Sinulariapolydactyla* (Ehreberg). J. Asian Nat. Prod. Res..

[20] Bruhn T., Schaumloffel A., Hemberger Y., Bringmann G. (2013). SpecDis: Quantifying the comparison of calculated and experimental electronic circular dichroism spectra. Chirality.

