# Supplementary material for: Further New Diterpenoids as PTP1B Inhibitors from the Xisha Soft Coral Sinularia polydactyla
Source: Mar Drugs. 2018 Mar 25;16(4):103. doi: 10.3390/md16040103 (PMC5923390; doi:10.3390/md16040103)
Supplement: Supplementary file 1 [file marinedrugs-16-00103-s001.docx]

**Further New Diterpenoids as PTP1B Inhibitors from the Xisha Soft Coral** ***Sinularia polydactyla***

Fei Ye ^1,2^, Zheng-Dan Zhu ^1,2^, Yu-Cheng Gu ^3^, Jia Li ^1,2,4^, Wei-Liang Zhu ^1,2,4^ and Yue-Wei Guo ^1,2,4,^*

^1^ State Key Laboratory of Drug Research, Shanghai Institute of MateriaMedica, Chinese Academy of Sciences, 555 Zu Chong Zhi Road, Zhangjiang Hi-Tech Park, Shanghai 201203, China; simmyefei@simm.ac.cn (F.Y.); zdzhu@simm.ac.cn (Z.-D.Z.); jli@simm.ac.cn (J.L.);
wlzhu@simm.ac.cn (W.-L.Z.)

^2^ University of Chinese Academy of Sciences, No. 19A Yuquan Road, Beijing 100049, China

^3^ Syngenta, Jealott’s Hill International Research Centre, Bracknell, Berkshire RG42 6EY, UK; yucheng.gu@syngenta.com

^4^ Open Studio for Druggability Research of Marine Lead Compounds, Qingdao National Laboratory for Marine Science and Technology, 1 Wenhai Road, Aoshanwei, Jimo, Qingdao 266237, China

***** Correspondence: ywguo@simm.ac.cn; Tel.: +86-21-50805813

**Table of contents**

1. Original spectra data for compounds **8** and **9** 3

1.1 NMR spectra for compound **8** 3

1.2 HR-EIMS spectrum of compound **8**9

1.3 NMR spectra for compound **9** 10

1.4 HR-EIMS spectrum of compound **9** 16

2. Computational section 17

1. Original spectra data for compounds **8** and **9**

1.1 NMR spectra for compound **8**

^1^H NMR spectrum(500 MHz) for compound **8** in C_5_D_5_N

**
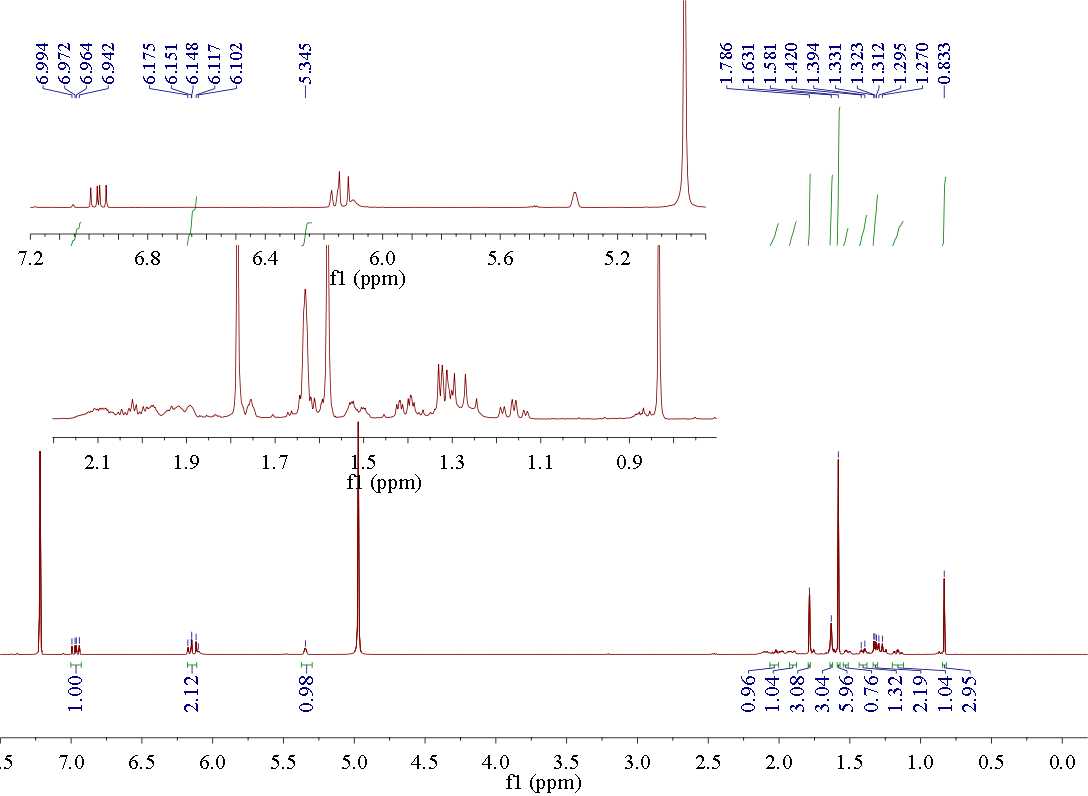
**

^13^C NMR spectrum(125 MHz) for compound **8** in C_5_D_5_N


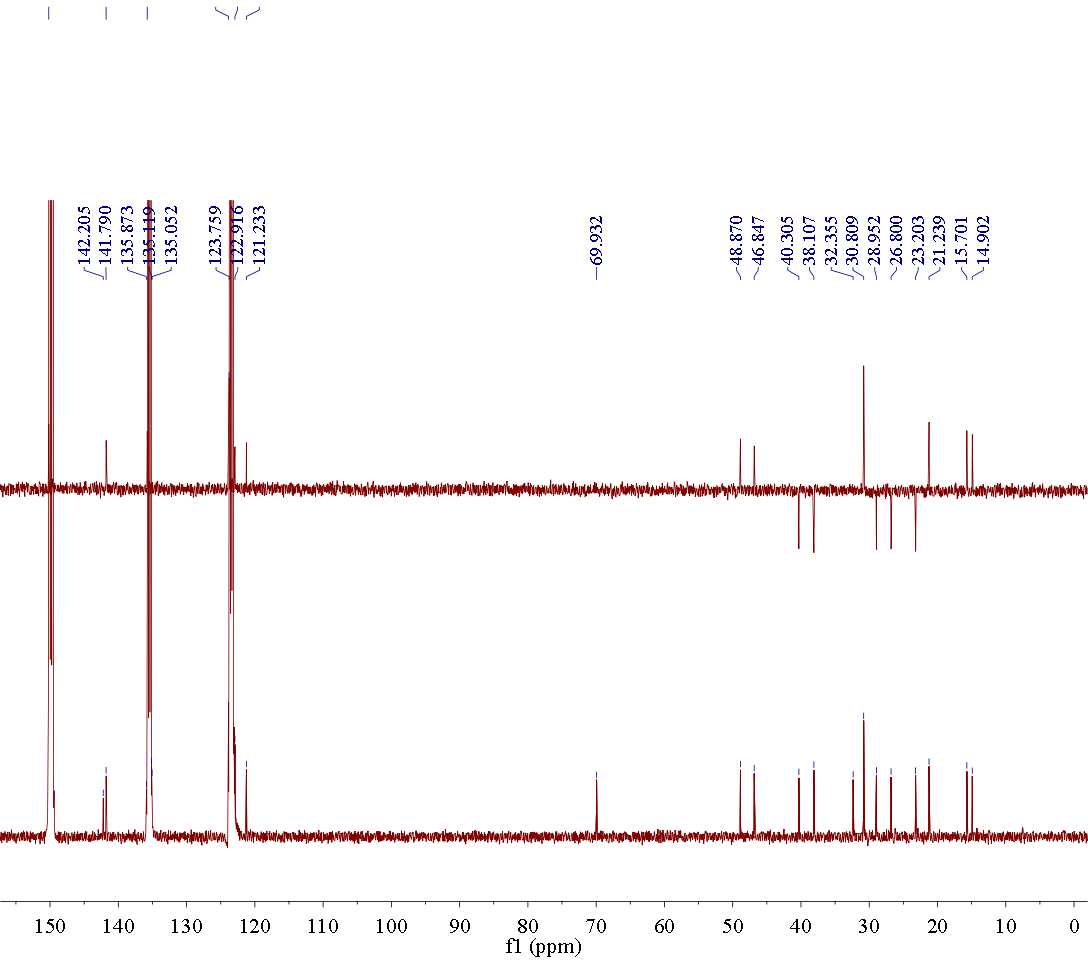


HSQC spectrum(500 MHz) for compound **8** in C_5_D_5_N


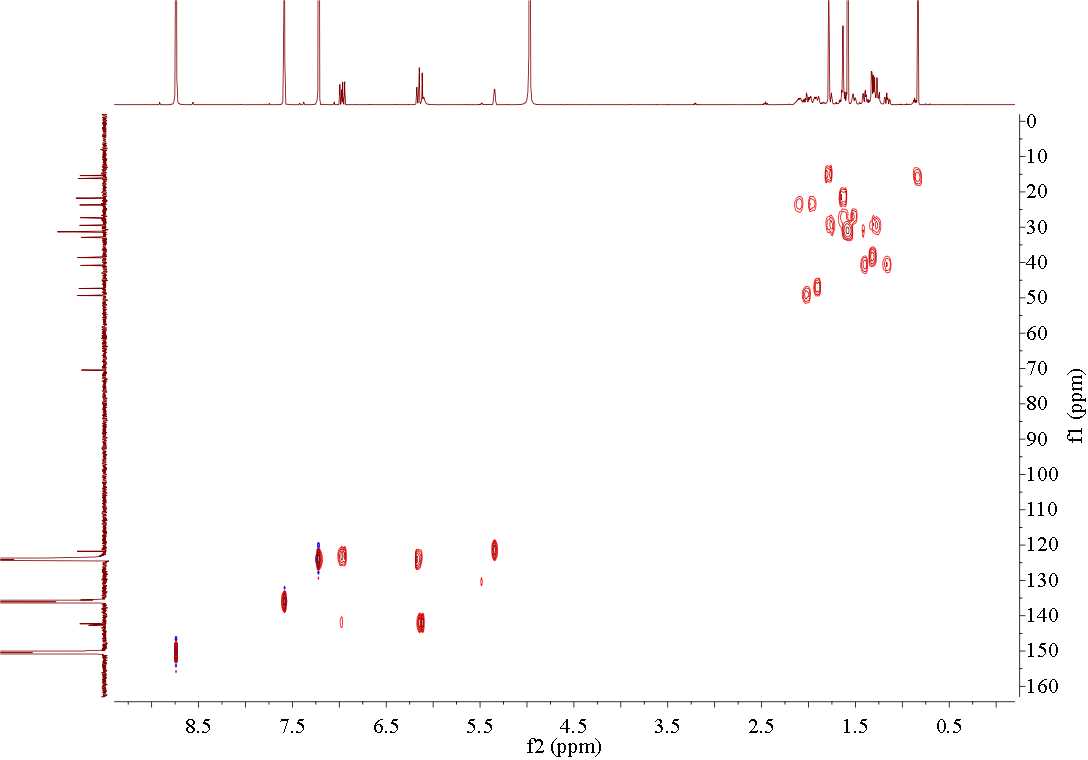


^1^H-^1^H COSY spectrum(500 MHz) for compound **8** in C_5_D_5_N


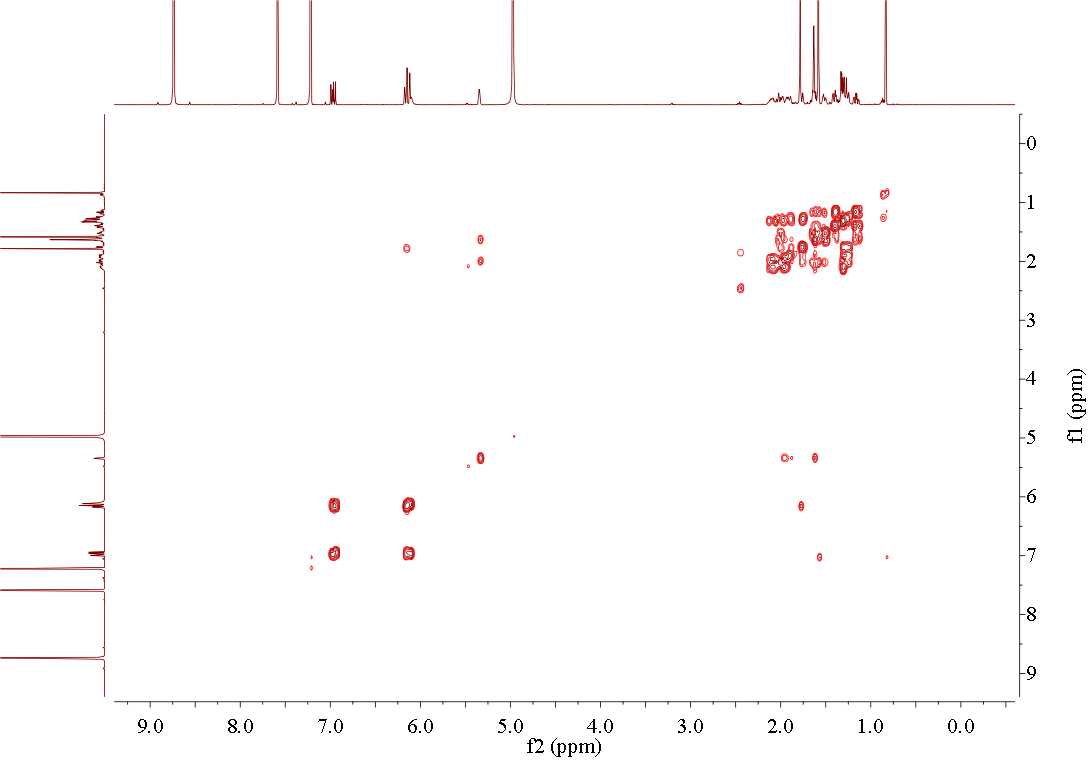


HMBC spectrum(500 MHz) for compound **8** in C_5_D_5_N


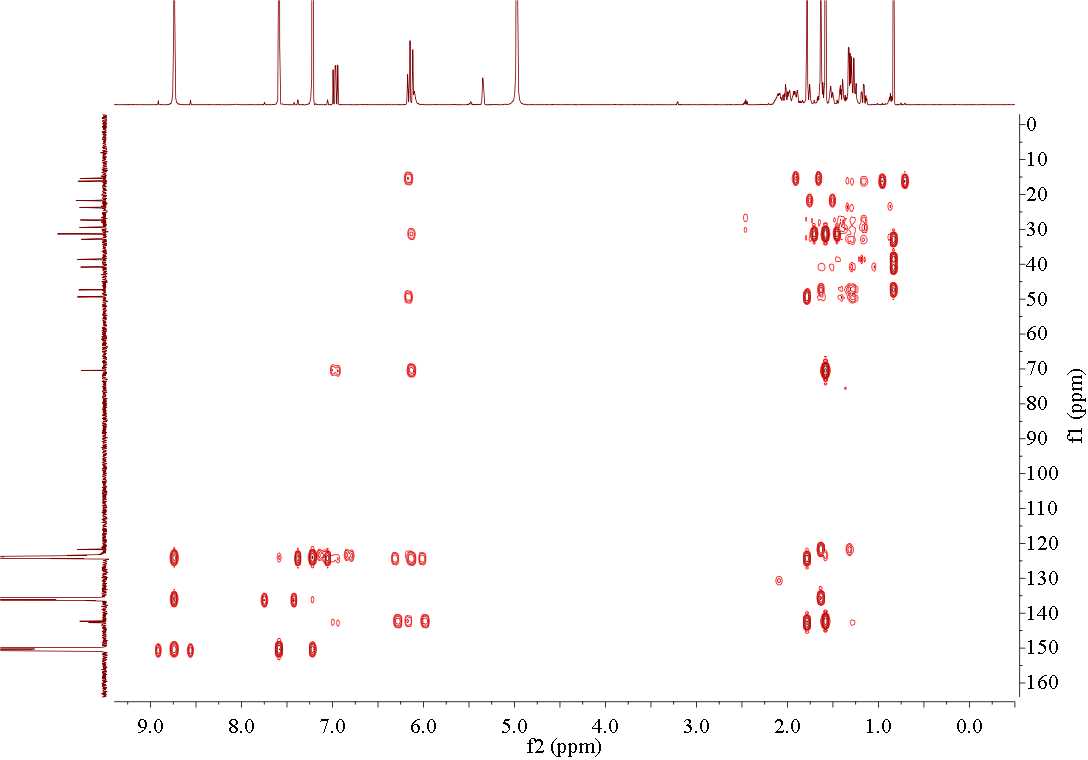


ROESY spectrum(500 MHz) for compound **8** in C_5_D_5_N


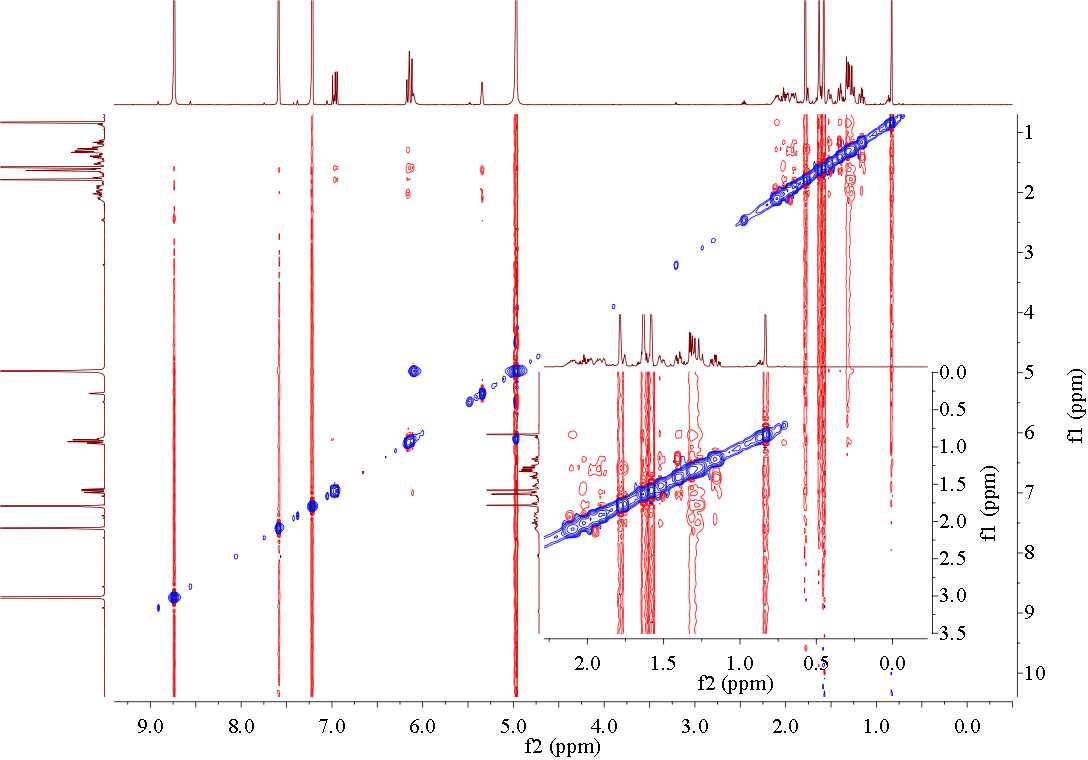


1.2 HR-EIMS spectrum of compound **8**

**
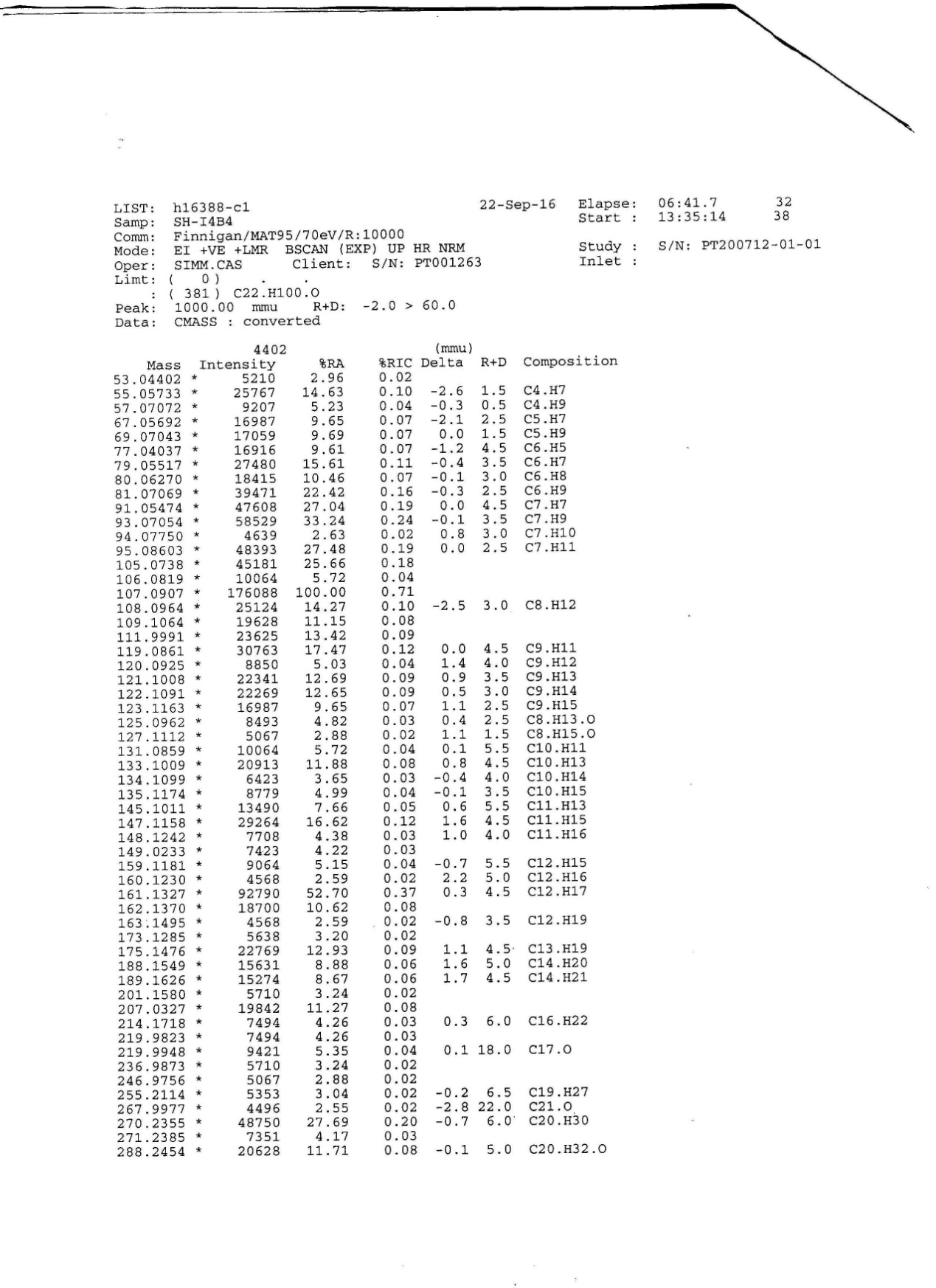
**

1.3 NMR spectra for compound **9**

^1^H NMR spectrum (500 MHz) for compound **9** in CDCl_3_


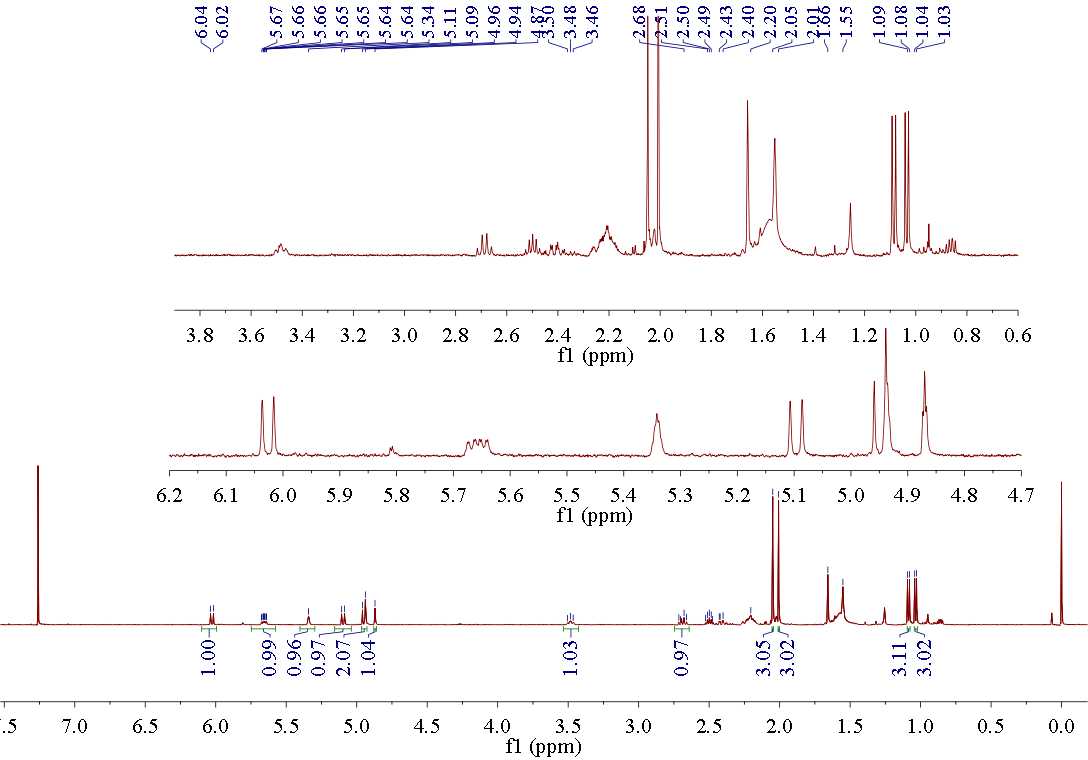


^13^C NMR spectrum(125 MHz) for compound **9** in CDCl_3_


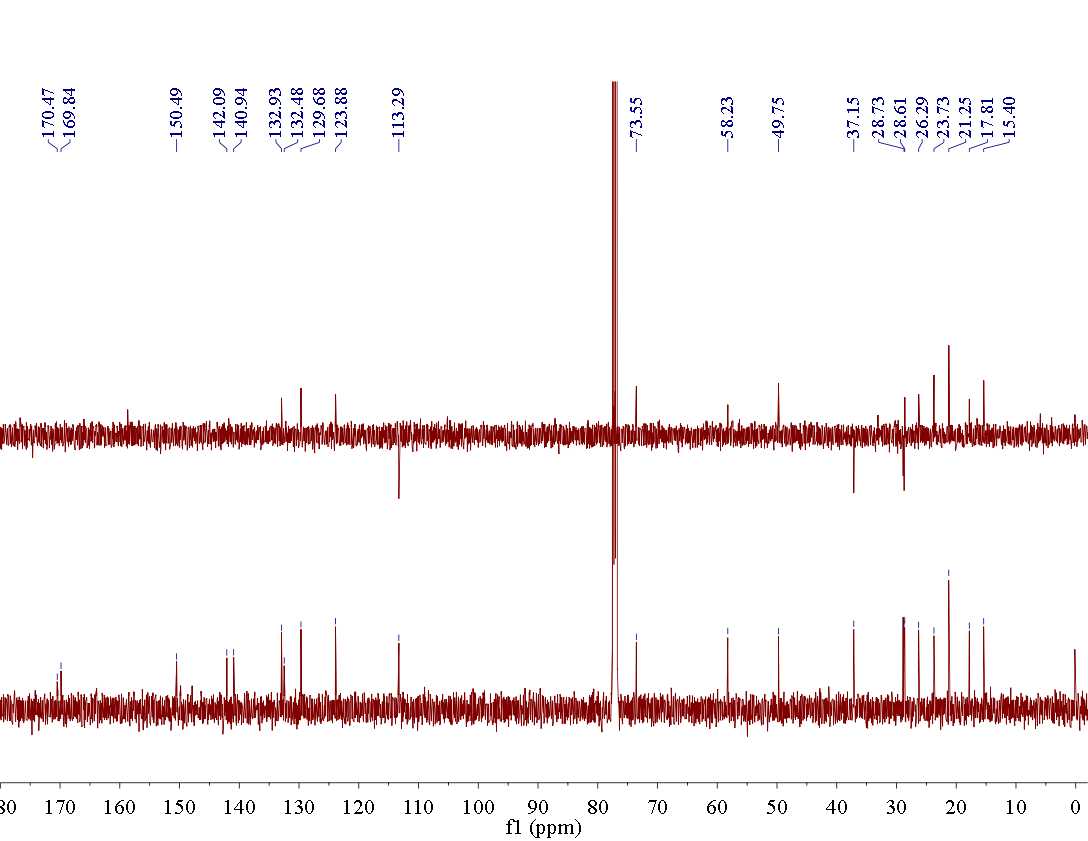


HSQC spectrum (500 MHz) for compound **9** in CDCl_3_

**
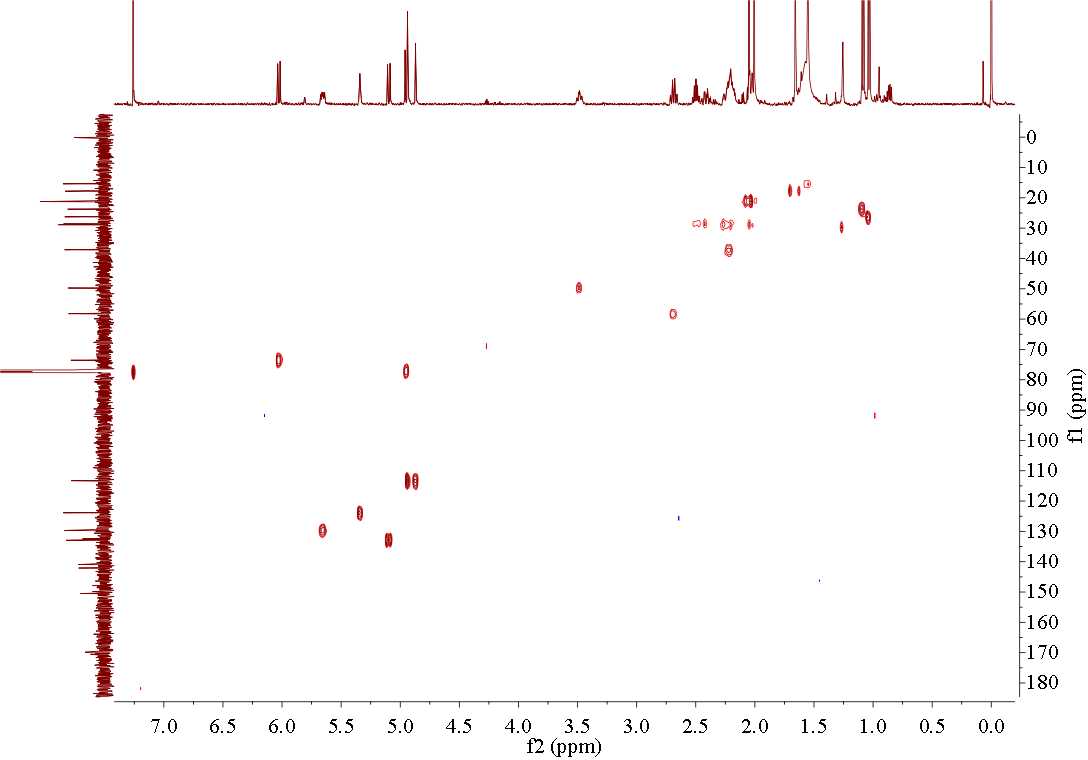
**

^1^H-^1^H COSY spectrum(500 MHz) for compound **9** in CDCl_3_

_
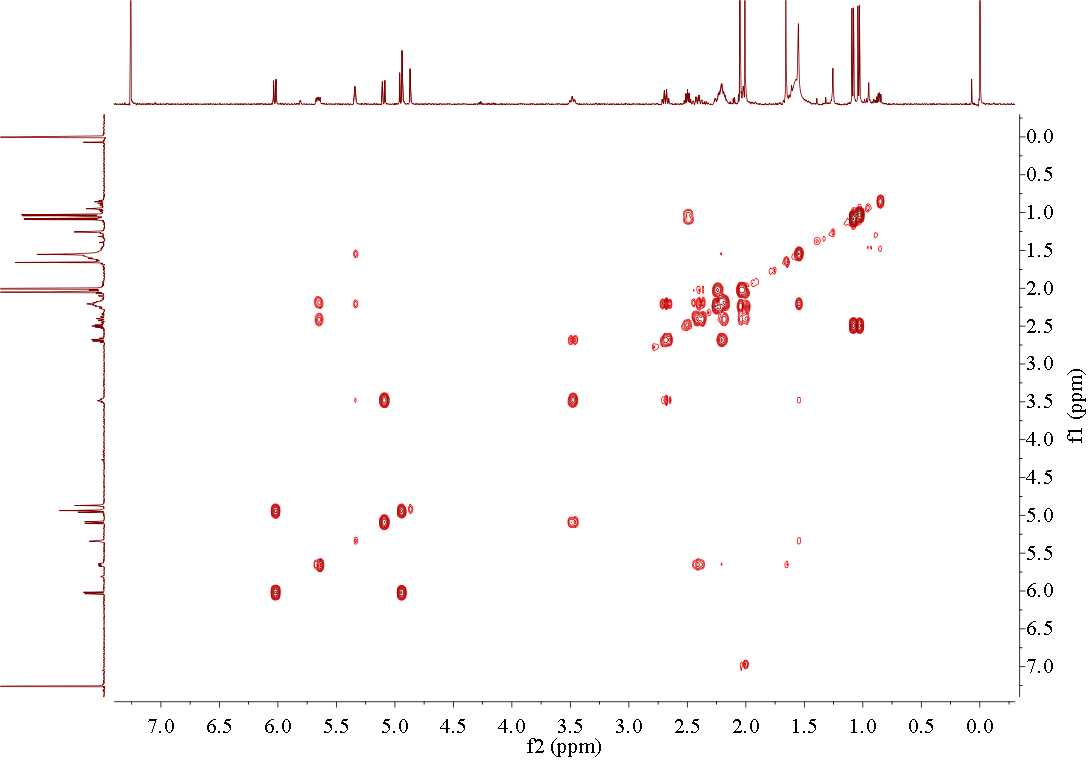
_

HMBC spectrum(500 MHz) for compound **9** in CDCl_3_

_
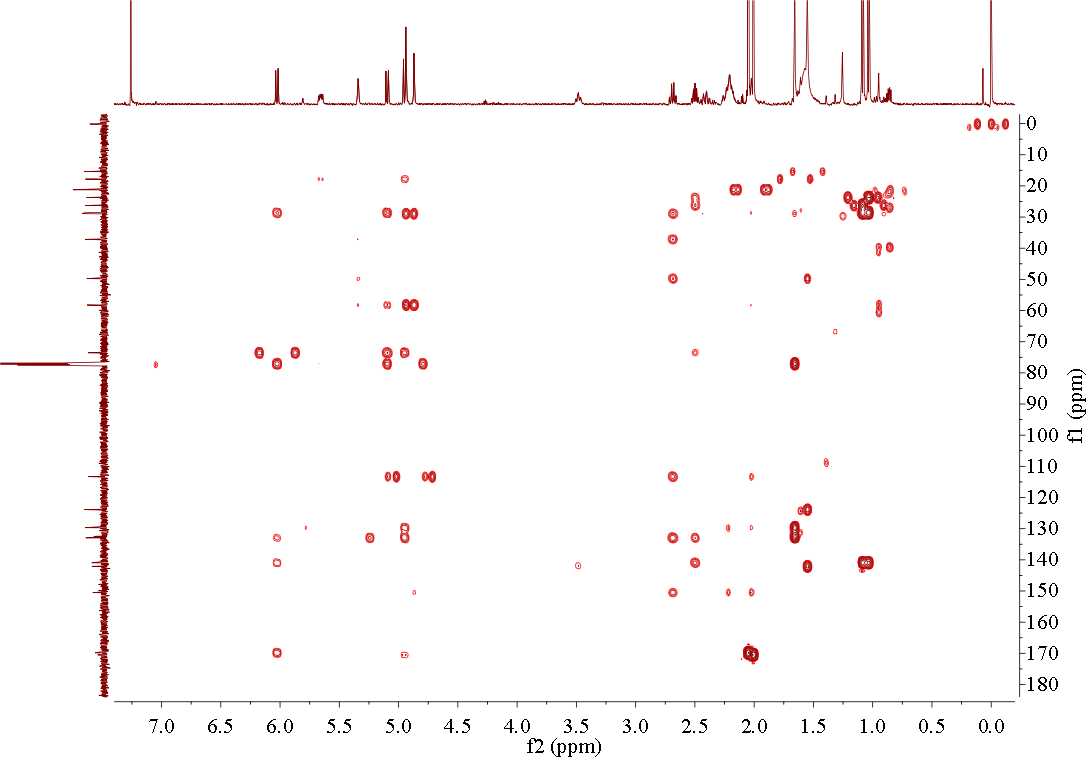
_

ROESY spectrum(500 MHz) for compound **9** in CDCl_3_

_
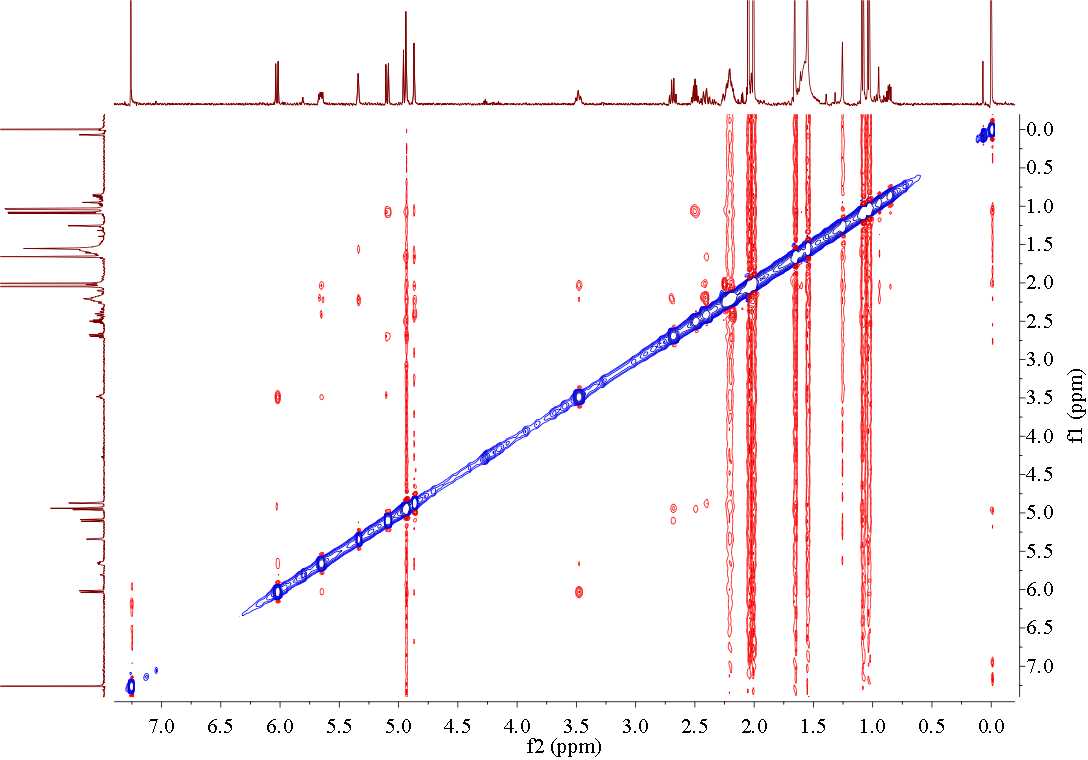
_

1.4 HR-EIMS spectrum of compound **9**


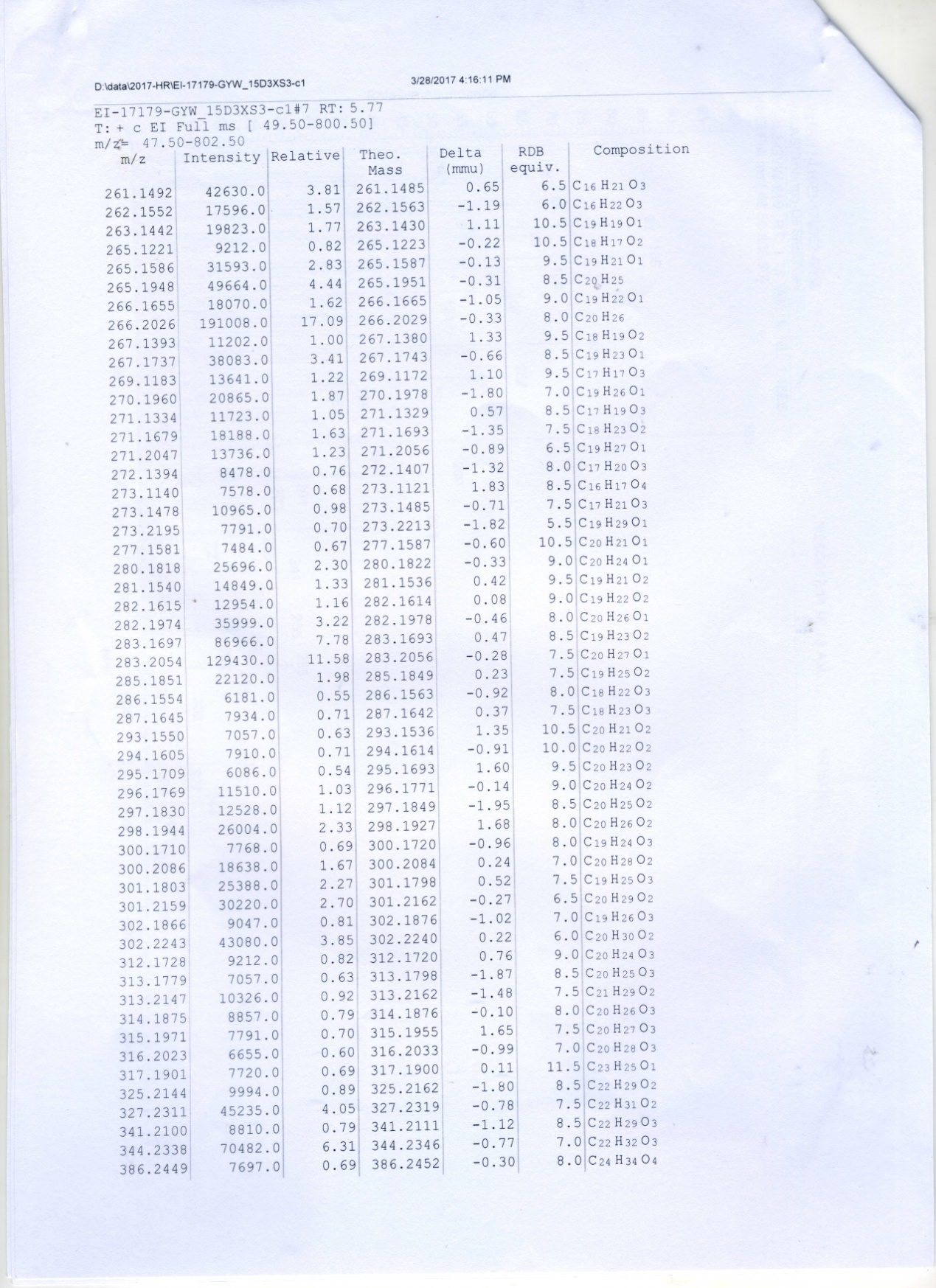


2. Computational section

Conformational searches were carried out using the torsional sampling (MCMM) method and OPLS_2005 force field. Conformers above 1% population were re-optimized at the B3LYP/6-311G(d,p) level with IEFPCM (Polarizable Continuum Model using the Integral Equation Formalism variant) solvent model for acetonitrile. For the resulting geometries, ECD spectra were obtained by TDDFT calculations performed with the same functional, basis set and solvent model as the energy optimization. Finally, the Boltzmann-averaged ECD spectra of the three compounds were obtained with SpecDis1.62.

Torsional sampling (MCMM) conformational searches using OPLS_2005 force field were carried out by means of the conformational search module in the Macromodel 9.9.223 software applying an energy window of 21 kJ/mol, which afforded 67 conformers for compound **8** and 10 conformers for compound **9**, respectively. The Boltzmann populations of the conformers were obtained based on the potential energy provided by the OPLS_2005 force field, which afforded 20 conformers for compound **8** and 5 conformers for compound **9** above 1% population for re-optimization. The re-optimization and the following TDDFT calculations of the re-optimized geometries (**Fig S1-S2, Table S1-S3**) were all performed with Gaussian 09^3^ at the B3LYP/6-311G(d,p) level with IEFPCM solvent model for acetonitrile. Frequency analysis was performed as well to confirm that the re-optimized geometries were at the energy minima. Finally, the SpecDis1.62 software was used to visualize the results.


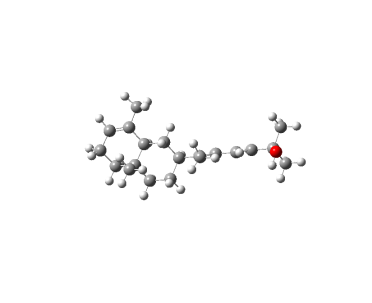

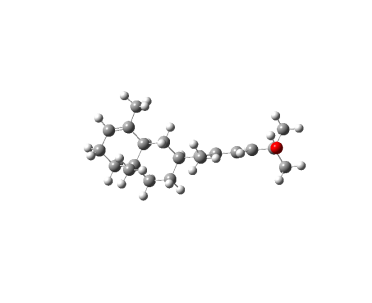

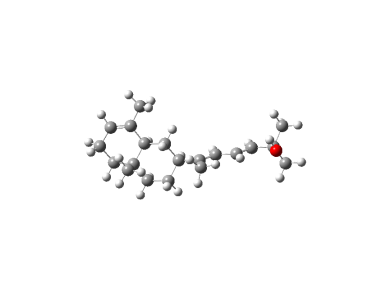


Conf. 1 Conf. 2 Conf. 3

18.81% 18.35% 17.67%


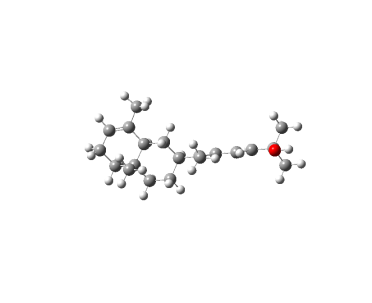

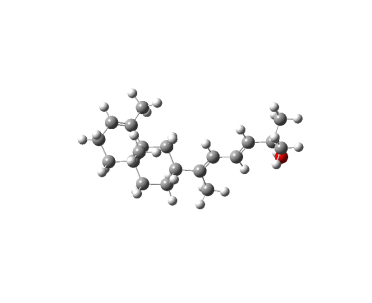

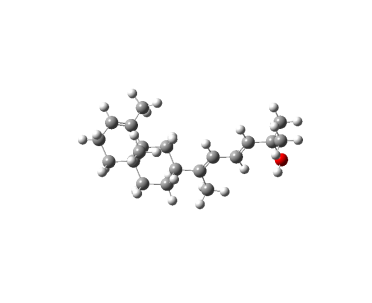


Conf. 4 Conf. 5 Conf. 6

7.18% 6.76% 6.73%


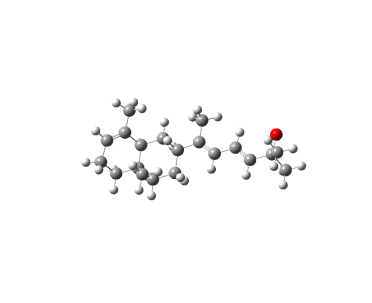

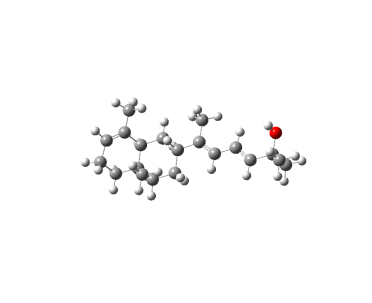

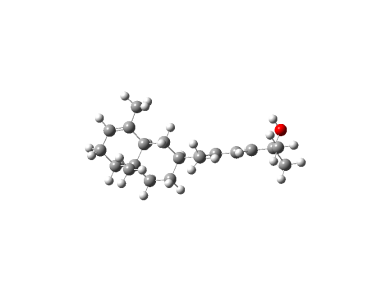


Conf. 7 Conf. 8 Conf. 9

4.45% 4.31% 3.10%


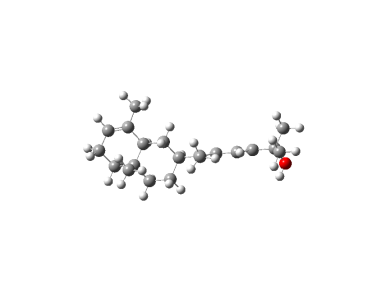

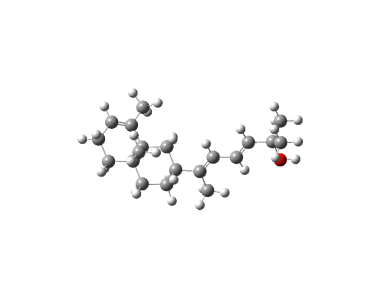

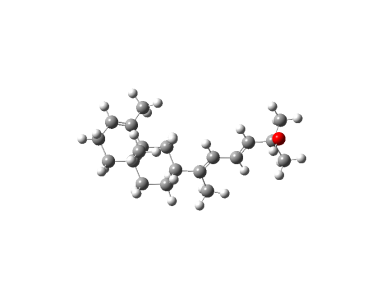


Conf. 10 Conf. 11 Conf. 12

3.06% 2.68% 1.18%


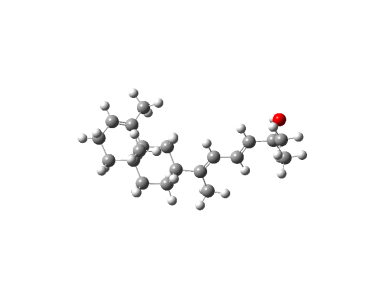

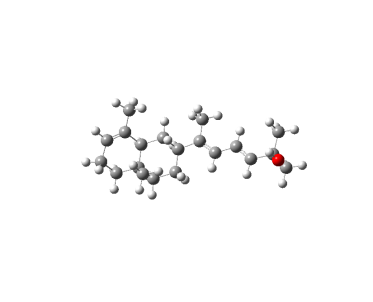

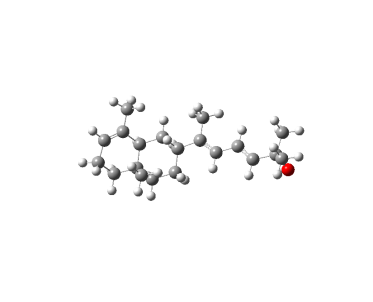


Conf. 13 Conf. 14 Conf. 15

1.17% 0.76% 0.75%


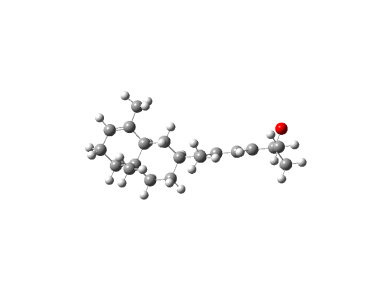

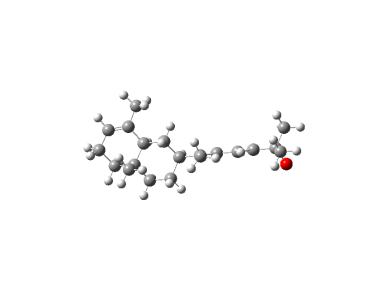

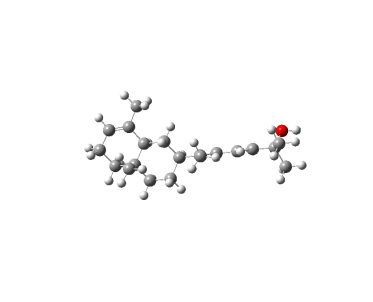


Conf. 16 Conf. 17 Conf. 18

0.74% 0.73% 0.68%


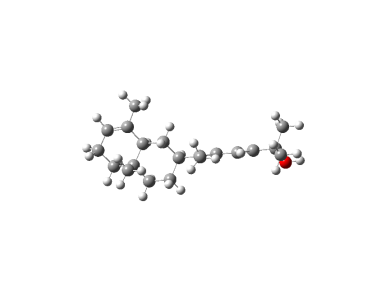

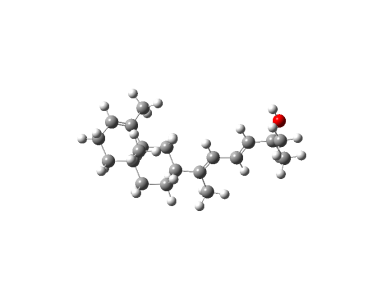


Conf. 19 Conf. 20

0.62% 0.28%

**Figure S1.** Re-optimized conformers above 1% population (OPLS_2005) of (*5S, 10S, 11S*)-**8** calculated at the B3LYP/6-311G(d,p) level with IEFPCM solvent model for acetonitrile.


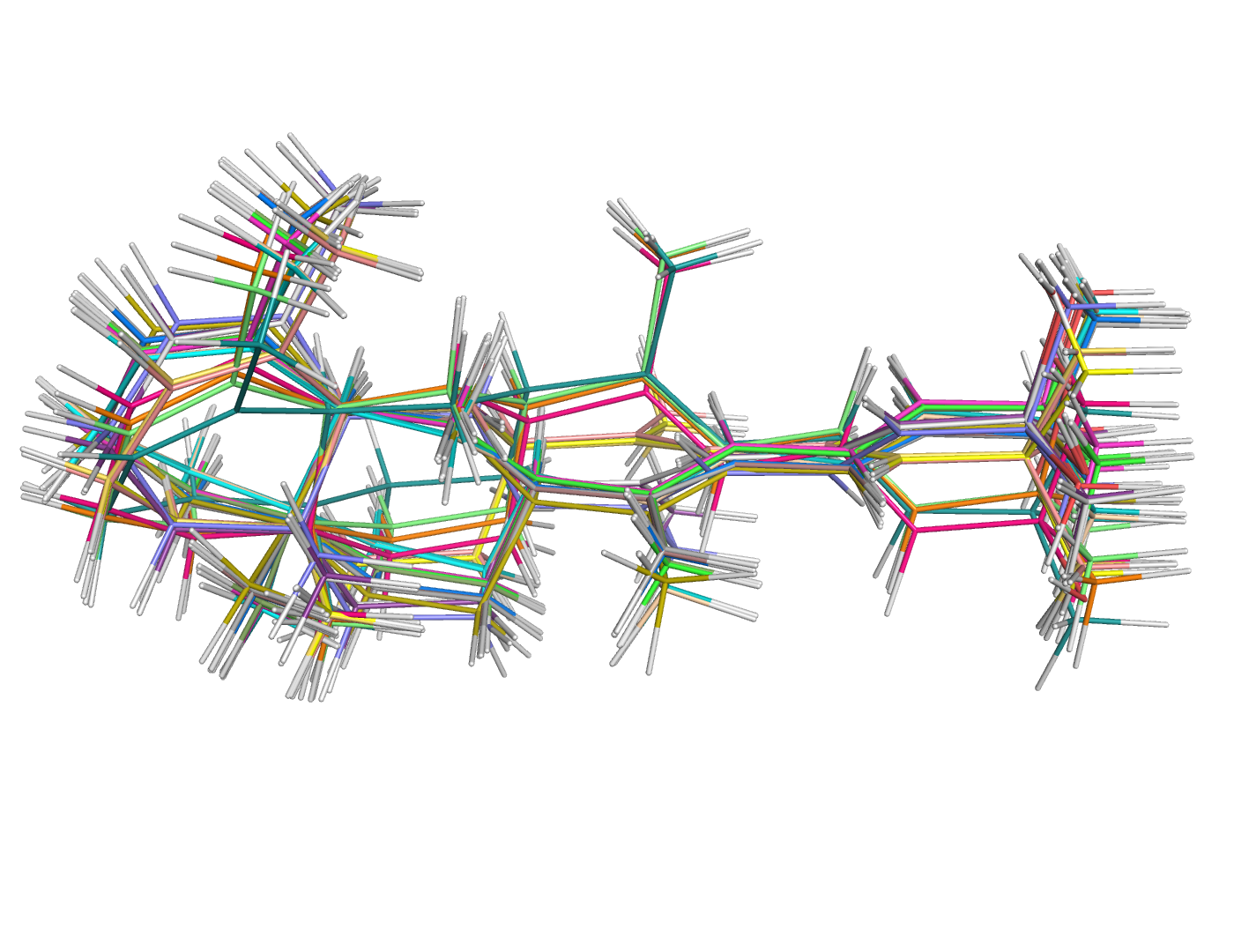


**Figure S2.** Superimposed figure of the re-optimized conformers above 1% population of (5*S*, 10*S*, 11*S*) for **8**.

**Table S1.** Cartesian coordinates for the re-optimized conformers of compound **8** at the B3LYP/6-311G(d,p) level with IEFPCM solvent model for acetonitrile.

| Compound **8**  Conformer 1 | | Standard Orientation  (Ångstroms) | | |
| --- | --- | --- | --- | --- |
| 1 | C | -5.76199700 | 0.06932000 | -0.59467300 |
| 2 | C | -5.13854800 | 1.39583600 | -0.24869900 |
| 3 | C | -3.82456500 | 1.61553500 | -0.11958100 |
| 4 | C | -2.81606000 | 0.49567500 | -0.37152800 |
| 5 | C | -3.44064500 | -0.91679100 | -0.17108600 |
| 6 | C | -4.72489700 | -0.98051300 | -1.02195200 |
| 7 | C | -1.47389300 | 0.63081900 | 0.37500100 |
| 8 | C | -0.46184000 | -0.42584700 | -0.12726000 |
| 9 | C | -1.04571000 | -1.85292900 | -0.00836500 |
| 10 | C | -2.42501200 | -1.96533400 | -0.67592400 |
| 11 | C | -3.29994500 | 2.99502000 | 0.19589900 |
| 12 | C | -3.79478300 | -1.20006000 | 1.30512300 |
| 13 | C | 0.90532100 | -0.30486600 | 0.52666900 |
| 14 | C | 1.99843400 | -0.15337600 | -0.25048300 |
| 15 | C | 0.95778700 | -0.36822900 | 2.03341900 |
| 16 | C | 3.38616200 | -0.04351900 | 0.17410700 |
| 17 | C | 4.41442900 | 0.10481600 | -0.67408700 |
| 18 | C | 5.87322600 | 0.21287200 | -0.27831500 |
| 19 | O | 6.05772100 | -0.02289500 | 1.12855900 |
| 20 | C | 6.70966900 | -0.79720300 | -1.08382100 |
| 21 | C | 6.38090600 | 1.64191200 | -0.51447600 |
| 22 | H | -6.49883400 | 0.20923500 | -1.39479100 |
| 23 | H | -6.34219100 | -0.29233400 | 0.26540700 |
| 24 | H | -5.82616900 | 2.22476100 | -0.09018400 |
| 25 | H | -2.56077800 | 0.56245900 | -1.44186900 |
| 26 | H | -4.45223600 | -0.81301200 | -2.07081300 |
| 27 | H | -5.16061300 | -1.98451700 | -0.96718900 |
| 28 | H | -1.62848000 | 0.53592400 | 1.45431400 |
| 29 | H | -1.04773800 | 1.62355100 | 0.20708700 |
| 30 | H | -0.31978700 | -0.23438700 | -1.19852200 |
| 31 | H | -0.35364400 | -2.56203600 | -0.47415700 |
| 32 | H | -1.11835000 | -2.14182800 | 1.04448000 |
| 33 | H | -2.30722800 | -1.84121700 | -1.76011900 |
| 34 | H | -2.83065600 | -2.97200600 | -0.52068100 |
| 35 | H | -2.60069900 | 3.34216200 | -0.57405200 |
| 36 | H | -4.11710300 | 3.71670600 | 0.25916900 |
| 37 | H | -2.75442300 | 3.01805600 | 1.14511600 |
| 38 | H | -4.30586500 | -2.16524200 | 1.38284100 |
| 39 | H | -2.91287100 | -1.24626700 | 1.94553200 |
| 40 | H | -4.45579800 | -0.43535900 | 1.71715400 |
| 41 | H | 1.84277400 | -0.10954400 | -1.32770700 |
| 42 | H | 1.97464700 | -0.44676900 | 2.41535700 |
| 43 | H | 0.50669800 | 0.52564900 | 2.47775100 |
| 44 | H | 0.39250400 | -1.22414200 | 2.41430800 |
| 45 | H | 3.60704800 | -0.08027800 | 1.23609300 |
| 46 | H | 4.22260900 | 0.15770500 | -1.74478400 |
| 47 | H | 5.80199200 | -0.93544300 | 1.30830600 |
| 48 | H | 7.76297900 | -0.71482900 | -0.80477900 |
| 49 | H | 6.37420900 | -1.81926100 | -0.88366900 |
| 50 | H | 6.61769700 | -0.61761400 | -2.15820300 |
| 51 | H | 7.44184500 | 1.70950000 | -0.25934800 |
| 52 | H | 6.25460300 | 1.93087000 | -1.5600490 |
| 53 | H | 5.82385200 | 2.34383600 | 0.10972500 |

B3LYP/6-311G(d,p) Energy =-856.8324544 a.u.; Population = 18.81%.

| Compound **8**  Conformer 2 | | Standard Orientation  (Ångstroms) | | |
| --- | --- | --- | --- | --- |
| 1 | C | -5.76113200 | 0.07903800 | -0.59641800 |
| 2 | C | -5.13606500 | 1.40158500 | -0.23844900 |
| 3 | C | -3.82186000 | 1.61822200 | -0.10614700 |
| 4 | C | -2.81473400 | 0.49904800 | -0.36645600 |
| 5 | C | -3.44164800 | -0.91419700 | -0.17919900 |
| 6 | C | -4.72516800 | -0.96856900 | -1.03180700 |
| 7 | C | -1.47338000 | 0.62541700 | 0.38312400 |
| 8 | C | -0.46225200 | -0.42792100 | -0.12819500 |
| 9 | C | -1.04817700 | -1.85509100 | -0.02286600 |
| 10 | C | -2.42709600 | -1.95978400 | -0.69240700 |
| 11 | C | -3.29557800 | 2.99427000 | 0.22132100 |
| 12 | C | -3.79764900 | -1.20983900 | 1.29417400 |
| 13 | C | 0.90527800 | -0.31510100 | 0.52674000 |
| 14 | C | 1.99751500 | -0.15061700 | -0.24904200 |
| 15 | C | 0.95911000 | -0.40226200 | 2.03220800 |
| 16 | C | 3.38634200 | -0.05156500 | 0.17472300 |
| 17 | C | 4.41295300 | 0.11274200 | -0.67231500 |
| 18 | C | 5.87271100 | 0.21322700 | -0.27810300 |
| 19 | O | 6.03473600 | 0.23912200 | 1.15078200 |
| 20 | C | 6.63154500 | -1.03655300 | -0.74489200 |
| 21 | C | 6.49377400 | 1.48172900 | -0.88938800 |
| 22 | H | -6.49704200 | 0.22695600 | -1.39595200 |
| 23 | H | -6.34266700 | -0.28923500 | 0.25992600 |
| 24 | H | -5.82258700 | 2.23019800 | -0.07368200 |
| 25 | H | -2.55796400 | 0.57498800 | -1.43583800 |
| 26 | H | -4.45117000 | -0.79235800 | -2.07887300 |
| 27 | H | -5.16234800 | -1.97237400 | -0.98618000 |
| 28 | H | -1.62976000 | 0.52032000 | 1.46122300 |
| 29 | H | -1.04560500 | 1.61909500 | 0.22523700 |
| 30 | H | -0.31965400 | -0.22654900 | -1.19756300 |
| 31 | H | -0.35666600 | -2.56105700 | -0.49422800 |
| 32 | H | -1.12233400 | -2.15331800 | 1.02720300 |
| 33 | H | -2.30850800 | -1.82661800 | -1.77542800 |
| 34 | H | -2.83424600 | -2.96717500 | -0.54602400 |
| 35 | H | -2.59476800 | 3.34665700 | -0.54483500 |
| 36 | H | -4.11170600 | 3.71668200 | 0.28936600 |
| 37 | H | -2.75129400 | 3.00877200 | 1.17142300 |
| 38 | H | -4.30898300 | -2.17553700 | 1.36325400 |
| 39 | H | -2.91657900 | -1.26152900 | 1.93534300 |
| 40 | H | -4.45902400 | -0.44848100 | 1.71180000 |
| 41 | H | 1.84027800 | -0.08797400 | -1.32510600 |
| 42 | H | 1.97740400 | -0.46180000 | 2.41349900 |
| 43 | H | 0.48536600 | 0.47237700 | 2.49091100 |
| 44 | H | 0.41539700 | -1.27829200 | 2.39870600 |
| 45 | H | 3.61093600 | -0.12306900 | 1.23410800 |
| 46 | H | 4.22012000 | 0.17832500 | -1.74215000 |
| 47 | H | 5.63534100 | 1.05439300 | 1.47655500 |
| 48 | H | 7.69017700 | -0.94988000 | -0.48626700 |
| 49 | H | 6.22281200 | -1.92521100 | -0.25919300 |
| 50 | H | 6.54571300 | -1.16129800 | -1.82646300 |
| 51 | H | 7.54979900 | 1.54846600 | -0.61642800 |
| 52 | H | 6.41434300 | 1.47403800 | -1.97955300 |
| 53 | H | 5.98038500 | 2.37484700 | -0.52067900 |

B3LYP/6-311G(d,p) Energy = -856.832431 a.u.; Population = 18.35%.

| Compound **8**  Conformer 3 | | Standard Orientation  (Ångstroms) | | |
| --- | --- | --- | --- | --- |
| 1 | C | -5.72945400 | 0.34883100 | -0.53706600 |
| 2 | C | -5.00622800 | 1.59124600 | -0.08819700 |
| 3 | C | -3.67828800 | 1.70148100 | 0.03940400 |
| 4 | C | -2.75872400 | 0.53589800 | -0.32153400 |
| 5 | C | -3.48629300 | -0.83915100 | -0.23792500 |
| 6 | C | -4.77712800 | -0.72988900 | -1.07410500 |
| 7 | C | -1.40493900 | 0.50462500 | 0.41383500 |
| 8 | C | -0.47861500 | -0.57756900 | -0.19402300 |
| 9 | C | -1.16634300 | -1.96063000 | -0.18642400 |
| 10 | C | -2.55420100 | -1.91189900 | -0.84416800 |
| 11 | C | -3.04842600 | 3.00709700 | 0.45886300 |
| 12 | C | -3.85160800 | -1.22448200 | 1.21227700 |
| 13 | C | 0.90011300 | -0.59174300 | 0.44681800 |
| 14 | C | 1.96996600 | -0.20231100 | -0.27838800 |
| 15 | C | 0.99256600 | -1.05167900 | 1.88126500 |
| 16 | C | 3.36035200 | -0.14447200 | 0.14754300 |
| 17 | C | 4.36834100 | 0.24695200 | -0.64582800 |
| 18 | C | 5.82747500 | 0.31790000 | -0.24221600 |
| 19 | O | 6.00101000 | 0.05865400 | 1.16179800 |
| 20 | C | 6.62499600 | -0.78356400 | -0.95387600 |
| 21 | C | 6.39875300 | 1.70553200 | -0.58335700 |
| 22 | H | -6.46383900 | 0.61131400 | -1.30791600 |
| 23 | H | -6.32311600 | -0.04619100 | 0.29872000 |
| 24 | H | -5.62877200 | 2.45197100 | 0.14967800 |
| 25 | H | -2.50615400 | 0.67796500 | -1.38531800 |
| 26 | H | -4.49931300 | -0.48640600 | -2.10642900 |
| 27 | H | -5.28591400 | -1.69994600 | -1.10598300 |
| 28 | H | -1.55633900 | 0.33081100 | 1.48380800 |
| 29 | H | -0.90502300 | 1.47219000 | 0.32244900 |
| 30 | H | -0.33469400 | -0.30012100 | -1.24614800 |
| 31 | H | -0.53398300 | -2.68149900 | -0.71563600 |
| 32 | H | -1.25501400 | -2.32734800 | 0.84050600 |
| 33 | H | -2.43424900 | -1.70428500 | -1.91525100 |
| 34 | H | -3.03308200 | -2.89537200 | -0.77019000 |
| 35 | H | -2.33469100 | 3.36521200 | -0.29266100 |
| 36 | H | -3.80845800 | 3.77911200 | 0.59637200 |
| 37 | H | -2.49068200 | 2.90991100 | 1.39613600 |
| 38 | H | -4.42877800 | -2.15490700 | 1.21305200 |
| 39 | H | -2.97215100 | -1.38797800 | 1.83662000 |
| 40 | H | -4.45424500 | -0.45421500 | 1.69678700 |
| 41 | H | 1.79026400 | 0.10428500 | -1.30806500 |
| 42 | H | 1.97039700 | -0.85980900 | 2.32061200 |
| 43 | H | 0.24485700 | -0.55392800 | 2.50569500 |
| 44 | H | 0.80066600 | -2.12707800 | 1.96247500 |
| 45 | H | 3.60286200 | -0.44316600 | 1.16238600 |
| 46 | H | 4.16004300 | 0.53933700 | -1.67401500 |
| 47 | H | 5.56790600 | 0.77111200 | 1.64675300 |
| 48 | H | 7.68291800 | -0.71126500 | -0.68808900 |
| 49 | H | 6.25319100 | -1.76565100 | -0.65395200 |
| 50 | H | 6.53045300 | -0.69254800 | -2.03810700 |
| 51 | H | 7.45263700 | 1.75472900 | -0.29858200 |
| 52 | H | 6.31596600 | 1.91465200 | -1.65310500 |
| 53 | H | 5.85436900 | 2.48685100 | -0.04437600 |

B3LYP/6-311G(d,p) Energy = -856.8323956 a.u.; Population = 17.67%.

| Compound **8**  Conformer 4 | | Standard Orientation  (Ångstroms) | | |
| --- | --- | --- | --- | --- |
| 1 | C | -5.76136800 | 0.08404600 | -0.59936300 |
| 2 | C | -5.13451800 | 1.40575200 | -0.24130100 |
| 3 | C | -3.82011000 | 1.62020100 | -0.10757100 |
| 4 | C | -2.81450700 | 0.49927500 | -0.36625300 |
| 5 | C | -3.44392200 | -0.91285800 | -0.17916900 |
| 6 | C | -4.72663900 | -0.96550000 | -1.03306200 |
| 7 | C | -1.47377500 | 0.62377300 | 0.38470600 |
| 8 | C | -0.46356200 | -0.43137400 | -0.12471000 |
| 9 | C | -1.05205300 | -1.85751800 | -0.02013700 |
| 10 | C | -2.43052600 | -1.96024700 | -0.69097400 |
| 11 | C | -3.29188200 | 2.99551900 | 0.21987800 |
| 12 | C | -3.80190000 | -1.20736500 | 1.29392300 |
| 13 | C | 0.90298400 | -0.32023300 | 0.53264200 |
| 14 | C | 1.99720000 | -0.15546700 | -0.24032400 |
| 15 | C | 0.95369700 | -0.40767900 | 2.03835700 |
| 16 | C | 3.38488600 | -0.05489000 | 0.18678900 |
| 17 | C | 4.40874500 | 0.11338600 | -0.66237300 |
| 18 | C | 5.86530400 | 0.21558500 | -0.28084000 |
| 19 | O | 5.95332000 | 0.07315500 | 1.14721700 |
| 20 | C | 6.66377500 | -0.91354800 | -0.95860400 |
| 21 | C | 6.42018700 | 1.59184100 | -0.69305800 |
| 22 | H | -6.49606100 | 0.23280300 | -1.39985500 |
| 23 | H | -6.34452700 | -0.28281700 | 0.25649200 |
| 24 | H | -5.81984400 | 2.23558600 | -0.07771500 |
| 25 | H | -2.55645200 | 0.57435300 | -1.43538800 |
| 26 | H | -4.45122900 | -0.79029700 | -2.07993500 |
| 27 | H | -5.16556800 | -1.96854500 | -0.98738800 |
| 28 | H | -1.63141100 | 0.51962600 | 1.46273000 |
| 29 | H | -1.04412100 | 1.61663700 | 0.22677200 |
| 30 | H | -0.31886000 | -0.23038300 | -1.19389700 |
| 31 | H | -0.36118000 | -2.56459200 | -0.49081600 |
| 32 | H | -1.12767800 | -2.15575100 | 1.02984500 |
| 33 | H | -2.31071300 | -1.82759300 | -1.77393600 |
| 34 | H | -2.83950900 | -2.96693700 | -0.54472600 |
| 35 | H | -2.58951100 | 3.34630600 | -0.54556800 |
| 36 | H | -4.10685900 | 3.71937000 | 0.28655500 |
| 37 | H | -2.74876900 | 3.00951500 | 1.17065200 |
| 38 | H | -4.31468900 | -2.17230500 | 1.36292000 |
| 39 | H | -2.92155600 | -1.26001700 | 1.93602000 |
| 40 | H | -4.46262100 | -0.44486600 | 1.71053700 |
| 41 | H | 1.84206600 | -0.09262100 | -1.31687100 |
| 42 | H | 1.97088800 | -0.47943400 | 2.42071100 |
| 43 | H | 0.49035300 | 0.47288800 | 2.49653000 |
| 44 | H | 0.39900200 | -1.27687600 | 2.40465200 |
| 45 | H | 3.61259000 | -0.12165800 | 1.24451600 |
| 46 | H | 4.21626900 | 0.18649000 | -1.73118200 |
| 47 | H | 6.88399100 | 0.14087800 | 1.38760800 |
| 48 | H | 7.72466500 | -0.83601000 | -0.69979600 |
| 49 | H | 6.29353600 | -1.88515000 | -0.62471000 |
| 50 | H | 6.57713500 | -0.86214000 | -2.04714400 |
| 51 | H | 7.48262000 | 1.66491300 | -0.43867900 |
| 52 | H | 6.31886100 | 1.75396200 | -1.76935500 |
| 53 | H | 5.88052300 | 2.38322800 | -0.16864400 |

B3LYP/6-311G(d,p) Energy = -856.8315454 a.u.; Population = 7.18%.

| Compound **8**  Conformer 5 | | Standard Orientation  (Ångstroms) | | |
| --- | --- | --- | --- | --- |
| 1 | C | -5.46362800 | 0.75576200 | 0.59358000 |
| 2 | C | -4.65966700 | 1.81390100 | -0.11518500 |
| 3 | C | -3.43180000 | 1.63611300 | -0.61760300 |
| 4 | C | -2.74774700 | 0.27175200 | -0.54451600 |
| 5 | C | -3.29725200 | -0.60116500 | 0.62300500 |
| 6 | C | -4.83283300 | -0.64065700 | 0.48680300 |
| 7 | C | -1.20609000 | 0.30004000 | -0.55205100 |
| 8 | C | -0.64246200 | -1.12464000 | -0.71936000 |
| 9 | C | -1.18473200 | -2.05835700 | 0.39070900 |
| 10 | C | -2.71858000 | -2.02547400 | 0.47137500 |
| 11 | C | -2.72563400 | 2.75530900 | -1.34288600 |
| 12 | C | -2.92458300 | -0.03139700 | 2.00893300 |
| 13 | C | 0.87304000 | -1.22103400 | -0.83273600 |
| 14 | C | 1.69768700 | -0.34235200 | -0.22727400 |
| 15 | C | 1.35500100 | -2.38292900 | -1.66593300 |
| 16 | C | 3.15344700 | -0.33950800 | -0.24659400 |
| 17 | C | 3.90497300 | 0.57253300 | 0.38766000 |
| 18 | C | 5.41993200 | 0.61542900 | 0.38901300 |
| 19 | O | 5.97599800 | -0.34681900 | -0.52443400 |
| 20 | C | 5.90509900 | 2.02536200 | 0.00826000 |
| 21 | C | 5.95944100 | 0.21510300 | 1.76906600 |
| 22 | H | -6.48115400 | 0.73442800 | 0.18519200 |
| 23 | H | -5.58675700 | 1.04132400 | 1.64739400 |
| 24 | H | -5.12819400 | 2.79190400 | -0.21005600 |
| 25 | H | -3.03316400 | -0.25128400 | -1.47214300 |
| 26 | H | -5.08128800 | -1.07610600 | -0.48834700 |
| 27 | H | -5.25939400 | -1.30583100 | 1.24605000 |
| 28 | H | -0.83595700 | 0.76253600 | 0.36781700 |
| 29 | H | -0.84220900 | 0.91604600 | -1.37803800 |
| 30 | H | -1.04734500 | -1.50365400 | -1.66944400 |
| 31 | H | -0.85365000 | -3.08575500 | 0.21016100 |
| 32 | H | -0.74473700 | -1.75826900 | 1.34759100 |
| 33 | H | -3.13120400 | -2.47456900 | -0.44125000 |
| 34 | H | -3.05887300 | -2.64898600 | 1.30650300 |
| 35 | H | -2.45768600 | 2.46238800 | -2.36500300 |
| 36 | H | -3.36086700 | 3.64168800 | -1.40174700 |
| 37 | H | -1.79242900 | 3.03998200 | -0.84577800 |
| 38 | H | -3.40895300 | -0.62205000 | 2.79329100 |
| 39 | H | -1.85044900 | -0.05974500 | 2.19695100 |
| 40 | H | -3.24664400 | 1.00541100 | 2.12096300 |
| 41 | H | 1.25198200 | 0.46829400 | 0.34348900 |
| 42 | H | 2.43477700 | -2.52115800 | -1.63579800 |
| 43 | H | 0.88896100 | -3.31764200 | -1.33669500 |
| 44 | H | 1.06138000 | -2.24371000 | -2.71327800 |
| 45 | H | 3.66619700 | -1.11809800 | -0.80221400 |
| 46 | H | 3.42111400 | 1.36055800 | 0.96290200 |
| 47 | H | 5.70416000 | -0.09498700 | -1.41503700 |
| 48 | H | 6.99731500 | 2.05756300 | 0.01580400 |
| 49 | H | 5.55510700 | 2.29376100 | -0.99302800 |
| 50 | H | 5.52863800 | 2.77554200 | 0.70866400 |
| 51 | H | 7.05094500 | 0.27616900 | 1.77684000 |
| 52 | H | 5.56766600 | 0.87574800 | 2.54544600 |
| 53 | H | 5.66305900 | -0.80969700 | 2.00267000 |

B3LYP/6-311G(d,p) Energy = -856.8314897 a.u.; Population = 6.76% .

| Compound **8**  Conformer 6 | | Standard Orientation  (Ångstroms) | | |
| --- | --- | --- | --- | --- |
| 1 | C | -5.46247800 | 0.75673800 | 0.59603800 |
| 2 | C | -4.66035200 | 1.81279400 | -0.11793000 |
| 3 | C | -3.43338300 | 1.63378600 | -0.62206900 |
| 4 | C | -2.74849300 | 0.27002700 | -0.54555100 |
| 5 | C | -3.29560800 | -0.59939600 | 0.62566200 |
| 6 | C | -4.83142200 | -0.63979900 | 0.49248700 |
| 7 | C | -1.20686800 | 0.29904900 | -0.55577600 |
| 8 | C | -0.64285800 | -1.12603000 | -0.71904400 |
| 9 | C | -1.18304500 | -2.05667400 | 0.39452300 |
| 10 | C | -2.71680700 | -2.02405200 | 0.47746300 |
| 11 | C | -2.72916800 | 2.75056700 | -1.35298600 |
| 12 | C | -2.92055600 | -0.02530300 | 2.00916300 |
| 13 | C | 0.87262900 | -1.22181600 | -0.83413700 |
| 14 | C | 1.69752300 | -0.34525600 | -0.22612000 |
| 15 | C | 1.35399800 | -2.38048000 | -1.67210400 |
| 16 | C | 3.15328100 | -0.33933800 | -0.25060000 |
| 17 | C | 3.90475500 | 0.57029200 | 0.38695100 |
| 18 | C | 5.41944300 | 0.61781200 | 0.38666000 |
| 19 | O | 5.98443800 | -0.52009800 | -0.28770100 |
| 20 | C | 5.90837800 | 1.83681300 | -0.40733100 |
| 21 | C | 5.94241300 | 0.67027400 | 1.83296300 |
| 22 | H | -6.48083800 | 0.73370700 | 0.18983900 |
| 23 | H | -5.58344800 | 1.04570100 | 1.64917700 |
| 24 | H | -5.12958200 | 2.79020100 | -0.21547300 |
| 25 | H | -3.03515300 | -0.25614000 | -1.47103200 |
| 26 | H | -5.08157700 | -1.07841800 | -0.48081100 |
| 27 | H | -5.25634700 | -1.30271100 | 1.25464000 |
| 28 | H | -0.83538500 | 0.76511400 | 0.36171600 |
| 29 | H | -0.84454200 | 0.91206300 | -1.38465400 |
| 30 | H | -1.04881800 | -1.50808600 | -1.66738700 |
| 31 | H | -0.85172400 | -3.08444800 | 0.21622400 |
| 32 | H | -0.74172500 | -1.75375900 | 1.34990800 |
| 33 | H | -3.13071900 | -2.47599200 | -0.43316700 |
| 34 | H | -3.05568200 | -2.64509900 | 1.31501300 |
| 35 | H | -2.46301900 | 2.45379000 | -2.37444900 |
| 36 | H | -3.36498200 | 3.63638500 | -1.41405100 |
| 37 | H | -1.79517800 | 3.03757200 | -0.85873500 |
| 38 | H | -3.40316300 | -0.61383300 | 2.79620700 |
| 39 | H | -1.84605600 | -0.05258600 | 2.19523600 |
| 40 | H | -3.24281700 | 1.01170500 | 2.11878100 |
| 41 | H | 1.25210200 | 0.46192000 | 0.34980700 |
| 42 | H | 2.43302200 | -2.52379300 | -1.63691400 |
| 43 | H | 0.88163100 | -3.31489600 | -1.35103900 |
| 44 | H | 1.06700100 | -2.23349300 | -2.72021000 |
| 45 | H | 3.66446600 | -1.10630900 | -0.82354900 |
| 46 | H | 3.42032400 | 1.35949000 | 0.96025300 |
| 47 | H | 5.75664100 | -1.30608800 | 0.22289000 |
| 48 | H | 7.00020600 | 1.88604700 | -0.38333400 |
| 49 | H | 5.58294400 | 1.76029600 | -1.44701300 |
| 50 | H | 5.50813200 | 2.76098000 | 0.01512800 |
| 51 | H | 7.03441100 | 0.71223600 | 1.83271800 |
| 52 | H | 5.55916600 | 1.54823000 | 2.35950500 |
| 53 | H | 5.62692700 | -0.21905800 | 2.38681300 |

B3LYP/6-311G(d,p) Energy = -856.8314845 a.u.; Population = 6.73%.

| Compound **8**  Conformer 7 | | Standard Orientation  (Ångstroms) | | |
| --- | --- | --- | --- | --- |
| 1 | C | -5.63284500 | -0.69503200 | 0.48382000 |
| 2 | C | -5.22819100 | 0.73151500 | 0.74778100 |
| 3 | C | -4.03817300 | 1.26096800 | 0.43935900 |
| 4 | C | -2.97631900 | 0.42832600 | -0.27735600 |
| 5 | C | -3.16315000 | -1.09845000 | -0.03719800 |
| 6 | C | -4.62147700 | -1.44797900 | -0.39431700 |
| 7 | C | -1.51794600 | 0.86858500 | -0.04537300 |
| 8 | C | -0.54985200 | 0.09874500 | -0.98269100 |
| 9 | C | -0.72648000 | -1.41846400 | -0.81108900 |
| 10 | C | -2.19293400 | -1.84764600 | -0.97679500 |
| 11 | C | -3.74552500 | 2.71540300 | 0.71603400 |
| 12 | C | -2.88906000 | -1.50258400 | 1.42770400 |
| 13 | C | 0.87620800 | 0.61793600 | -0.85949800 |
| 14 | C | 1.89048700 | -0.14459600 | -0.40184900 |
| 15 | C | 1.06285400 | 2.05611000 | -1.28942200 |
| 16 | C | 3.28095500 | 0.25614100 | -0.23829900 |
| 17 | C | 4.23888200 | -0.55213400 | 0.23886500 |
| 18 | C | 5.69578500 | -0.17657200 | 0.42070800 |
| 19 | O | 5.98025000 | 1.12051200 | -0.13181100 |
| 20 | C | 6.02883800 | -0.05676200 | 1.91418500 |
| 21 | C | 6.59547600 | -1.22975300 | -0.24999900 |
| 22 | H | -6.62109500 | -0.71299000 | 0.00875200 |
| 23 | H | -5.76937700 | -1.21451300 | 1.44220700 |
| 24 | H | -5.97218800 | 1.36118300 | 1.23247300 |
| 25 | H | -3.15368000 | 0.58589000 | -1.35398400 |
| 26 | H | -4.79099900 | -1.18841100 | -1.44597300 |
| 27 | H | -4.77923900 | -2.52897500 | -0.30810300 |
| 28 | H | -1.21889800 | 0.71322900 | 0.99740500 |
| 29 | H | -1.42650300 | 1.93880500 | -0.23815500 |
| 30 | H | -0.87064800 | 0.34214600 | -2.00823600 |
| 31 | H | -0.10808700 | -1.94713400 | -1.54370600 |
| 32 | H | -0.36512600 | -1.72710300 | 0.17471200 |
| 33 | H | -2.50336000 | -1.66812800 | -2.01416900 |
| 34 | H | -2.28166700 | -2.92727200 | -0.80837400 |
| 35 | H | -3.47056800 | 3.24950900 | -0.20131400 |
| 36 | H | -4.61772900 | 3.21230100 | 1.14599600 |
| 37 | H | -2.90847100 | 2.83895900 | 1.41115600 |
| 38 | H | -3.50613200 | -0.93554300 | 2.12683000 |
| 39 | H | -3.11342900 | -2.56540500 | 1.56503500 |
| 40 | H | -1.84847500 | -1.35038200 | 1.71698900 |
| 41 | H | 1.67810300 | -1.16995700 | -0.11401900 |
| 42 | H | 2.09602000 | 2.28762100 | -1.54712900 |
| 43 | H | 0.44166000 | 2.27929900 | -2.16224200 |
| 44 | H | 0.75985000 | 2.75324200 | -0.50027800 |
| 45 | H | 3.56277600 | 1.26873200 | -0.50844700 |
| 46 | H | 3.98518400 | -1.56872600 | 0.53595000 |
| 47 | H | 5.83818100 | 1.07048800 | -1.08456500 |
| 48 | H | 7.08766500 | 0.18273900 | 2.04331600 |
| 49 | H | 5.43114000 | 0.73681000 | 2.36759600 |
| 50 | H | 5.81685000 | -0.99277000 | 2.43544900 |
| 51 | H | 7.64611800 | -0.96017500 | -0.11730200 |
| 52 | H | 6.43196800 | -2.22100600 | 0.18084200 |
| 53 | H | 6.38296800 | -1.28910900 | -1.32164300 |

B3LYP/6-311G(d,p) Energy = -856.8310938 a.u.; Population = 4.45% .

| Compound **8**  Conformer 8 | | Standard Orientation  (Ångstroms) | | |
| --- | --- | --- | --- | --- |
| 1 | C | -5.62988900 | -0.69983300 | 0.48720500 |
| 2 | C | -5.22618800 | 0.72632900 | 0.75477500 |
| 3 | C | -4.03763000 | 1.25813600 | 0.44477700 |
| 4 | C | -2.97657400 | 0.42904100 | -0.27719800 |
| 5 | C | -3.16152200 | -1.09877300 | -0.04290400 |
| 6 | C | -4.62056600 | -1.44812700 | -0.39722100 |
| 7 | C | -1.51825300 | 0.86952300 | -0.04570400 |
| 8 | C | -0.55019000 | 0.10484300 | -0.98741000 |
| 9 | C | -0.72654900 | -1.41337700 | -0.82428400 |
| 10 | C | -2.19317300 | -1.84256600 | -0.98865900 |
| 11 | C | -3.74613800 | 2.71221400 | 0.72469500 |
| 12 | C | -2.88293400 | -1.50877300 | 1.41952200 |
| 13 | C | 0.87573800 | 0.62345700 | -0.85918800 |
| 14 | C | 1.89134800 | -0.14453300 | -0.41372700 |
| 15 | C | 1.06098800 | 2.06758300 | -1.26950700 |
| 16 | C | 3.28068900 | 0.25684200 | -0.24165600 |
| 17 | C | 4.24136100 | -0.55939400 | 0.21601100 |
| 18 | C | 5.69492400 | -0.18063800 | 0.41781700 |
| 19 | O | 5.90757900 | 1.22699000 | 0.21229300 |
| 20 | C | 6.14423000 | -0.56576800 | 1.83828500 |
| 21 | C | 6.57324900 | -0.87617400 | -0.63120600 |
| 22 | H | -6.61977000 | -0.71736900 | 0.01555100 |
| 23 | H | -5.76230400 | -1.22301300 | 1.44415200 |
| 24 | H | -5.96965800 | 1.35365600 | 1.24329900 |
| 25 | H | -3.15595800 | 0.59088300 | -1.35286400 |
| 26 | H | -4.79353800 | -1.18414700 | -1.44720900 |
| 27 | H | -4.77711500 | -2.52962200 | -0.31515400 |
| 28 | H | -1.21773800 | 0.71027000 | 0.99608200 |
| 29 | H | -1.42831500 | 1.94059500 | -0.23415100 |
| 30 | H | -0.87050700 | 0.35440200 | -2.01165300 |
| 31 | H | -0.10981700 | -1.93785800 | -1.56128400 |
| 32 | H | -0.36269200 | -1.72738200 | 0.15890300 |
| 33 | H | -2.50634000 | -1.65797000 | -2.02431400 |
| 34 | H | -2.28064900 | -2.92310800 | -0.82553900 |
| 35 | H | -3.47388800 | 3.24901700 | -0.19188600 |
| 36 | H | -4.61801100 | 3.20690700 | 1.15786300 |
| 37 | H | -2.90771000 | 2.83504200 | 1.41829100 |
| 38 | H | -3.10704900 | -2.57208100 | 1.55348000 |
| 39 | H | -1.84147100 | -1.35785900 | 1.70627700 |
| 40 | H | -3.49776300 | -0.94432200 | 2.12271700 |
| 41 | H | 1.68071900 | -1.17447500 | -0.14149800 |
| 42 | H | 2.09404600 | 2.30362200 | -1.52318200 |
| 43 | H | 0.44089300 | 2.30128000 | -2.14038600 |
| 44 | H | 0.75554700 | 2.75390900 | -0.47187300 |
| 45 | H | 3.56017000 | 1.27422700 | -0.49551700 |
| 46 | H | 3.99363200 | -1.58810300 | 0.47382100 |
| 47 | H | 5.41594400 | 1.69900300 | 0.89493400 |
| 48 | H | 7.19507800 | -0.30186300 | 1.98034400 |
| 49 | H | 5.54697800 | -0.03539000 | 2.58600500 |
| 50 | H | 6.02643700 | -1.63855000 | 2.01219000 |
| 51 | H | 7.62440700 | -0.62661000 | -0.46389000 |
| 52 | H | 6.45914800 | -1.96085400 | -0.57500800 |
| 53 | H | 6.28914100 | -0.54823000 | -1.63339600 |

B3LYP/6-311G(d,p) Energy = -856.8310644 a.u.; Population = 4.31% .

| Compound **8**  Conformer 9 | | Standard Orientation  (Ångstroms) | | |
| --- | --- | --- | --- | --- |
| 1 | C | -5.75695400 | 0.08230400 | -0.61593200 |
| 2 | C | -5.12981700 | 1.40537900 | -0.26364700 |
| 3 | C | -3.81587400 | 1.61847900 | -0.12344300 |
| 4 | C | -2.81114400 | 0.49377400 | -0.36862800 |
| 5 | C | -3.44397700 | -0.91573600 | -0.17289400 |
| 6 | C | -4.72178600 | -0.97327100 | -1.03384700 |
| 7 | C | -1.47362700 | 0.62229000 | 0.38729100 |
| 8 | C | -0.46365100 | -0.43906700 | -0.10954000 |
| 9 | C | -1.05446000 | -1.86334700 | 0.00685300 |
| 10 | C | -2.42976300 | -1.96950800 | -0.66993700 |
| 11 | C | -3.28708200 | 2.99493700 | 0.19807500 |
| 12 | C | -3.81089200 | -1.19691400 | 1.30054500 |
| 13 | C | 0.90050700 | -0.32286900 | 0.55130300 |
| 14 | C | 1.99649800 | -0.16749700 | -0.22130400 |
| 15 | C | 0.94473900 | -0.39250400 | 2.05791400 |
| 16 | C | 3.38287100 | -0.05756700 | 0.20770400 |
| 17 | C | 4.40813700 | 0.09539400 | -0.64546300 |
| 18 | C | 5.87728200 | 0.22602500 | -0.31089100 |
| 19 | O | 6.36883900 | 1.44433400 | -0.93001100 |
| 20 | C | 6.17647900 | 0.26866900 | 1.19095300 |
| 21 | C | 6.67066400 | -0.89548400 | -0.99180700 |
| 22 | H | -6.48544500 | 0.22639000 | -1.42291600 |
| 23 | H | -6.34741600 | -0.27611600 | 0.23851400 |
| 24 | H | -5.81461700 | 2.23750100 | -0.10987200 |
| 25 | H | -2.54799200 | 0.55955700 | -1.43707400 |
| 26 | H | -4.43969600 | -0.80804500 | -2.08054600 |
| 27 | H | -5.16315900 | -1.97490800 | -0.98188100 |
| 28 | H | -1.63568700 | 0.52700500 | 1.46545200 |
| 29 | H | -1.04159000 | 1.61304600 | 0.22293900 |
| 30 | H | -0.31525500 | -0.24842800 | -1.17998100 |
| 31 | H | -0.36277300 | -2.57626400 | -0.45361100 |
| 32 | H | -1.13580400 | -2.15082700 | 1.05939900 |
| 33 | H | -2.30405900 | -1.84696300 | -1.75338700 |
| 34 | H | -2.84116400 | -2.97410700 | -0.51664400 |
| 35 | H | -2.58000300 | 3.33979000 | -0.56573000 |
| 36 | H | -4.10116100 | 3.72054000 | 0.25580100 |
| 37 | H | -2.74913800 | 3.01403100 | 1.15171300 |
| 38 | H | -4.32596300 | -2.16021700 | 1.37485500 |
| 39 | H | -2.93423700 | -1.24583100 | 1.94800500 |
| 40 | H | -4.47244000 | -0.42957300 | 1.70676500 |
| 41 | H | 1.84584300 | -0.11863000 | -1.29883900 |
| 42 | H | 1.95809500 | -0.47881300 | 2.44755000 |
| 43 | H | 0.49546800 | 0.50215300 | 2.50249000 |
| 44 | H | 0.37232600 | -1.24659900 | 2.43176400 |
| 45 | H | 3.58776500 | -0.10552900 | 1.27199400 |
| 46 | H | 4.20171100 | 0.14778100 | -1.71402300 |
| 47 | H | 5.84553700 | 2.17298700 | -0.57604800 |
| 48 | H | 7.24831200 | 0.41536100 | 1.33899900 |
| 49 | H | 5.64895300 | 1.09671200 | 1.67230100 |
| 50 | H | 5.88351100 | -0.65908000 | 1.68862100 |
| 51 | H | 7.74177300 | -0.73741100 | -0.84037400 |
| 52 | H | 6.39665000 | -1.86885000 | -0.57983300 |
| 53 | H | 6.47204900 | -0.90225700 | -2.06615000 |

B3LYP/6-311G(d,p) Energy = -856.8307548 a.u.; Population = 3.10% .

| Compound **8**  Conformer 10 | | Standard Orientation  (Ångstroms) | | |
| --- | --- | --- | --- | --- |
| 1 | C | -5.76516400 | 0.05607700 | -0.59602700 |
| 2 | C | -5.15245700 | 1.38262200 | -0.23160300 |
| 3 | C | -3.84029900 | 1.61098800 | -0.09857200 |
| 4 | C | -2.82264900 | 0.50257800 | -0.36413700 |
| 5 | C | -3.43664800 | -0.91741900 | -0.18478500 |
| 6 | C | -4.71946000 | -0.97890800 | -1.03795800 |
| 7 | C | -1.48351700 | 0.63743300 | 0.38780300 |
| 8 | C | -0.46209200 | -0.40451700 | -0.12599500 |
| 9 | C | -1.03509400 | -1.83806700 | -0.03210300 |
| 10 | C | -2.41246200 | -1.95057600 | -0.70348500 |
| 11 | C | -3.32685400 | 2.99032500 | 0.23522600 |
| 12 | C | -3.79000400 | -1.22454300 | 1.28687000 |
| 13 | C | 0.90017200 | -0.28675800 | 0.53828400 |
| 14 | C | 1.99955000 | -0.14016300 | -0.23111300 |
| 15 | C | 0.93890100 | -0.34829000 | 2.04551500 |
| 16 | C | 3.38482000 | -0.03916900 | 0.20347100 |
| 17 | C | 4.41543700 | 0.10354300 | -0.64496100 |
| 18 | C | 5.88609600 | 0.19879500 | -0.30448500 |
| 19 | O | 6.59116700 | -0.79374100 | -1.09593000 |
| 20 | C | 6.45081400 | 1.54121900 | -0.78402000 |
| 21 | C | 6.20117100 | -0.02453600 | 1.17807900 |
| 22 | H | -6.50329200 | 0.20114800 | -1.39401000 |
| 23 | H | -6.34205400 | -0.32256100 | 0.25892400 |
| 24 | H | -5.84658500 | 2.20402200 | -0.06273000 |
| 25 | H | -2.56550100 | 0.58668800 | -1.43280000 |
| 26 | H | -4.44715700 | -0.79358800 | -2.08387900 |
| 27 | H | -5.14703400 | -1.98709100 | -0.99851600 |
| 28 | H | -1.64053100 | 0.52656000 | 1.46520800 |
| 29 | H | -1.06437600 | 1.63556100 | 0.23482600 |
| 30 | H | -0.31536200 | -0.19575600 | -1.19326900 |
| 31 | H | -0.33664200 | -2.53326400 | -0.50903300 |
| 32 | H | -1.10599300 | -2.14570600 | 1.01557400 |
| 33 | H | -2.29396600 | -1.80962600 | -1.78549900 |
| 34 | H | -2.81055200 | -2.96250100 | -0.56376600 |
| 35 | H | -2.62987100 | 3.35300200 | -0.52961600 |
| 36 | H | -4.14969500 | 3.70468500 | 0.30718300 |
| 37 | H | -2.78221200 | 3.00541000 | 1.18510700 |
| 38 | H | -4.29382400 | -2.19454900 | 1.35035900 |
| 39 | H | -2.90843000 | -1.27328000 | 1.92756000 |
| 40 | H | -4.45709300 | -0.47084200 | 1.70922900 |
| 41 | H | 1.85326500 | -0.09486400 | -1.30942900 |
| 42 | H | 1.94773900 | -0.46966900 | 2.43786500 |
| 43 | H | 0.52279400 | 0.56557900 | 2.48349800 |
| 44 | H | 0.33438500 | -1.17785700 | 2.42373900 |
| 45 | H | 3.58392500 | -0.08175600 | 1.26911500 |
| 46 | H | 4.21597800 | 0.14892100 | -1.71515100 |
| 47 | H | 6.21879200 | -1.65415000 | -0.86968100 |
| 48 | H | 7.53450600 | 1.56023100 | -0.64099900 |
| 49 | H | 6.24095000 | 1.68346700 | -1.84671200 |
| 50 | H | 6.00744400 | 2.36869000 | -0.22649600 |
| 51 | H | 7.28301400 | 0.00514400 | 1.32292200 |
| 52 | H | 5.74816500 | 0.74424800 | 1.80883900 |
| 53 | H | 5.83829500 | -1.00018800 | 1.51305500 |

B3LYP/6-311G(d,p) Energy = -856.8307407 a.u.; Population = 3.06% .

| Compound **8**  Conformer 11 | | Standard Orientation  (Ångstroms) | | |
| --- | --- | --- | --- | --- |
| 1 | C | -5.46250000 | 0.77901900 | 0.57076000 |
| 2 | C | -4.64323700 | 1.83364700 | -0.12564700 |
| 3 | C | -3.41250400 | 1.64791300 | -0.61811000 |
| 4 | C | -2.74113700 | 0.27720700 | -0.54592100 |
| 5 | C | -3.30747100 | -0.59556300 | 0.61371900 |
| 6 | C | -4.84207000 | -0.62216300 | 0.46447200 |
| 7 | C | -1.19923500 | 0.29198600 | -0.54139000 |
| 8 | C | -0.64564300 | -1.13645500 | -0.70963100 |
| 9 | C | -1.20481400 | -2.06849300 | 0.39365200 |
| 10 | C | -2.73882000 | -2.02378400 | 0.46166100 |
| 11 | C | -2.69038500 | 2.76492300 | -1.33090500 |
| 12 | C | -2.94188700 | -0.03384600 | 2.00480100 |
| 13 | C | 0.86984600 | -1.24614900 | -0.81181200 |
| 14 | C | 1.69859000 | -0.35651100 | -0.22831500 |
| 15 | C | 1.34815600 | -2.43185900 | -1.61348900 |
| 16 | C | 3.15456900 | -0.36457000 | -0.24011100 |
| 17 | C | 3.90302600 | 0.57338800 | 0.35799300 |
| 18 | C | 5.41130800 | 0.61993400 | 0.37731300 |
| 19 | O | 5.89218600 | -0.52513400 | -0.34735700 |
| 20 | C | 5.90415900 | 1.90617400 | -0.31176200 |
| 21 | C | 5.91895800 | 0.55724900 | 1.83021200 |
| 22 | H | -6.47616300 | 0.76761500 | 0.15247700 |
| 23 | H | -5.59360900 | 1.06134700 | 1.62447000 |
| 24 | H | -5.10250500 | 2.81613400 | -0.21947400 |
| 25 | H | -3.02398900 | -0.23940900 | -1.47786900 |
| 26 | H | -5.08582500 | -1.05237300 | -0.51417200 |
| 27 | H | -5.28038900 | -1.28633400 | 1.21787200 |
| 28 | H | -0.83251700 | 0.74733500 | 0.38348000 |
| 29 | H | -0.82377600 | 0.90860100 | -1.36170100 |
| 30 | H | -1.04647400 | -1.50967900 | -1.66397300 |
| 31 | H | -0.88108800 | -3.09824700 | 0.21398500 |
| 32 | H | -0.77051100 | -1.77405900 | 1.35492600 |
| 33 | H | -3.14728800 | -2.46628500 | -0.45606400 |
| 34 | H | -3.09104600 | -2.64765900 | 1.29156300 |
| 35 | H | -2.41582400 | 2.47546500 | -2.35224300 |
| 36 | H | -3.31753000 | 3.65701500 | -1.39034200 |
| 37 | H | -1.75922500 | 3.03897300 | -0.82403400 |
| 38 | H | -3.43714000 | -0.62368800 | 2.78295800 |
| 39 | H | -1.86955900 | -0.07115400 | 2.20127800 |
| 40 | H | -3.25696400 | 1.00496100 | 2.11803400 |
| 41 | H | 1.25618900 | 0.47426800 | 0.31559300 |
| 42 | H | 2.42648600 | -2.57795300 | -1.57086400 |
| 43 | H | 0.87164200 | -3.35522900 | -1.26809000 |
| 44 | H | 1.06542900 | -2.31387000 | -2.66651300 |
| 45 | H | 3.66983100 | -1.16505800 | -0.75849100 |
| 46 | H | 3.42000700 | 1.39341300 | 0.88628500 |
| 47 | H | 6.85491600 | -0.48641400 | -0.34326800 |
| 48 | H | 6.99768800 | 1.95565000 | -0.28666800 |
| 49 | H | 5.57823800 | 1.91932900 | -1.35395500 |
| 50 | H | 5.51446200 | 2.79717000 | 0.18736900 |
| 51 | H | 7.01217700 | 0.61120900 | 1.85384500 |
| 52 | H | 5.52750700 | 1.38807300 | 2.42314600 |
| 53 | H | 5.60591200 | -0.38035800 | 2.29435400 |

B3LYP/6-311G(d,p) Energy = -856.8306178 a.u.; Population = 2.68% .

| Compound **8**  Conformer 12 | | Standard Orientation  (Ångstroms) | | |
| --- | --- | --- | --- | --- |
| 1 | C | -5.45342900 | 0.79219500 | 0.57061500 |
| 2 | C | -4.63791700 | 1.83153500 | -0.15256600 |
| 3 | C | -3.41081600 | 1.63446200 | -0.64951600 |
| 4 | C | -2.74039900 | 0.26475600 | -0.55416600 |
| 5 | C | -3.30061000 | -0.58487800 | 0.62535000 |
| 6 | C | -4.83611800 | -0.61183200 | 0.48671400 |
| 7 | C | -1.19852400 | 0.27834100 | -0.55804700 |
| 8 | C | -0.64723000 | -1.15378800 | -0.70148400 |
| 9 | C | -1.20149200 | -2.06523500 | 0.42157800 |
| 10 | C | -2.73506100 | -2.01672200 | 0.49674700 |
| 11 | C | -2.69246300 | 2.73536400 | -1.39060800 |
| 12 | C | -2.92504500 | 0.00200100 | 2.00333200 |
| 13 | C | 0.86762900 | -1.26711000 | -0.80704300 |
| 14 | C | 1.69765800 | -0.37071400 | -0.23561500 |
| 15 | C | 1.34053100 | -2.46312100 | -1.59599900 |
| 16 | C | 3.15351100 | -0.37972900 | -0.24867600 |
| 17 | C | 3.90143600 | 0.56782800 | 0.33885900 |
| 18 | C | 5.41051400 | 0.65106700 | 0.39454400 |
| 19 | O | 5.79792900 | 0.79252000 | 1.78708100 |
| 20 | C | 6.12789100 | -0.56225300 | -0.20555500 |
| 21 | C | 5.88916700 | 1.94805500 | -0.26845600 |
| 22 | H | -6.47033000 | 0.77451100 | 0.16054100 |
| 23 | H | -5.57567200 | 1.09510200 | 1.61967100 |
| 24 | H | -5.09688700 | 2.81236900 | -0.26354500 |
| 25 | H | -3.02871200 | -0.26951800 | -1.47440800 |
| 26 | H | -5.08711800 | -1.06019100 | -0.48191400 |
| 27 | H | -5.27043700 | -1.26081900 | 1.25550100 |
| 28 | H | -0.82688500 | 0.75129100 | 0.35587100 |
| 29 | H | -0.82665100 | 0.87830300 | -1.39220800 |
| 30 | H | -1.05176300 | -1.54476200 | -1.64692900 |
| 31 | H | -0.88032200 | -3.09855700 | 0.25847900 |
| 32 | H | -0.76169300 | -1.75460000 | 1.37518900 |
| 33 | H | -3.14920100 | -2.47561300 | -0.41028100 |
| 34 | H | -3.08348800 | -2.62448000 | 1.34008000 |
| 35 | H | -2.42484600 | 2.42368200 | -2.40723300 |
| 36 | H | -3.31928900 | 3.62652600 | -1.46510500 |
| 37 | H | -1.75784600 | 3.01928200 | -0.89573500 |
| 38 | H | -3.41542100 | -0.57312900 | 2.79544300 |
| 39 | H | -1.85146200 | -0.03275400 | 2.19327000 |
| 40 | H | -3.23823900 | 1.04305000 | 2.09994600 |
| 41 | H | 1.25821300 | 0.46682600 | 0.29958900 |
| 42 | H | 2.41685500 | -2.62183800 | -1.54627000 |
| 43 | H | 0.85170300 | -3.37810100 | -1.24605300 |
| 44 | H | 1.06515600 | -2.35019000 | -2.65141400 |
| 45 | H | 3.64995900 | -1.19420800 | -0.76555200 |
| 46 | H | 3.40425300 | 1.38760700 | 0.85663000 |
| 47 | H | 5.45077800 | 0.02533400 | 2.25722500 |
| 48 | H | 7.20525400 | -0.44273000 | -0.07394800 |
| 49 | H | 5.82293300 | -1.48512200 | 0.29541200 |
| 50 | H | 5.92127800 | -0.66837800 | -1.27340700 |
| 51 | H | 6.96813100 | 2.05918700 | -0.13119600 |
| 52 | H | 5.66925700 | 1.93995700 | -1.33793700 |
| 53 | H | 5.39366700 | 2.81047300 | 0.18358100 |

B3LYP/6-311G(d,p) Energy = -856.8298408 a.u.; Population = 1.18% .

| Compound **8**  Conformer 13 | | Standard Orientation  (Ångstroms) | | |
| --- | --- | --- | --- | --- |
| 1 | C | -5.45118000 | 0.80819800 | 0.55573000 |
| 2 | C | -4.62862600 | 1.83781900 | -0.17329900 |
| 3 | C | -3.40068300 | 1.63125200 | -0.66435900 |
| 4 | C | -2.73665400 | 0.25948300 | -0.55509800 |
| 5 | C | -3.30484800 | -0.57778700 | 0.62943800 |
| 6 | C | -4.83990200 | -0.59922700 | 0.48553500 |
| 7 | C | -1.19476700 | 0.26656600 | -0.55280200 |
| 8 | C | -0.64785200 | -1.16874100 | -0.68117900 |
| 9 | C | -1.21112300 | -2.06835400 | 0.44712300 |
| 10 | C | -2.74474700 | -2.01293300 | 0.51480300 |
| 11 | C | -2.67503000 | 2.72268200 | -1.41232800 |
| 12 | C | -2.93143200 | 0.01862500 | 2.00392700 |
| 13 | C | 0.86716800 | -1.28827000 | -0.77838700 |
| 14 | C | 1.69729500 | -0.38022300 | -0.22580900 |
| 15 | C | 1.34060800 | -2.50309200 | -1.53782200 |
| 16 | C | 3.15319500 | -0.39056700 | -0.23545100 |
| 17 | C | 3.89959900 | 0.56995200 | 0.33261700 |
| 18 | C | 5.40841100 | 0.66619300 | 0.37000600 |
| 19 | O | 5.78532600 | 1.96255500 | -0.16580000 |
| 20 | C | 5.89696700 | 0.69684200 | 1.82281100 |
| 21 | C | 6.12634100 | -0.43254800 | -0.42016400 |
| 22 | H | -6.46679900 | 0.79171400 | 0.14244600 |
| 23 | H | -5.57544200 | 1.12030900 | 1.60183700 |
| 24 | H | -5.08292200 | 2.81965300 | -0.29421700 |
| 25 | H | -3.02379400 | -0.28127300 | -1.47189300 |
| 26 | H | -5.08952700 | -1.05444700 | -0.48024000 |
| 27 | H | -5.27980100 | -1.23989900 | 1.25810400 |
| 28 | H | -0.82531600 | 0.74589800 | 0.35868300 |
| 29 | H | -0.81706200 | 0.85798100 | -1.39042900 |
| 30 | H | -1.04899200 | -1.56695400 | -1.62513300 |
| 31 | H | -0.89401300 | -3.10441200 | 0.29435000 |
| 32 | H | -0.77449300 | -1.75169300 | 1.40022300 |
| 33 | H | -3.15675800 | -2.47740900 | -0.39035600 |
| 34 | H | -3.09939600 | -2.61252900 | 1.36137900 |
| 35 | H | -2.40530800 | 2.40115800 | -2.42532400 |
| 36 | H | -3.29776200 | 3.61582900 | -1.49660600 |
| 37 | H | -1.74087100 | 3.00688800 | -0.91674600 |
| 38 | H | -3.42697000 | -0.54806300 | 2.79889600 |
| 39 | H | -1.85866400 | -0.01937000 | 2.19772400 |
| 40 | H | -3.24025900 | 1.06177700 | 2.09128200 |
| 41 | H | 1.25786500 | 0.47029100 | 0.28839100 |
| 42 | H | 2.41666700 | -2.66132300 | -1.48164900 |
| 43 | H | 0.85056000 | -3.40948900 | -1.16806200 |
| 44 | H | 1.06801800 | -2.41457900 | -2.59630800 |
| 45 | H | 3.65112100 | -1.21896900 | -0.72830400 |
| 46 | H | 3.40074000 | 1.40477900 | 0.82399700 |
| 47 | H | 5.43272900 | 2.01200100 | -1.06214400 |
| 48 | H | 6.97505600 | 0.87695300 | 1.84842000 |
| 49 | H | 5.40038400 | 1.49932300 | 2.37334600 |
| 50 | H | 5.68674900 | -0.25135400 | 2.32192000 |
| 51 | H | 7.20315100 | -0.25551300 | -0.38235700 |
| 52 | H | 5.92568500 | -1.42452200 | -0.00788200 |
| 53 | H | 5.81638100 | -0.42842000 | -1.46870900 |

B3LYP/6-311G(d,p) Energy = -856.8298326 a.u.; Population = 1.17% .

| Compound **8**  Conformer 14 | | Standard Orientation  (Ångstroms) | | |
| --- | --- | --- | --- | --- |
| 1 | C | -5.62743900 | -0.72117800 | 0.47823100 |
| 2 | C | -5.23326800 | 0.70595300 | 0.75494700 |
| 3 | C | -4.04786100 | 1.24730000 | 0.44957200 |
| 4 | C | -2.98074000 | 0.42935000 | -0.27617700 |
| 5 | C | -3.15579500 | -1.10099100 | -0.05083700 |
| 6 | C | -4.61208100 | -1.45772300 | -0.40917100 |
| 7 | C | -1.52552200 | 0.87842800 | -0.04075600 |
| 8 | C | -0.55211000 | 0.12492500 | -0.98585600 |
| 9 | C | -0.71783500 | -1.39517100 | -0.83061100 |
| 10 | C | -2.18138700 | -1.83313000 | -0.99943000 |
| 11 | C | -3.76584300 | 2.70147800 | 0.73870100 |
| 12 | C | -2.87645100 | -1.51731600 | 1.40961300 |
| 13 | C | 0.87000200 | 0.65270000 | -0.85526000 |
| 14 | C | 1.88963300 | -0.11103600 | -0.41135200 |
| 15 | C | 1.04553100 | 2.09922400 | -1.26161600 |
| 16 | C | 3.27728700 | 0.29587500 | -0.23872500 |
| 17 | C | 4.23758000 | -0.52858100 | 0.20882400 |
| 18 | C | 5.69956200 | -0.21035200 | 0.43004400 |
| 19 | O | 6.04152300 | -0.60205800 | 1.78576400 |
| 20 | C | 6.57095500 | -1.10656100 | -0.45806300 |
| 21 | C | 6.05782100 | 1.26548000 | 0.22801600 |
| 22 | H | -6.61648100 | -0.74218100 | 0.00497600 |
| 23 | H | -5.75786700 | -1.25091500 | 1.43182400 |
| 24 | H | -5.98133400 | 1.32544300 | 1.24644900 |
| 25 | H | -3.16013500 | 0.59632100 | -1.35106100 |
| 26 | H | -4.78535900 | -1.18870900 | -1.45783600 |
| 27 | H | -4.76172500 | -2.54067000 | -0.33362700 |
| 28 | H | -1.22478100 | 0.71534900 | 1.00033400 |
| 29 | H | -1.44238300 | 1.95111400 | -0.22328100 |
| 30 | H | -0.87338900 | 0.37755400 | -2.00896300 |
| 31 | H | -0.09668000 | -1.91156400 | -1.56958400 |
| 32 | H | -0.35324700 | -1.71179000 | 0.15144800 |
| 33 | H | -2.49424500 | -1.64472700 | -2.03451300 |
| 34 | H | -2.26189300 | -2.91510600 | -0.84253400 |
| 35 | H | -3.49580900 | 3.24554400 | -0.17423800 |
| 36 | H | -4.64136900 | 3.18801700 | 1.17374600 |
| 37 | H | -2.92910300 | 2.82527200 | 1.43416400 |
| 38 | H | -3.09424900 | -2.58267500 | 1.53755300 |
| 39 | H | -1.83628500 | -1.36162300 | 1.69854900 |
| 40 | H | -3.49559400 | -0.96041900 | 2.11502400 |
| 41 | H | 1.68589200 | -1.14322400 | -0.14290500 |
| 42 | H | 2.07294700 | 2.33888000 | -1.53488500 |
| 43 | H | 0.40842800 | 2.33617500 | -2.11897700 |
| 44 | H | 0.75471900 | 2.78058500 | -0.45437200 |
| 45 | H | 3.53733600 | 1.31995500 | -0.48481000 |
| 46 | H | 3.97596000 | -1.55524300 | 0.46354600 |
| 47 | H | 5.46558400 | -0.10519000 | 2.37867700 |
| 48 | H | 7.62672200 | -0.94935100 | -0.22149800 |
| 49 | H | 6.33211900 | -2.15870200 | -0.28628500 |
| 50 | H | 6.40914400 | -0.87951500 | -1.51376700 |
| 51 | H | 7.11417600 | 1.41221700 | 0.46233100 |
| 52 | H | 5.88575700 | 1.58734800 | -0.80205900 |
| 53 | H | 5.46871900 | 1.90625000 | 0.88989700 |

B3LYP/6-311G(d,p) Energy = -856.8294244 a.u.; Population = 0.76% .

| Compound **8**  Conformer 15 | | Standard Orientation  (Ångstroms) | | |
| --- | --- | --- | --- | --- |
| 1 | C | -5.62085900 | -0.73898900 | 0.47896000 |
| 2 | C | -5.22953700 | 0.68249100 | 0.78719200 |
| 3 | C | -4.04768600 | 1.23483400 | 0.48751700 |
| 4 | C | -2.98192000 | 0.43763300 | -0.26284200 |
| 5 | C | -3.15194100 | -1.09816300 | -0.07477000 |
| 6 | C | -4.60938200 | -1.44967800 | -0.43345200 |
| 7 | C | -1.52682300 | 0.88464800 | -0.02234800 |
| 8 | C | -0.55390600 | 0.15705900 | -0.98825800 |
| 9 | C | -0.71769300 | -1.36695100 | -0.87340000 |
| 10 | C | -2.18111700 | -1.80279600 | -1.04741300 |
| 11 | C | -3.76902200 | 2.68312400 | 0.80790200 |
| 12 | C | -2.86292200 | -1.54955300 | 1.37337000 |
| 13 | C | 0.86823700 | 0.68179300 | -0.84362100 |
| 14 | C | 1.88795700 | -0.09473700 | -0.42262100 |
| 15 | C | 1.04367800 | 2.13935700 | -1.20740100 |
| 16 | C | 3.27586300 | 0.30521900 | -0.23599900 |
| 17 | C | 4.23221200 | -0.53327600 | 0.19365500 |
| 18 | C | 5.69745000 | -0.22999200 | 0.41411800 |
| 19 | O | 6.47092700 | -1.20198500 | -0.33840400 |
| 20 | C | 6.11656000 | 1.18145100 | -0.00867700 |
| 21 | C | 6.07261000 | -0.49246500 | 1.87751700 |
| 22 | H | -6.61330000 | -0.75213200 | 0.01259100 |
| 23 | H | -5.74223700 | -1.29242200 | 1.42021400 |
| 24 | H | -5.97666000 | 1.28791400 | 1.29730400 |
| 25 | H | -3.16642800 | 0.63087300 | -1.33247200 |
| 26 | H | -4.79000600 | -1.15378900 | -1.47360700 |
| 27 | H | -4.75573100 | -2.53462100 | -0.38506100 |
| 28 | H | -1.22184000 | 0.69805000 | 1.01352500 |
| 29 | H | -1.44734800 | 1.96157500 | -0.17963100 |
| 30 | H | -0.87600100 | 0.43675000 | -2.00407200 |
| 31 | H | -0.09881900 | -1.86264100 | -1.62829900 |
| 32 | H | -0.34882500 | -1.70923400 | 0.09833100 |
| 33 | H | -2.49933600 | -1.58689300 | -2.07551600 |
| 34 | H | -2.25895500 | -2.88875900 | -0.91959400 |
| 35 | H | -3.50619400 | 3.24850500 | -0.09414300 |
| 36 | H | -4.64376000 | 3.15659100 | 1.25867400 |
| 37 | H | -2.92872200 | 2.79445300 | 1.50116900 |
| 38 | H | -3.07959300 | -2.61774900 | 1.47770300 |
| 39 | H | -1.82107300 | -1.40009900 | 1.65930100 |
| 40 | H | -3.47760800 | -1.00964300 | 2.09567200 |
| 41 | H | 1.68367800 | -1.13426100 | -0.18488800 |
| 42 | H | 0.41766400 | 2.39754900 | -2.06696000 |
| 43 | H | 0.73870000 | 2.79731500 | -0.38613100 |
| 44 | H | 2.07382000 | 2.38998500 | -1.45950700 |
| 45 | H | 3.53808900 | 1.33612000 | -0.44901200 |
| 46 | H | 3.96753700 | -1.56735000 | 0.41247300 |
| 47 | H | 6.21179200 | -1.11518000 | -1.26335400 |
| 48 | H | 7.19299700 | 1.29384900 | 0.13605800 |
| 49 | H | 5.89424800 | 1.35635700 | -1.06485400 |
| 50 | H | 5.60791600 | 1.94871500 | 0.58013400 |
| 51 | H | 7.15165300 | -0.37785800 | 2.01073500 |
| 52 | H | 5.55988400 | 0.20789200 | 2.54003300 |
| 53 | H | 5.79650200 | -1.51002500 | 2.16384800 |

B3LYP/6-311G(d,p) Energy = -856.8294089 a.u.; Population = 0.74% .

| Compound **8**  Conformer 16 | | Standard Orientation  (Ångstroms) | | |
| --- | --- | --- | --- | --- |
| 1 | C | -5.76000700 | 0.07766100 | -0.61146100 |
| 2 | C | -5.13465600 | 1.40183700 | -0.26010900 |
| 3 | C | -3.82084900 | 1.61731200 | -0.12230100 |
| 4 | C | -2.81438500 | 0.49454100 | -0.36922500 |
| 5 | C | -3.44439900 | -0.91623500 | -0.17372200 |
| 6 | C | -4.72360200 | -0.97542200 | -1.03246800 |
| 7 | C | -1.47638900 | 0.62493400 | 0.38553100 |
| 8 | C | -0.46474200 | -0.43424800 | -0.11247100 |
| 9 | C | -1.05306500 | -1.85980800 | 0.00180600 |
| 10 | C | -2.42900500 | -1.96754700 | -0.67342800 |
| 11 | C | -3.29394800 | 2.99476800 | 0.19808000 |
| 12 | C | -3.80830300 | -1.19942500 | 1.30009400 |
| 13 | C | 0.89885100 | -0.31722100 | 0.54944800 |
| 14 | C | 1.99566500 | -0.16115800 | -0.22167600 |
| 15 | C | 0.94138700 | -0.38618300 | 2.05624700 |
| 16 | C | 3.38142600 | -0.05053100 | 0.20995900 |
| 17 | C | 4.40879700 | 0.10479600 | -0.63890800 |
| 18 | C | 5.87947100 | 0.22957700 | -0.30059600 |
| 19 | O | 6.36020400 | 1.50385000 | -0.80626700 |
| 20 | C | 6.17216400 | 0.27140500 | 1.19714800 |
| 21 | C | 6.66607900 | -0.90341200 | -0.98021900 |
| 22 | H | -6.49079700 | 0.22065000 | -1.41655700 |
| 23 | H | -6.34751100 | -0.28253200 | 0.24427200 |
| 24 | H | -5.82066900 | 2.23273000 | -0.10507900 |
| 25 | H | -2.55241600 | 0.56150500 | -1.43790600 |
| 26 | H | -4.44379300 | -0.80851400 | -2.07952200 |
| 27 | H | -5.16289700 | -1.97799600 | -0.98073500 |
| 28 | H | -1.63743900 | 0.52896000 | 1.46378100 |
| 29 | H | -1.04620100 | 1.61652300 | 0.22135100 |
| 30 | H | -0.31618700 | -0.24191600 | -1.18261100 |
| 31 | H | -0.36052800 | -2.57051000 | -0.46081300 |
| 32 | H | -1.13251000 | -2.14954900 | 1.05388700 |
| 33 | H | -2.30494100 | -1.84335300 | -1.75688800 |
| 34 | H | -2.83840100 | -2.97309200 | -0.52089800 |
| 35 | H | -2.58878400 | 3.34066800 | -0.56702000 |
| 36 | H | -4.10920900 | 3.71894700 | 0.25704400 |
| 37 | H | -2.75438200 | 3.01501200 | 1.15076600 |
| 38 | H | -4.32202600 | -2.16346000 | 1.37431600 |
| 39 | H | -2.93045200 | -1.24790000 | 1.94593600 |
| 40 | H | -4.47008900 | -0.43335400 | 1.70833100 |
| 41 | H | 1.84616700 | -0.11191000 | -1.29942000 |
| 42 | H | 1.95381600 | -0.47969900 | 2.44668900 |
| 43 | H | 0.49874900 | 0.51226500 | 2.49989500 |
| 44 | H | 0.36238800 | -1.23563500 | 2.43043100 |
| 45 | H | 3.58424200 | -0.10015000 | 1.27453300 |
| 46 | H | 4.19740500 | 0.14971500 | -1.70842200 |
| 47 | H | 6.22655200 | 1.50937900 | -1.76125000 |
| 48 | H | 7.24385600 | 0.41393700 | 1.35072300 |
| 49 | H | 5.64454300 | 1.10246900 | 1.66951000 |
| 50 | H | 5.87472800 | -0.65834000 | 1.68567700 |
| 51 | H | 7.73730100 | -0.76603100 | -0.81152800 |
| 52 | H | 6.36936000 | -1.88006300 | -0.58901500 |
| 53 | H | 6.48554900 | -0.90340400 | -2.05970400 |

B3LYP/6-311G(d,p) Energy = -856.8294084 a.u.; Population = 0.74% .

| Compound **8**  Conformer 17 | | Standard Orientation  (Ångstroms) | | |
| --- | --- | --- | --- | --- |
| 1 | C | -5.76731200 | 0.05975900 | -0.59102600 |
| 2 | C | -5.15195200 | 1.38572800 | -0.22884400 |
| 3 | C | -3.83921500 | 1.61229300 | -0.09884800 |
| 4 | C | -2.82364300 | 0.50224900 | -0.36570800 |
| 5 | C | -3.43924300 | -0.91672000 | -0.18438900 |
| 6 | C | -4.72395000 | -0.97682700 | -1.03479100 |
| 7 | C | -1.48310900 | 0.63575800 | 0.38388100 |
| 8 | C | -0.46371600 | -0.40815700 | -0.12989200 |
| 9 | C | -1.03876700 | -1.84082100 | -0.03554700 |
| 10 | C | -2.41744500 | -1.95144000 | -0.70467500 |
| 11 | C | -3.32306400 | 2.99116900 | 0.23292800 |
| 12 | C | -3.78996300 | -1.22275500 | 1.28813700 |
| 13 | C | 0.89848800 | -0.29206400 | 0.53493600 |
| 14 | C | 1.99907600 | -0.14989400 | -0.23332500 |
| 15 | C | 0.93553600 | -0.34988600 | 2.04252700 |
| 16 | C | 3.38381100 | -0.04878300 | 0.20397900 |
| 17 | C | 4.41742000 | 0.08826600 | -0.64039200 |
| 18 | C | 5.88823700 | 0.19221600 | -0.29466400 |
| 19 | O | 6.59732600 | -0.86669500 | -0.99113100 |
| 20 | C | 6.43900600 | 1.54974500 | -0.76161200 |
| 21 | C | 6.19695500 | -0.04103400 | 1.18228100 |
| 22 | H | -6.50708300 | 0.20537400 | -1.38738900 |
| 23 | H | -6.34264500 | -0.31765800 | 0.26550000 |
| 24 | H | -5.84459900 | 2.20820100 | -0.05900900 |
| 25 | H | -2.56832300 | 0.58535500 | -1.43492200 |
| 26 | H | -4.45370400 | -0.79230900 | -2.08140700 |
| 27 | H | -5.15281900 | -1.98442600 | -0.99399100 |
| 28 | H | -1.63879900 | 0.52647700 | 1.46163800 |
| 29 | H | -1.06267100 | 1.63309400 | 0.22919300 |
| 30 | H | -0.31662000 | -0.19991000 | -1.19726300 |
| 31 | H | -0.34197100 | -2.53677700 | -0.51377900 |
| 32 | H | -1.10834000 | -2.14876200 | 1.01214400 |
| 33 | H | -2.30058900 | -1.81073400 | -1.78692400 |
| 34 | H | -2.81675900 | -2.96278100 | -0.56416000 |
| 35 | H | -2.62694700 | 3.35215700 | -0.53349400 |
| 36 | H | -4.14475600 | 3.70677100 | 0.30579200 |
| 37 | H | -2.77670200 | 3.00621000 | 1.18181500 |
| 38 | H | -4.29509700 | -2.19200100 | 1.35296400 |
| 39 | H | -2.90715800 | -1.27259500 | 1.92703400 |
| 40 | H | -4.45506400 | -0.46792500 | 1.71163600 |
| 41 | H | 1.85428200 | -0.10788200 | -1.31205800 |
| 42 | H | 1.94258100 | -0.48330400 | 2.43566100 |
| 43 | H | 0.53097800 | 0.57035700 | 2.47807000 |
| 44 | H | 0.32045400 | -1.17077400 | 2.42253900 |
| 45 | H | 3.57981700 | -0.08581300 | 1.27037700 |
| 46 | H | 4.21212800 | 0.13507600 | -1.71100200 |
| 47 | H | 6.47163200 | -0.73000300 | -1.93738900 |
| 48 | H | 7.51863400 | 1.59049300 | -0.59519600 |
| 49 | H | 6.25029200 | 1.69427100 | -1.82999200 |
| 50 | H | 5.96691200 | 2.37420500 | -0.22116600 |
| 51 | H | 7.27782100 | -0.00667400 | 1.33378600 |
| 52 | H | 5.73711500 | 0.72450600 | 1.80992600 |
| 53 | H | 5.83649000 | -1.02146500 | 1.50037600 |

B3LYP/6-311G(d,p) Energy = -856.8293891 a.u.; Population = 0.73% .

| Compound **8**  Conformer 18 | | Standard Orientation  (Ångstroms) | | |
| --- | --- | --- | --- | --- |
| 1 | C | -5.76053400 | 0.06004600 | -0.61431800 |
| 2 | C | -5.14036700 | 1.39008000 | -0.27616900 |
| 3 | C | -3.82749500 | 1.61176500 | -0.13933400 |
| 4 | C | -2.81677200 | 0.49017400 | -0.37381800 |
| 5 | C | -3.44182600 | -0.92077600 | -0.16371200 |
| 6 | C | -4.71990700 | -0.99366900 | -1.02312900 |
| 7 | C | -1.47945800 | 0.63347200 | 0.37983100 |
| 8 | C | -0.46389300 | -0.42746700 | -0.10619900 |
| 9 | C | -1.04724200 | -1.85372900 | 0.02442700 |
| 10 | C | -2.42209500 | -1.97370800 | -0.65094700 |
| 11 | C | -3.30587900 | 2.99430400 | 0.16738400 |
| 12 | C | -3.80633000 | -1.18947900 | 1.31262300 |
| 13 | C | 0.89937500 | -0.29676600 | 0.55375600 |
| 14 | C | 1.99487600 | -0.14539100 | -0.21988200 |
| 15 | C | 0.94309000 | -0.34753200 | 2.06128400 |
| 16 | C | 3.38065200 | -0.01974700 | 0.20828300 |
| 17 | C | 4.40287000 | 0.12602600 | -0.64734100 |
| 18 | C | 5.87282900 | 0.22426500 | -0.31401000 |
| 19 | O | 6.34251300 | 1.34616700 | -1.10301600 |
| 20 | C | 6.15999600 | 0.49479900 | 1.16620800 |
| 21 | C | 6.59313300 | -1.05246500 | -0.78680800 |
| 22 | H | -6.49093100 | 0.19190100 | -1.42166700 |
| 23 | H | -6.34771200 | -0.29319200 | 0.24455700 |
| 24 | H | -5.82956600 | 2.22000100 | -0.13032900 |
| 25 | H | -2.55438900 | 0.54655000 | -1.44298500 |
| 26 | H | -4.43960600 | -0.83677900 | -2.07160500 |
| 27 | H | -5.15570900 | -1.99720900 | -0.96133900 |
| 28 | H | -1.64013400 | 0.54888800 | 1.45911100 |
| 29 | H | -1.05286700 | 1.62475000 | 0.20473000 |
| 30 | H | -0.31632900 | -0.24693700 | -1.17853300 |
| 31 | H | -0.35186400 | -2.56735600 | -0.42935100 |
| 32 | H | -1.12662000 | -2.13140300 | 1.07978900 |
| 33 | H | -2.29713500 | -1.86061400 | -1.73552200 |
| 34 | H | -2.82809800 | -2.97902300 | -0.48818100 |
| 35 | H | -2.60198100 | 3.33522900 | -0.60109900 |
| 36 | H | -4.12386200 | 3.71595600 | 0.21918800 |
| 37 | H | -2.76640200 | 3.02592400 | 1.11980800 |
| 38 | H | -4.31815100 | -2.15375300 | 1.39630400 |
| 39 | H | -2.92885500 | -1.22953000 | 1.95953000 |
| 40 | H | -4.47004400 | -0.42061900 | 1.71236400 |
| 41 | H | 1.84484500 | -0.11177200 | -1.29811100 |
| 42 | H | 1.95622100 | -0.43313200 | 2.45177700 |
| 43 | H | 0.49758000 | 0.55440600 | 2.49487000 |
| 44 | H | 0.36733900 | -1.19468800 | 2.44564000 |
| 45 | H | 3.58537300 | -0.04780800 | 1.27344800 |
| 46 | H | 4.19958900 | 0.16020700 | -1.71666800 |
| 47 | H | 7.30014700 | 1.39069400 | -0.99794500 |
| 48 | H | 7.23735300 | 0.61053500 | 1.31550500 |
| 49 | H | 5.66769800 | 1.41453700 | 1.48824400 |
| 50 | H | 5.82340800 | -0.32695100 | 1.80285600 |
| 51 | H | 7.67411400 | -0.95483900 | -0.63985500 |
| 52 | H | 6.25078500 | -1.92774900 | -0.22933400 |
| 53 | H | 6.40555300 | -1.22077500 | -1.84983900 |

B3LYP/6-311G(d,p) Energy = -856.8293192 a.u.; Population = 0.68% .

| Compound **8**  Conformer 19 | | Standard Orientation  (Ångstroms) | | |
| --- | --- | --- | --- | --- |
| 1 | C | -5.76472000 | 0.06727800 | -0.60005900 |
| 2 | C | -5.14870900 | 1.39017300 | -0.22795200 |
| 3 | C | -3.83610200 | 1.61396300 | -0.09159700 |
| 4 | C | -2.82132700 | 0.50387000 | -0.36119700 |
| 5 | C | -3.43975200 | -0.91509600 | -0.18894000 |
| 6 | C | -4.72133200 | -0.96902600 | -1.04447400 |
| 7 | C | -1.48249200 | 0.63144800 | 0.39254500 |
| 8 | C | -0.46377500 | -0.41105200 | -0.12522500 |
| 9 | C | -1.04092100 | -1.84318700 | -0.03718800 |
| 10 | C | -2.41793200 | -1.94924900 | -0.71038600 |
| 11 | C | -3.31923100 | 2.99001600 | 0.25042700 |
| 12 | C | -3.79631100 | -1.22745000 | 1.28083200 |
| 13 | C | 0.89923600 | -0.30012500 | 0.53905600 |
| 14 | C | 1.99872100 | -0.15757200 | -0.23048400 |
| 15 | C | 0.93833200 | -0.36541900 | 2.04617200 |
| 16 | C | 3.38581800 | -0.06625400 | 0.20199100 |
| 17 | C | 4.41324700 | 0.06481500 | -0.64970500 |
| 18 | C | 5.87906800 | 0.19258800 | -0.30758000 |
| 19 | O | 6.53806800 | -0.71319200 | -1.22775500 |
| 20 | C | 6.35428600 | 1.62871500 | -0.59759100 |
| 21 | C | 6.22078800 | -0.21370400 | 1.12954400 |
| 22 | H | -6.50055000 | 0.21844400 | -1.39904100 |
| 23 | H | -6.34481000 | -0.31350100 | 0.25176900 |
| 24 | H | -5.84072600 | 2.21271300 | -0.05597100 |
| 25 | H | -2.56298300 | 0.59213300 | -1.42924300 |
| 26 | H | -4.44657700 | -0.78013700 | -2.08910800 |
| 27 | H | -5.15203300 | -1.97605500 | -1.01006400 |
| 28 | H | -1.64088200 | 0.51595500 | 1.46928100 |
| 29 | H | -1.06041400 | 1.62911900 | 0.24463600 |
| 30 | H | -0.31656800 | -0.19845400 | -1.19169500 |
| 31 | H | -0.34399700 | -2.53863100 | -0.51596400 |
| 32 | H | -1.11366900 | -2.15439800 | 1.00933100 |
| 33 | H | -2.29784000 | -1.80458400 | -1.79174200 |
| 34 | H | -2.81919000 | -2.96051600 | -0.57496800 |
| 35 | H | -2.61980900 | 3.35476800 | -0.51118900 |
| 36 | H | -4.14013700 | 3.70636700 | 0.32473100 |
| 37 | H | -2.77627600 | 2.99843600 | 1.20136100 |
| 38 | H | -4.30208900 | -2.19674000 | 1.33958000 |
| 39 | H | -2.91590700 | -1.28048900 | 1.92282400 |
| 40 | H | -4.46266000 | -0.47415000 | 1.70508900 |
| 41 | H | 1.85184100 | -0.10977400 | -1.30864000 |
| 42 | H | 1.94744000 | -0.48715300 | 2.43776700 |
| 43 | H | 0.52156000 | 0.54681900 | 2.48691400 |
| 44 | H | 0.33476400 | -1.19665200 | 2.42244700 |
| 45 | H | 3.58736300 | -0.10844300 | 1.26728000 |
| 46 | H | 4.21460400 | 0.10798900 | -1.71954200 |
| 47 | H | 7.48863100 | -0.58996900 | -1.12150700 |
| 48 | H | 7.43643600 | 1.70733800 | -0.44724600 |
| 49 | H | 6.13109400 | 1.90011100 | -1.63204600 |
| 50 | H | 5.86489100 | 2.34690100 | 0.06479600 |
| 51 | H | 7.30202100 | -0.14994100 | 1.28160100 |
| 52 | H | 5.74387300 | 0.44162200 | 1.86236400 |
| 53 | H | 5.90668200 | -1.24199400 | 1.31910700 |

B3LYP/6-311G(d,p) Energy = -856.8292357 a.u.; Population = 0.62% .

| Compound **8**  Conformer 20 | | Standard Orientation  (Ångstroms) | | |
| --- | --- | --- | --- | --- |
| 1 | C | -5.45413500 | 0.79395300 | 0.57114200 |
| 2 | C | -4.64124000 | 1.82833300 | -0.16197500 |
| 3 | C | -3.41501800 | 1.62846600 | -0.66006000 |
| 4 | C | -2.74284700 | 0.26036300 | -0.55520900 |
| 5 | C | -3.30039600 | -0.58134800 | 0.63125500 |
| 6 | C | -4.83614800 | -0.61021800 | 0.49575200 |
| 7 | C | -1.20098100 | 0.27551600 | -0.56120600 |
| 8 | C | -0.64874300 | -1.15719900 | -0.69531600 |
| 9 | C | -1.20060300 | -2.06162500 | 0.43447100 |
| 10 | C | -2.73410700 | -2.01373000 | 0.51166900 |
| 11 | C | -2.69960500 | 2.72378700 | -1.41218800 |
| 12 | C | -2.92246400 | 0.01515100 | 2.00446900 |
| 13 | C | 0.86608500 | -1.26987300 | -0.80265000 |
| 14 | C | 1.69655400 | -0.37438000 | -0.23067400 |
| 15 | C | 1.33811200 | -2.46589000 | -1.59219100 |
| 16 | C | 3.15266300 | -0.38368800 | -0.24580800 |
| 17 | C | 3.90334600 | 0.55796500 | 0.34574000 |
| 18 | C | 5.41458200 | 0.64209900 | 0.39384100 |
| 19 | O | 5.82895400 | 0.65588900 | 1.78602100 |
| 20 | C | 6.12454600 | -0.56764400 | -0.20879400 |
| 21 | C | 5.88490000 | 1.94292500 | -0.27796900 |
| 22 | H | -6.47213800 | 0.77267600 | 0.16397900 |
| 23 | H | -5.57365100 | 1.10438700 | 1.61830900 |
| 24 | H | -5.10154600 | 2.80771300 | -0.28008300 |
| 25 | H | -3.03172600 | -0.28104200 | -1.47111200 |
| 26 | H | -5.08875200 | -1.06537600 | -0.46928600 |
| 27 | H | -5.26858800 | -1.25417400 | 1.26981300 |
| 28 | H | -0.82858100 | 0.75533200 | 0.34881600 |
| 29 | H | -0.83063200 | 0.86969200 | -1.40017700 |
| 30 | H | -1.05443800 | -1.55471800 | -1.63749900 |
| 31 | H | -0.87878000 | -3.09570400 | 0.27737700 |
| 32 | H | -0.75954000 | -1.74470000 | 1.38542300 |
| 33 | H | -3.14937500 | -2.47915900 | -0.39150700 |
| 34 | H | -3.08082300 | -2.61588500 | 1.35972100 |
| 35 | H | -2.43391900 | 2.40351800 | -2.42664800 |
| 36 | H | -3.32763900 | 3.61353900 | -1.49304200 |
| 37 | H | -1.76417500 | 3.01301000 | -0.92195800 |
| 38 | H | -3.41133500 | -0.55461600 | 2.80136100 |
| 39 | H | -1.84855100 | -0.01828300 | 2.19271600 |
| 40 | H | -3.23553300 | 1.05681900 | 2.09459900 |
| 41 | H | 1.25726000 | 0.46183000 | 0.30693700 |
| 42 | H | 2.41448000 | -2.62464800 | -1.54386500 |
| 43 | H | 0.84994000 | -3.38075800 | -1.24084300 |
| 44 | H | 1.06100400 | -2.35370800 | -2.64724400 |
| 45 | H | 3.64769600 | -1.19509500 | -0.76876100 |
| 46 | H | 3.39997000 | 1.37682700 | 0.86229800 |
| 47 | H | 5.43063700 | 1.42902700 | 2.20248200 |
| 48 | H | 7.20303800 | -0.45127600 | -0.08326700 |
| 49 | H | 5.81714400 | -1.48528700 | 0.29687500 |
| 50 | H | 5.91060000 | -0.66388000 | -1.27485600 |
| 51 | H | 6.96689700 | 2.05022000 | -0.16477000 |
| 52 | H | 5.63902800 | 1.94797600 | -1.34304400 |
| 53 | H | 5.40339100 | 2.81085000 | 0.18329000 |

B3LYP/6-311G(d,p) Energy =-856.8284953 a.u.; Population = 0.28% .


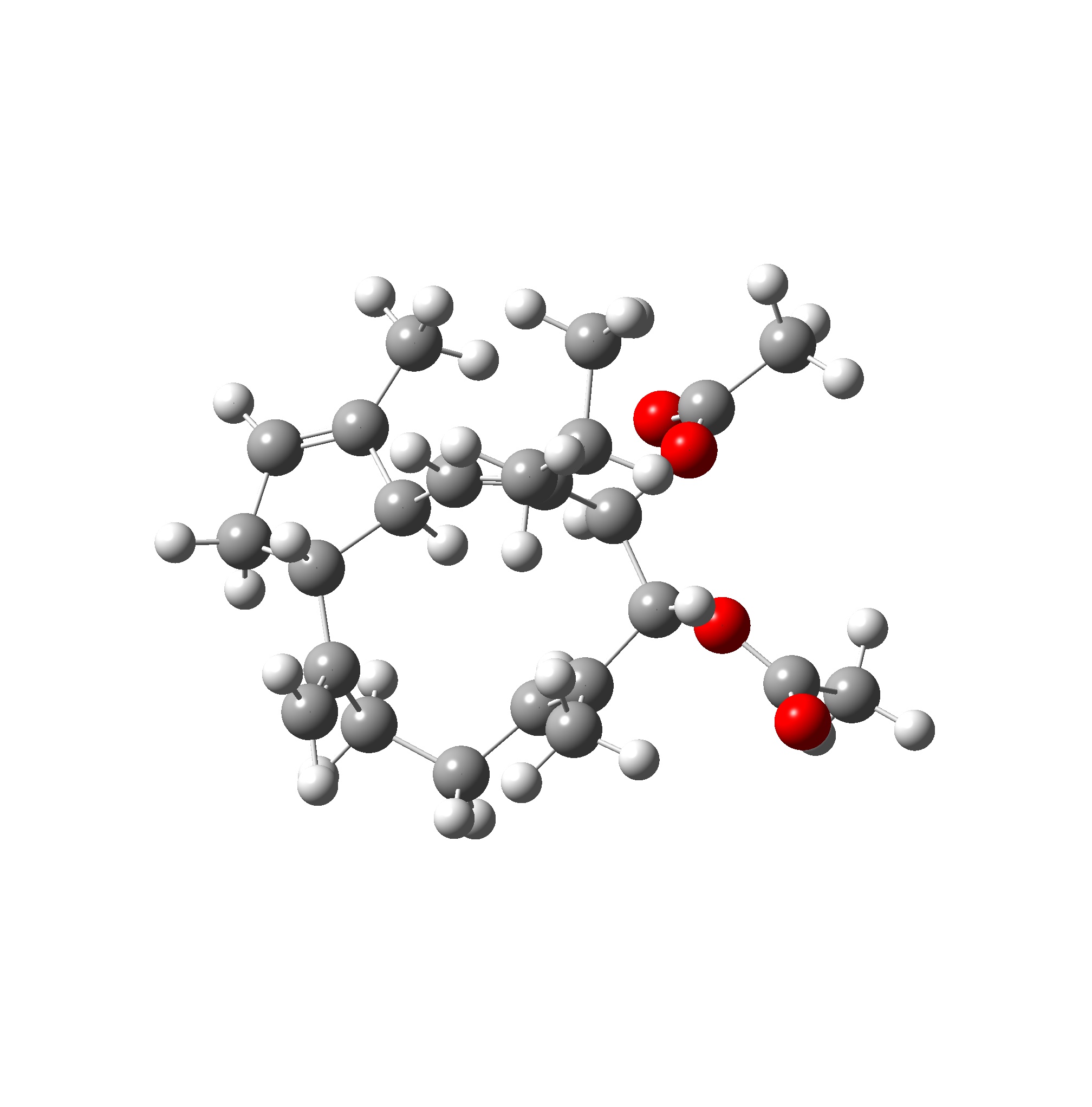

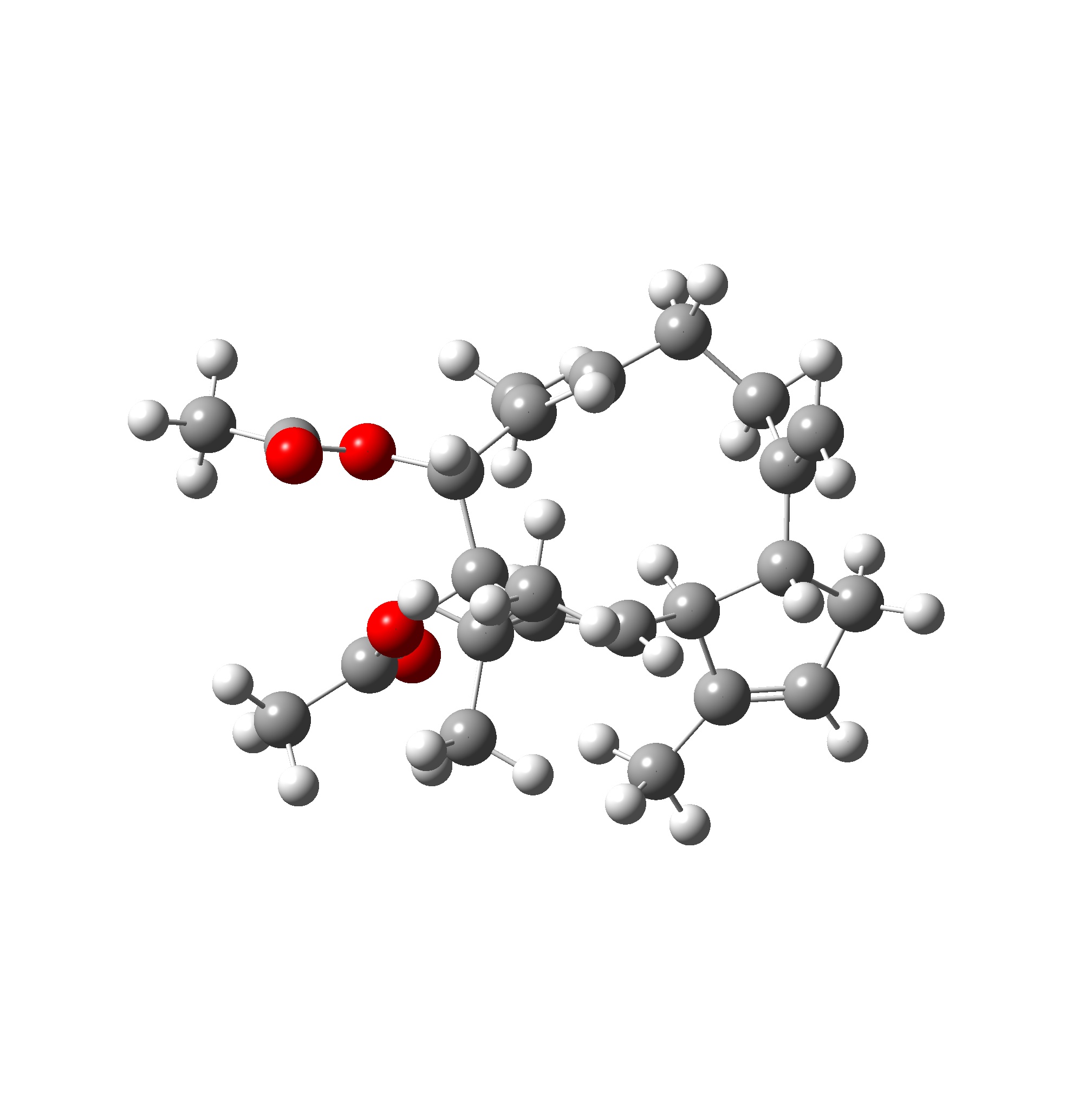

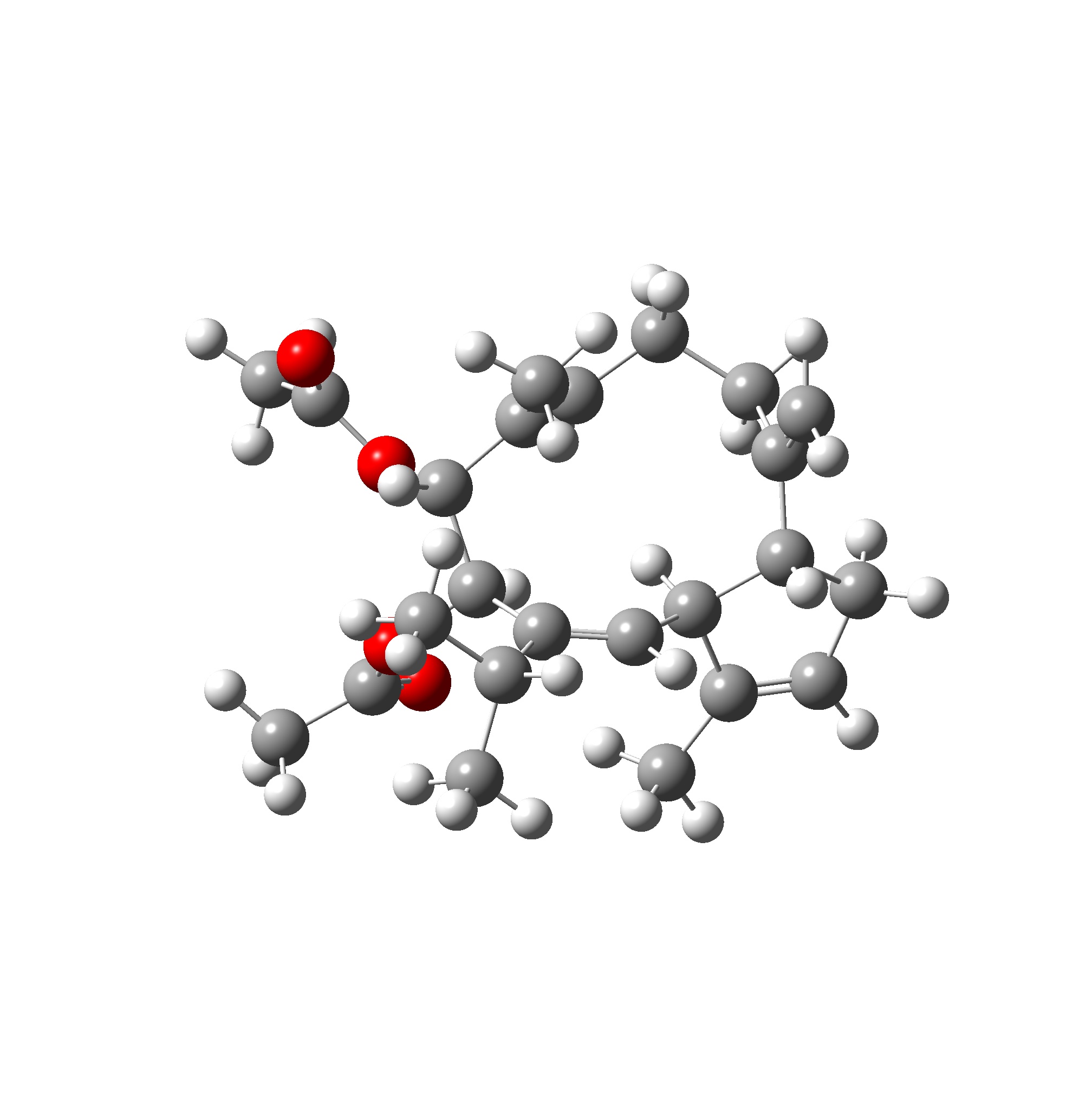

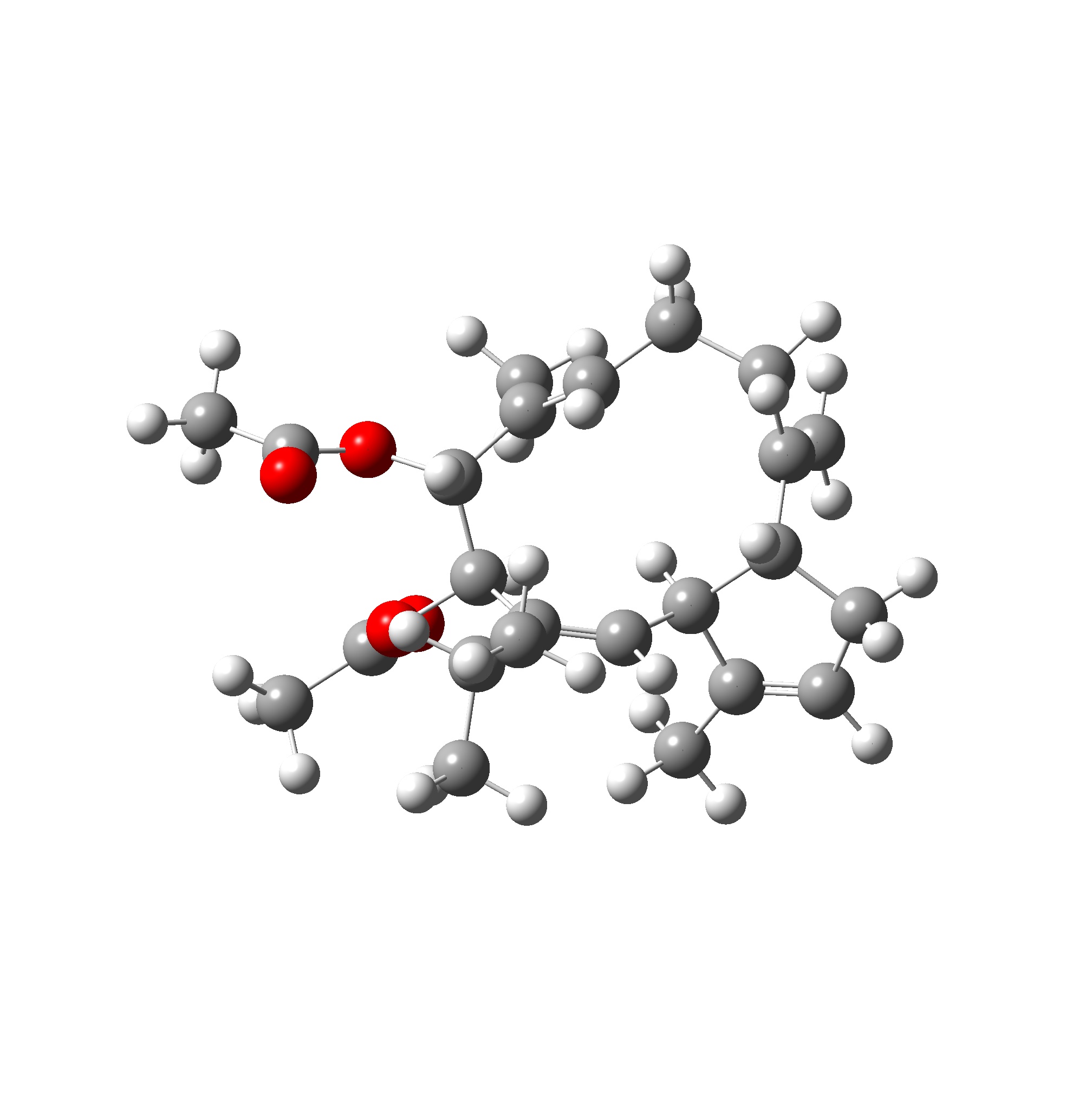


Conf. 1 Conf. 2 Conf. 3 Conf. 4

73.79% 16.19% 4.05% 2.60%


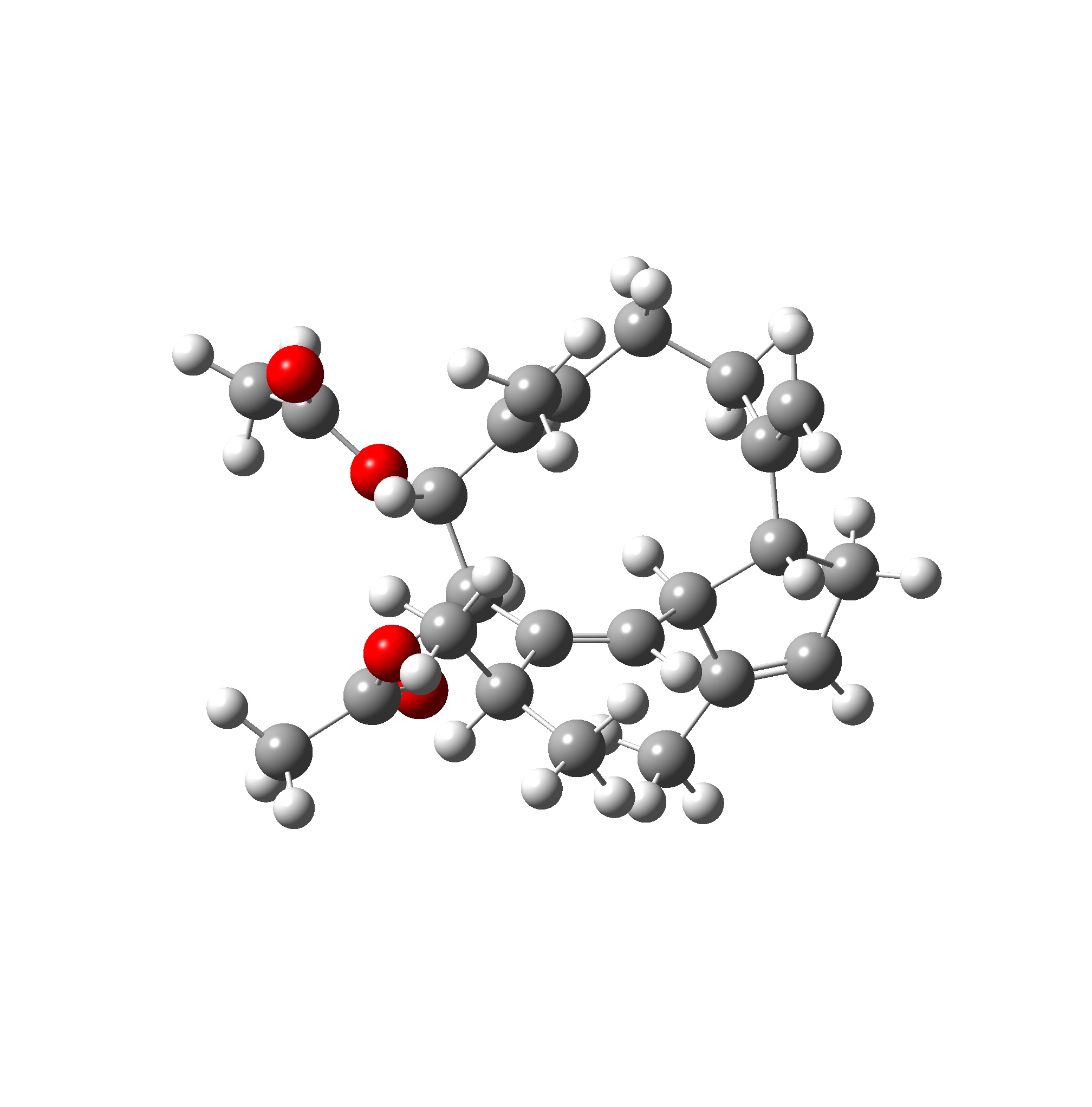

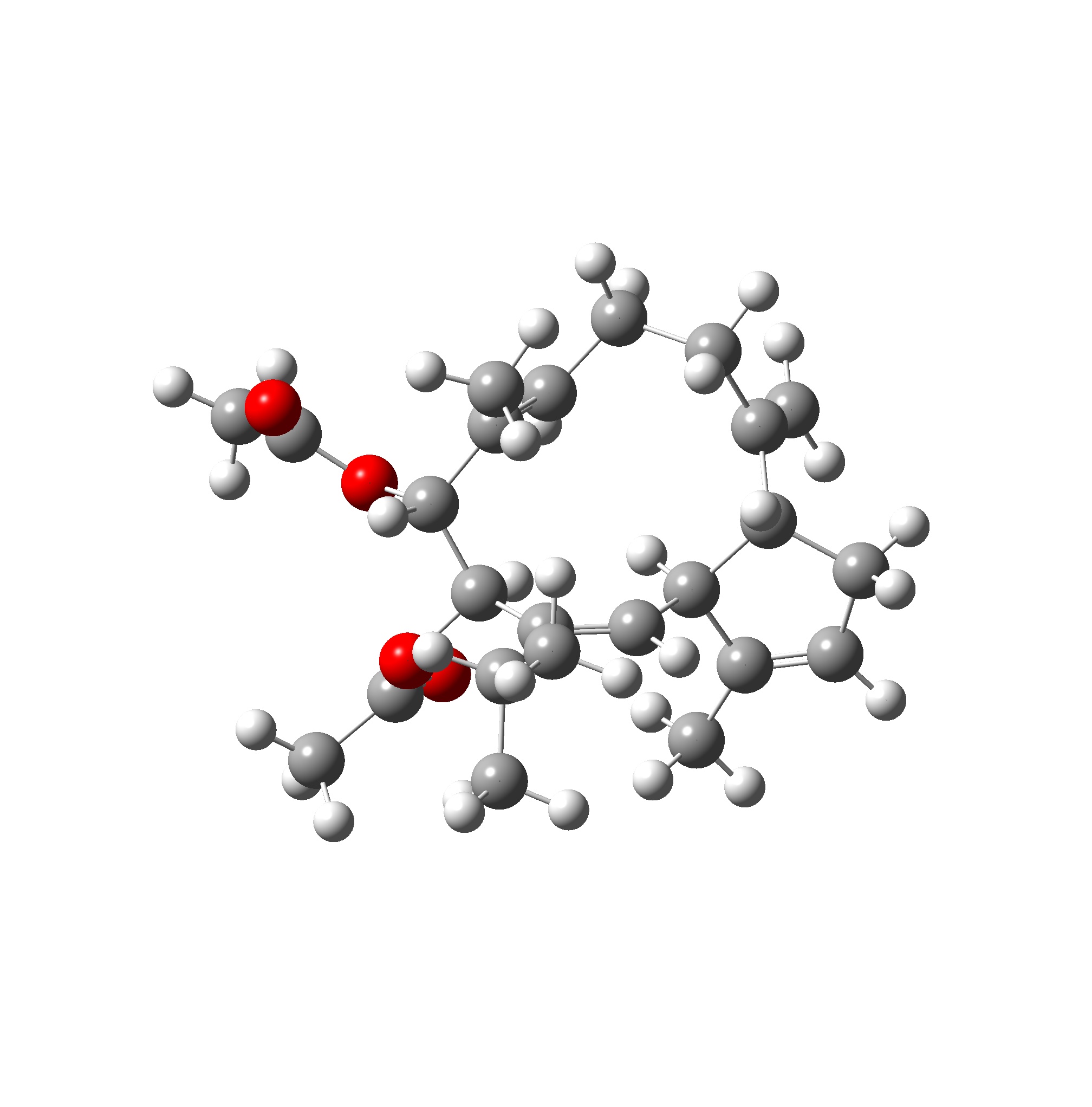

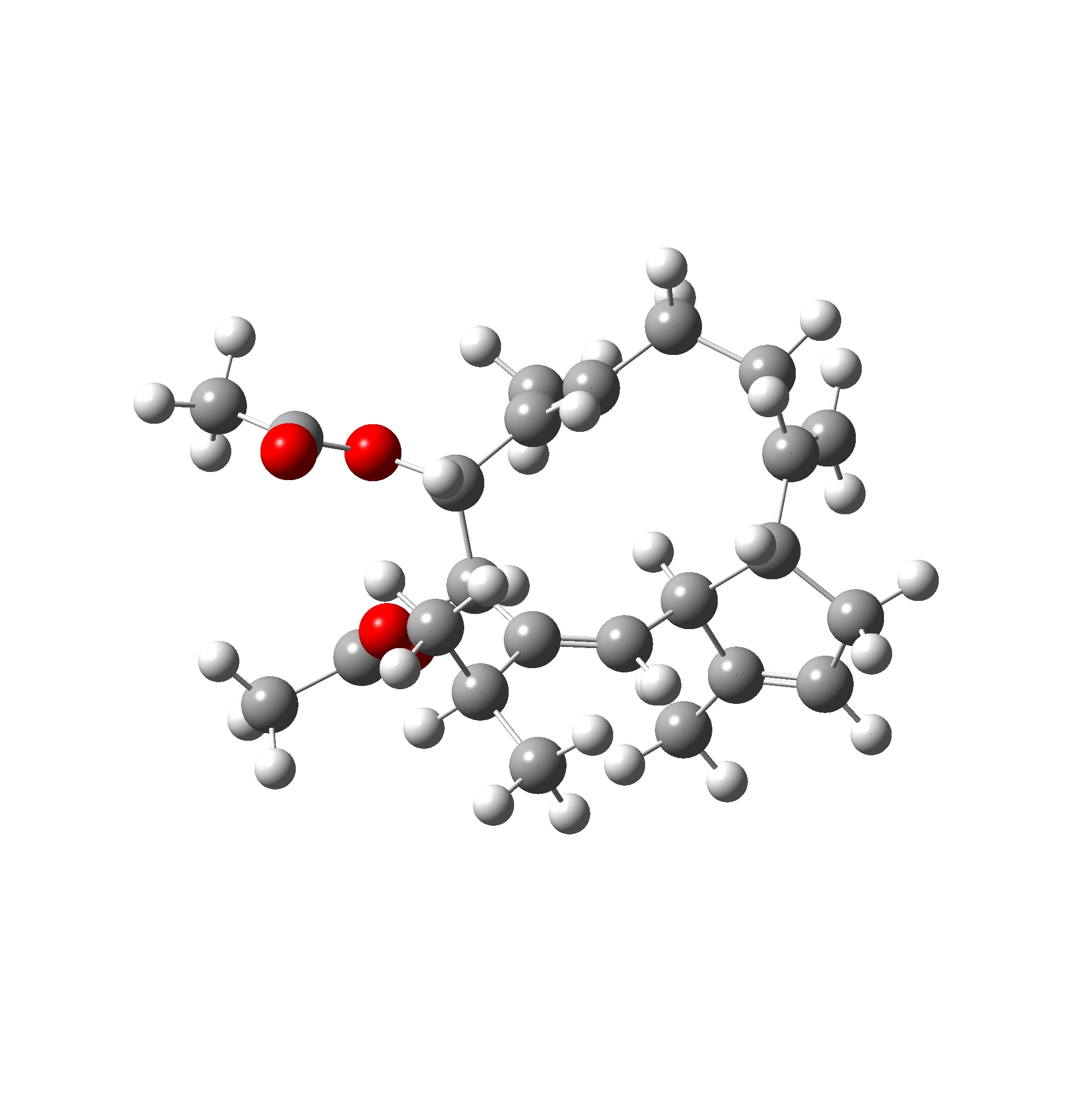

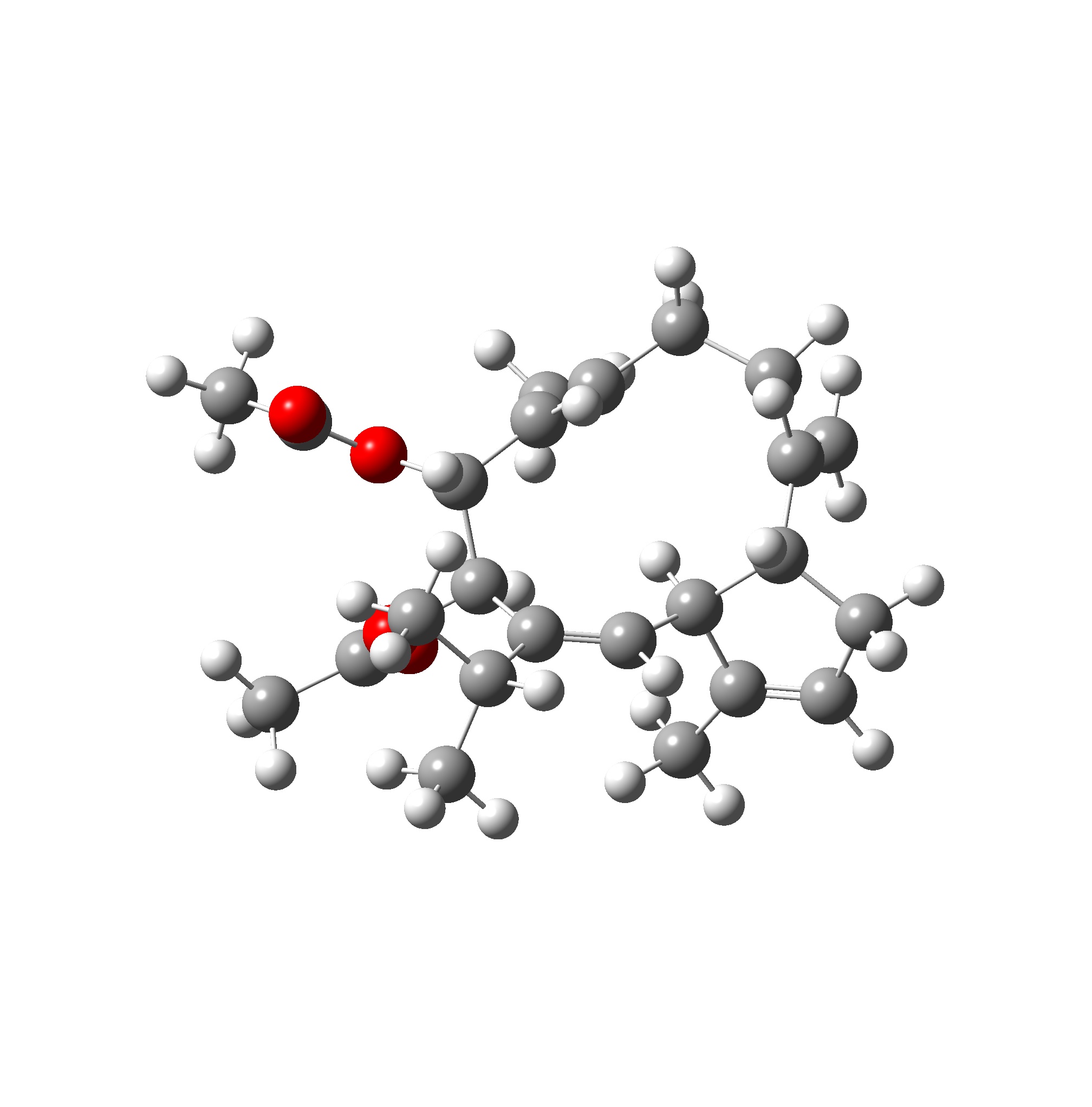


Conf. 5 Conf. 6 Conf. 7 Conf. 8

2.59% 0.58% 0.16% 0.05%

**Figure S3.** Re-optimized conformers above 1% population (OPLS_2005) of (3*S*, 7*R*, *13R*, 14*R*) for **9** calculated at the B3LYP/6-311G (d, p) level with IEFPCM solvent model for acetonitrile.

%nprocshared=n

%mem=n MB

%chk=name.chk

# opt freq b3lyp/6-311g(d,p) scrf(solvent=acetonitrile)

name

0 1

x, y, z coordinates

**Scheme S1.** key words of the input model used in the re-optimization calculations

%nprocshared=n

%mem=nMB

%chk=name.chk

# b3lyp/6-311g(d,p) scrf(solvent=acetonitrile) td(nstates=30,singlets)

name

0 1

x, y, z coordinates

**Scheme S2.** key words of the input file by TDDFT calculation for ECD spectra


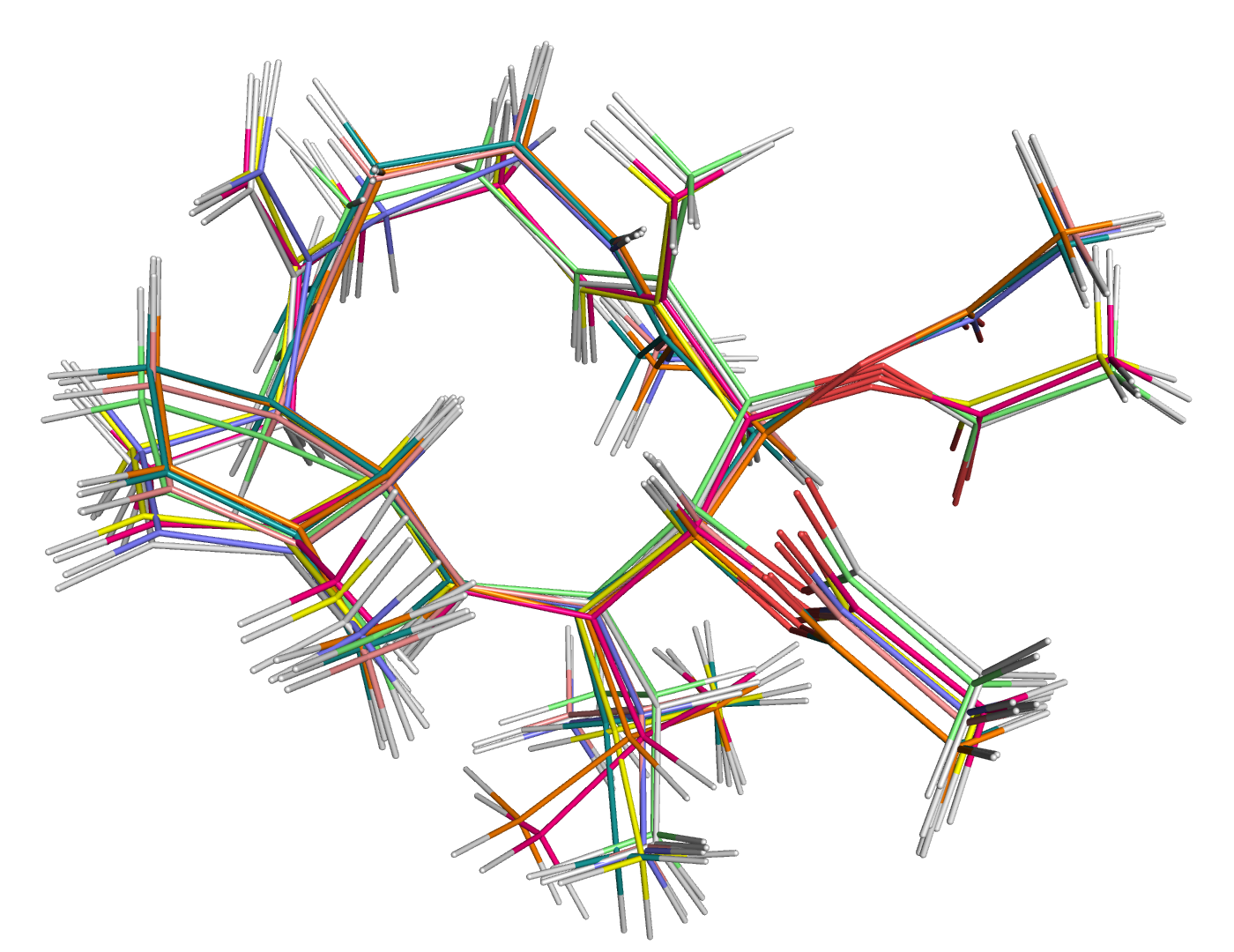


**Figure S4.** Superimposed figure of the re-optimized conformers above 1% population of (3*S*, 7*R*, *13R*, 14*R*) for **9**.

**Table S2.** Comparison of the specific splitting patterns and ^1^H-^1^H coupling constants for the most populated conformer of **9** with experimental ones

| NO. | Calculated data |  | Experimental data |  |
| --- | --- | --- | --- | --- |
|  | mult. (*J*, Hz) |  | mult. (*J*, Hz) |  |
| H-2 | d (11.7) |  | d (10.6) |  |
| H-3 | dd (9.9, 11.7) |  | t (9.5) |  |
| H-5 | dd (5.2, 1.4) |  | t (3.5) |  |
| H-7 | ddd (9.9, 9.9, 7.4) |  | dt (9.5, 8.9) |  |
| H-11 | dd (4.7, 13.0) |  | dd (6.5, 10.1) |  |
| H-13 | d (8.0) |  | d (10.2) |  |
| H-14 | d (8.0) |  | d (10.2) |  |


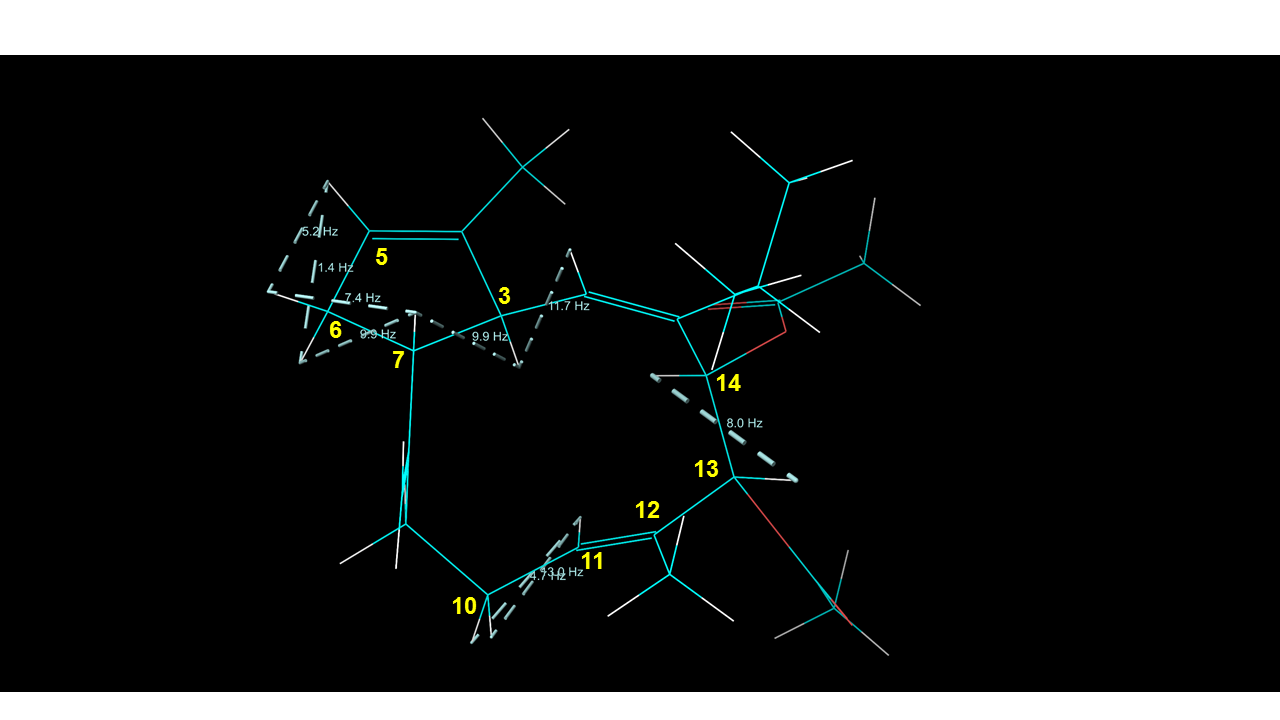


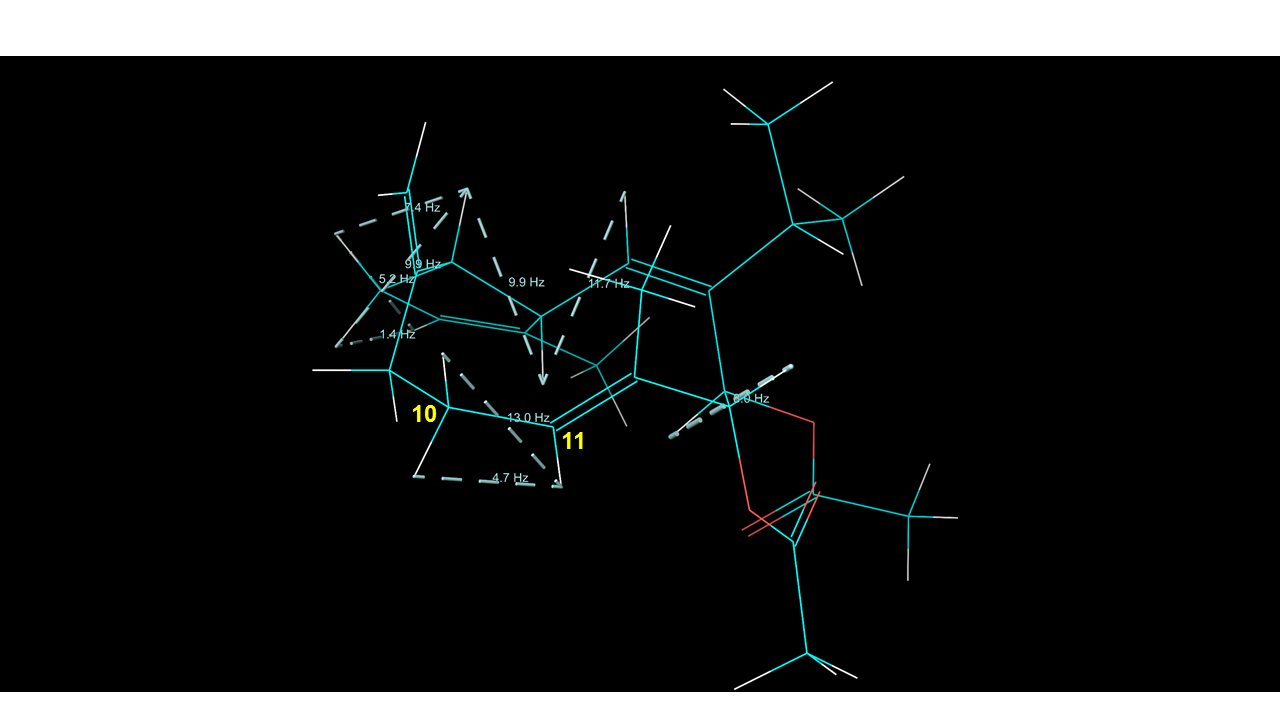


**Figure S5.**  Specific ^1^H-^1^H coupling constants of the most populated conformer (measured in Schrödinger LLC, 2015-4)

**Table S3.** Cartesian coordinates for the re-optimized conformers of compound **9** at the B3LYP/6-311G(d,p) level with IEFPCM solvent model for acetonitrile.

| Compound **9**  Conformer1 | | Standard Orientation  (Ångstroms) | | |
| --- | --- | --- | --- | --- |
| I | atom | X | Y | Z |
| 1 | C | -3.95041300 | 1.54470600 | -1.58201700 |
| 2 | C | -4.40122600 | 0.17565500 | -1.14504100 |
| 3 | C | -3.35057200 | -0.21134300 | -0.06487000 |
| 4 | C | -2.09469500 | 0.66495600 | -0.40463800 |
| 5 | C | -2.70935900 | 1.83438800 | -1.18517600 |
| 6 | C | -1.33449000 | 1.07953800 | 0.82953400 |
| 7 | C | -0.01945600 | 1.04478800 | 1.08525800 |
| 8 | C | 0.98816800 | 0.55049200 | 0.06160900 |
| 9 | C | 0.55254600 | 1.56180900 | 2.41214900 |
| 10 | C | 0.68777600 | 3.09814000 | 2.40193000 |
| 11 | C | -0.24236800 | 1.11463600 | 3.65027700 |
| 12 | C | 1.61321000 | -0.82008400 | 0.38201300 |
| 13 | C | 0.57248100 | -1.93122500 | 0.45032400 |
| 14 | C | -1.92489000 | 3.07091900 | -1.50035900 |
| 15 | C | -3.12589300 | -1.70058400 | 0.09323900 |
| 16 | C | -3.45051700 | -2.31251100 | 1.23567200 |
| 17 | C | -2.58362200 | -2.49874900 | -1.08856100 |
| 18 | C | -1.23756400 | -3.22137700 | -0.81725000 |
| 19 | C | -0.10469900 | -2.24974900 | -0.65816700 |
| 20 | C | 0.40715000 | -2.57083900 | 1.80249900 |
| 21 | O | 2.08921300 | 1.50787100 | -0.00132600 |
| 22 | O | 1.69126400 | 1.96449900 | -2.18312500 |
| 23 | C | 2.34364500 | 2.12220900 | -1.17683900 |
| 24 | C | 3.52878400 | 3.04302800 | -1.05334400 |
| 25 | O | 2.57138600 | -1.05011600 | -0.69235900 |
| 26 | O | 3.75013200 | -2.45853200 | 0.61844900 |
| 27 | C | 3.58911400 | -1.89994000 | -0.44209200 |
| 28 | C | 4.46725200 | -2.06317100 | -1.65412900 |
| 29 | H | -4.56157600 | 2.19444800 | -2.20046200 |
| 30 | H | -4.39766100 | -0.52280100 | -1.99185900 |
| 31 | H | -5.41597300 | 0.15746300 | -0.73484800 |
| 32 | H | -3.73389100 | 0.16485500 | 0.88914400 |
| 33 | H | -1.43556900 | 0.12182300 | -1.08806100 |
| 34 | H | -1.97931900 | 1.47048400 | 1.61545700 |
| 35 | H | 0.54849500 | 0.50240500 | -0.92989400 |
| 36 | H | 1.56309200 | 1.15268200 | 2.51162800 |
| 37 | H | 1.17098400 | 3.44578800 | 3.32021600 |
| 38 | H | 1.28195500 | 3.43967200 | 1.55359300 |
| 39 | H | -0.29837300 | 3.56830100 | 2.33970800 |
| 40 | H | 0.27313400 | 1.43662700 | 4.55961600 |
| 41 | H | -1.24280500 | 1.55523500 | 3.66984700 |
| 42 | H | -0.35387300 | 0.02895200 | 3.69120600 |
| 43 | H | 2.17364200 | -0.77075300 | 1.31594400 |
| 44 | H | -1.64907600 | 3.61023200 | -0.58809600 |
| 45 | H | -0.98633000 | 2.82240200 | -2.00829600 |
| 46 | H | -2.49559500 | 3.74834000 | -2.13925200 |
| 47 | H | -3.33396600 | -3.38402400 | 1.36632600 |
| 48 | H | -3.84714200 | -1.76107700 | 2.08169800 |
| 49 | H | -2.46269500 | -1.84964500 | -1.96049700 |
| 50 | H | -3.32229100 | -3.25418400 | -1.37567500 |
| 51 | H | -1.34132100 | -3.86746300 | 0.05564900 |
| 52 | H | -1.03173700 | -3.87527400 | -1.67287500 |
| 53 | H | 0.15452300 | -1.71760400 | -1.57228200 |
| 54 | H | 0.13550400 | -1.82808900 | 2.55870300 |
| 55 | H | -0.35807600 | -3.34529000 | 1.80970100 |
| 56 | H | 1.35804400 | -3.01261600 | 2.11804400 |
| 57 | H | 3.23820600 | 3.92792100 | -0.48074500 |
| 58 | H | 4.34221500 | 2.55261000 | -0.51776100 |
| 59 | H | 3.85588800 | 3.35152700 | -2.04414900 |
| 60 | H | 4.69617700 | -1.09351400 | -2.09776700 |
| 61 | H | 5.38329000 | -2.57973900 | -1.37518200 |
| 62 | H | 3.93397400 | -2.65361300 | -2.40400700 |

B3LYP/6-311G(d,p) Energy = -1236.2288178a.u.; Population =73.79% .

| Compound **9**  Conformer 2 | | Standard Orientation  (Ångstroms) | | |
| --- | --- | --- | --- | --- |
| I | atom | X | Y | Z |
| 1 | C | 3.85185800 | -1.99949200 | -1.34071600 |
| 2 | C | 4.49665900 | -0.72819800 | -0.85135500 |
| 3 | C | 3.48117900 | -0.21540400 | 0.20776200 |
| 4 | C | 2.11268400 | -0.83298600 | -0.24162500 |
| 5 | C | 2.56080900 | -2.08337100 | -1.01116400 |
| 6 | C | 1.19321600 | -1.08668300 | 0.92541300 |
| 7 | C | -0.12250000 | -0.86294300 | 1.03914100 |
| 8 | C | -0.95926400 | -0.32341300 | -0.10509800 |
| 9 | C | -0.87794700 | -1.15929600 | 2.34080600 |
| 10 | C | -1.13318100 | -2.66973900 | 2.50920000 |
| 11 | C | -0.18819100 | -0.58416300 | 3.58959600 |
| 12 | C | -1.31145700 | 1.19299100 | 0.07699500 |
| 13 | C | -0.25791000 | 2.04978000 | -0.59112100 |
| 14 | C | 1.59834700 | -3.16528700 | -1.39358200 |
| 15 | C | 3.51446400 | 1.27712700 | 0.45932500 |
| 16 | C | 3.91413200 | 1.75168800 | 1.64258200 |
| 17 | C | 3.12645300 | 2.21742100 | -0.67618900 |
| 18 | C | 1.99161700 | 3.21704600 | -0.32968700 |
| 19 | C | 0.74378100 | 2.52707100 | 0.15694800 |
| 20 | C | -0.37832600 | 2.21386600 | -2.08576300 |
| 21 | O | -2.18673200 | -1.10044000 | -0.16256700 |
| 22 | O | -1.94061300 | -1.47215200 | -2.38277500 |
| 23 | C | -2.56134700 | -1.61704400 | -1.35505600 |
| 24 | C | -3.82936500 | -2.41660900 | -1.21707800 |
| 25 | O | -2.59324700 | 1.47696600 | -0.53739700 |
| 26 | O | -3.65362200 | 1.41862300 | 1.46085600 |
| 27 | C | -3.68162000 | 1.56156000 | 0.26104100 |
| 28 | C | -4.91211800 | 1.87081400 | -0.54927600 |
| 29 | H | 4.37895200 | -2.73241800 | -1.94320200 |
| 30 | H | 4.62422100 | -0.01489500 | -1.67632700 |
| 31 | H | 5.48837400 | -0.87623700 | -0.41262000 |
| 32 | H | 3.74102100 | -0.71219600 | 1.14840300 |
| 33 | H | 1.62043500 | -0.16574600 | -0.95661000 |
| 34 | H | 1.70124200 | -1.49722500 | 1.79720700 |
| 35 | H | -0.45912800 | -0.45577100 | -1.05896600 |
| 36 | H | -1.85543800 | -0.67533100 | 2.26228200 |
| 37 | H | -1.74099700 | -2.85962100 | 3.39931500 |
| 38 | H | -1.65800700 | -3.07949400 | 1.64415500 |
| 39 | H | -0.18929300 | -3.21151200 | 2.62293700 |
| 40 | H | -0.81114300 | -0.75226700 | 4.47313000 |
| 41 | H | 0.77896300 | -1.05973300 | 3.77468500 |
| 42 | H | -0.01999000 | 0.49145000 | 3.49356300 |
| 43 | H | -1.39110300 | 1.42341200 | 1.13709000 |
| 44 | H | 1.15507200 | -3.63566900 | -0.50987100 |
| 45 | H | 0.76520500 | -2.76239400 | -1.98111700 |
| 46 | H | 2.09000200 | -3.94036400 | -1.98541500 |
| 47 | H | 3.97564700 | 2.81639800 | 1.84527400 |
| 48 | H | 4.19893500 | 1.08702900 | 2.45160700 |
| 49 | H | 2.81846200 | 1.63667000 | -1.54922400 |
| 50 | H | 4.00622700 | 2.79034000 | -0.98912200 |
| 51 | H | 2.34419500 | 3.90513500 | 0.44373400 |
| 52 | H | 1.78923000 | 3.82471300 | -1.21542200 |
| 53 | H | 0.70573500 | 2.33681400 | 1.22607000 |
| 54 | H | -1.26541000 | 2.80350000 | -2.33359900 |
| 55 | H | 0.49190800 | 2.70771400 | -2.51570400 |
| 56 | H | -0.50099000 | 1.24770400 | -2.58571600 |
| 57 | H | -4.54878400 | -1.89879400 | -0.58248500 |
| 58 | H | -4.25241100 | -2.60228000 | -2.20221000 |
| 59 | H | -3.59514300 | -3.37367500 | -0.74289400 |
| 60 | H | -4.77777900 | 2.81581800 | -1.07987000 |
| 61 | H | -5.06979800 | 1.09244700 | -1.29856200 |
| 62 | H | -5.77653900 | 1.93409200 | 0.10795200 |

B3LYP/6-311G(d,p) Energy =-1236.2273869a.u.; Population =16.19% .

| Compound **9**  Conformer 3 | | Standard Orientation  (Ångstroms) | | |
| --- | --- | --- | --- | --- |
| I | atom | X | Y | Z |
| 1 | C | 3.89056000 | -1.82090400 | -1.44718300 |
| 2 | C | 4.46611100 | -0.52474300 | -0.94267200 |
| 3 | C | 3.41946600 | -0.05987800 | 0.11099000 |
| 4 | C | 2.09376000 | -0.79590300 | -0.29203500 |
| 5 | C | 2.61248400 | -1.99222100 | -1.10310100 |
| 6 | C | 1.26624900 | -1.19283900 | 0.90486200 |
| 7 | C | -0.05635300 | -1.11403000 | 1.12292900 |
| 8 | C | -0.98925800 | -0.50772600 | 0.08541200 |
| 9 | C | -0.61272800 | -1.73394200 | 2.41462200 |
| 10 | C | -1.75202500 | -0.96072300 | 3.09965800 |
| 11 | C | -1.02172800 | -3.20866200 | 2.21146800 |
| 12 | C | -1.46148200 | 0.93705300 | 0.35626700 |
| 13 | C | -0.30913700 | 1.92049800 | 0.51568500 |
| 14 | C | 1.71944400 | -3.12936900 | -1.49517200 |
| 15 | C | 3.34808200 | 1.44110900 | 0.29544600 |
| 16 | C | 3.71073800 | 1.99347000 | 1.45660400 |
| 17 | C | 2.92164700 | 2.31533200 | -0.87915600 |
| 18 | C | 1.62660700 | 3.13398100 | -0.63381500 |
| 19 | C | 0.40869300 | 2.25909800 | -0.56067300 |
| 20 | C | -0.08471600 | 2.41979300 | 1.91794900 |
| 21 | O | -2.19392300 | -1.32280100 | -0.02186800 |
| 22 | O | -1.76796900 | -1.84004900 | -2.18536200 |
| 23 | C | -2.48051400 | -1.89971400 | -1.21028000 |
| 24 | C | -3.79509100 | -2.63074300 | -1.14354600 |
| 25 | O | -2.28187400 | 1.26590600 | -0.80241700 |
| 26 | O | -3.43324800 | 2.78442200 | 0.40679100 |
| 27 | C | -3.22398600 | 2.21901300 | -0.64142600 |
| 28 | C | -3.94854800 | 2.48670700 | -1.93369200 |
| 29 | H | 4.45193300 | -2.50883600 | -2.07158000 |
| 30 | H | 4.57388500 | 0.19722700 | -1.76247300 |
| 31 | H | 5.45870500 | -0.63018900 | -0.49319600 |
| 32 | H | 3.72867700 | -0.49224400 | 1.06792200 |
| 33 | H | 1.50539000 | -0.16559800 | -0.96443600 |
| 34 | H | 1.85684100 | -1.65168600 | 1.69862000 |
| 35 | H | -0.51413400 | -0.51564700 | -0.89205900 |
| 36 | H | 0.22964500 | -1.74103500 | 3.11439500 |
| 37 | H | -2.00136100 | -1.44806000 | 4.04643700 |
| 38 | H | -1.47044800 | 0.06946000 | 3.32835500 |
| 39 | H | -2.65645400 | -0.94755200 | 2.48792500 |
| 40 | H | -1.27713000 | -3.66142900 | 3.17470600 |
| 41 | H | -1.89116400 | -3.29438000 | 1.55772100 |
| 42 | H | -0.20408600 | -3.78553900 | 1.77287900 |
| 43 | H | -2.10548300 | 0.97045800 | 1.23245100 |
| 44 | H | 1.36036900 | -3.67505300 | -0.61636700 |
| 45 | H | 0.82776400 | -2.76896700 | -2.01982100 |
| 46 | H | 2.24251600 | -3.83423100 | -2.14511500 |
| 47 | H | 3.70909600 | 3.06887300 | 1.60596700 |
| 48 | H | 4.02402700 | 1.38679700 | 2.29988000 |
| 49 | H | 2.78624200 | 1.70623000 | -1.77750800 |
| 50 | H | 3.72803300 | 3.01898000 | -1.10961700 |
| 51 | H | 1.74557000 | 3.73729500 | 0.26768400 |
| 52 | H | 1.51169800 | 3.83403300 | -1.46950500 |
| 53 | H | 0.10860700 | 1.83003200 | -1.51520400 |
| 54 | H | 0.16853200 | 1.59668900 | 2.59333900 |
| 55 | H | 0.72062900 | 3.15006600 | 1.97573100 |
| 56 | H | -1.00170000 | 2.87996700 | 2.30126800 |
| 57 | H | -3.72876900 | -3.44416900 | -0.41733000 |
| 58 | H | -4.58290300 | -1.95341700 | -0.80893500 |
| 59 | H | -4.03893900 | -3.03446300 | -2.12377700 |
| 60 | H | -4.85132800 | 3.06053900 | -1.73451400 |
| 61 | H | -3.29512900 | 3.06433400 | -2.59333700 |
| 62 | H | -4.19265400 | 1.55372900 | -2.44249100 |

B3LYP/6-311G(d,p) Energy = -1236.2260797a.u.; Population = 4.05% .

| Compound **9**  Conformer 4 | | Standard Orientation  (Ångstroms) | | |
| --- | --- | --- | --- | --- |
| I | atom | X | Y | Z |
| 1 | C | 4.08096000 | -2.00158200 | -0.63712100 |
| 2 | C | 4.55993700 | -0.89773500 | 0.26384000 |
| 3 | C | 3.31478600 | 0.03461600 | 0.44273800 |
| 4 | C | 2.10942000 | -0.76019700 | -0.20758100 |
| 5 | C | 2.77729000 | -1.93721900 | -0.91162200 |
| 6 | C | 1.13222200 | -1.22780500 | 0.84431400 |
| 7 | C | -0.19537000 | -1.07033900 | 0.91416500 |
| 8 | C | -0.98307200 | -0.34883900 | -0.15983900 |
| 9 | C | -1.00751300 | -1.60622200 | 2.10011700 |
| 10 | C | -1.23227900 | -3.12581800 | 1.97897300 |
| 11 | C | -0.39783500 | -1.25563400 | 3.46781300 |
| 12 | C | -1.34371800 | 1.11051500 | 0.27689200 |
| 13 | C | -0.26644400 | 2.07821100 | -0.16807100 |
| 14 | C | 1.98530500 | -2.86503800 | -1.78184800 |
| 15 | C | 3.50917300 | 1.43173400 | -0.13966400 |
| 16 | C | 3.94100800 | 1.61625600 | -1.39053200 |
| 17 | C | 3.21230600 | 2.62646900 | 0.75757100 |
| 18 | C | 1.86003900 | 3.33423600 | 0.48028600 |
| 19 | C | 0.65788800 | 2.46664800 | 0.71913100 |
| 20 | C | -0.31270600 | 2.48476600 | -1.62091000 |
| 21 | O | -2.20278900 | -1.09958600 | -0.40543700 |
| 22 | O | -1.89198500 | -1.02138100 | -2.64708300 |
| 23 | C | -2.54015900 | -1.36896400 | -1.68742600 |
| 24 | C | -3.80662400 | -2.18078400 | -1.74710900 |
| 25 | O | -2.59449000 | 1.51166600 | -0.33945800 |
| 26 | O | -3.75245800 | 1.13900200 | 1.56922800 |
| 27 | C | -3.71990500 | 1.47810000 | 0.40953900 |
| 28 | C | -4.90712400 | 1.93437200 | -0.39635900 |
| 29 | H | 4.74765000 | -2.76892600 | -1.01871000 |
| 30 | H | 5.40603800 | -0.35339800 | -0.16648700 |
| 31 | H | 4.90487900 | -1.28916900 | 1.22858200 |
| 32 | H | 3.11155900 | 0.14956600 | 1.50817000 |
| 33 | H | 1.60654200 | -0.12489600 | -0.93921400 |
| 34 | H | 1.61351300 | -1.75684900 | 1.66607900 |
| 35 | H | -0.43944800 | -0.31301500 | -1.09860300 |
| 36 | H | -1.99153200 | -1.13100100 | 2.05650900 |
| 37 | H | -1.87759700 | -3.48531200 | 2.78652200 |
| 38 | H | -1.70327700 | -3.37901100 | 1.02726200 |
| 39 | H | -0.28080300 | -3.66271300 | 2.04127300 |
| 40 | H | -1.05928400 | -1.59362600 | 4.27103200 |
| 41 | H | 0.57210000 | -1.73817100 | 3.61715000 |
| 42 | H | -0.25705500 | -0.17723200 | 3.57746000 |
| 43 | H | -1.47920100 | 1.14333100 | 1.35584600 |
| 44 | H | 1.18175500 | -3.34864700 | -1.21634300 |
| 45 | H | 1.50424300 | -2.31791500 | -2.60085400 |
| 46 | H | 2.61657200 | -3.64388800 | -2.21462700 |
| 47 | H | 4.09087600 | 2.61127700 | -1.79790200 |
| 48 | H | 4.15823900 | 0.78387400 | -2.05208500 |
| 49 | H | 4.00137500 | 3.37411800 | 0.63140200 |
| 50 | H | 3.24185400 | 2.31503600 | 1.80641900 |
| 51 | H | 1.80343800 | 4.20218600 | 1.14970300 |
| 52 | H | 1.87104200 | 3.72798000 | -0.53734400 |
| 53 | H | 0.55523800 | 2.10445400 | 1.74098600 |
| 54 | H | -1.15279300 | 3.16230900 | -1.79969600 |
| 55 | H | 0.60053000 | 2.98320600 | -1.94089100 |
| 56 | H | -0.47238900 | 1.61892000 | -2.27042400 |
| 57 | H | -3.58852700 | -3.20501000 | -1.43286300 |
| 58 | H | -4.55753200 | -1.77824700 | -1.06668800 |
| 59 | H | -4.18428900 | -2.19309900 | -2.76740800 |
| 60 | H | -5.80487100 | 1.88786000 | 0.21609900 |
| 61 | H | -4.74635500 | 2.95744400 | -0.74316600 |
| 62 | H | -5.02422300 | 1.30249500 | -1.27896400 |

B3LYP/6-311G(d,p) Energy = -1236.2256607a.u.; Population = 2.60% .

| Compound **9**  Conformer 5 | | Standard Orientation  (Ångstroms) | | |
| --- | --- | --- | --- | --- |
| I | atom | X | Y | Z |
| 1 | C | 3.83790500 | -1.54716800 | -1.72276500 |
| 2 | C | 4.37548800 | -0.26097600 | -1.15500800 |
| 3 | C | 3.34486400 | 0.09431200 | -0.04400400 |
| 4 | C | 2.04009400 | -0.68063300 | -0.44316300 |
| 5 | C | 2.58221000 | -1.79833400 | -1.34695300 |
| 6 | C | 1.28830800 | -1.20944000 | 0.75417300 |
| 7 | C | -0.02624300 | -1.21894100 | 1.02611100 |
| 8 | C | -1.02717700 | -0.58872000 | 0.05853600 |
| 9 | C | -0.59626200 | -1.98266500 | 2.23000100 |
| 10 | C | 0.44361700 | -2.79833700 | 3.00906500 |
| 11 | C | -1.40069300 | -1.10861700 | 3.21329900 |
| 12 | C | -1.54316600 | 0.82997100 | 0.38684000 |
| 13 | C | -0.43631200 | 1.86083000 | 0.56065000 |
| 14 | C | 1.72827100 | -2.95164300 | -1.77756200 |
| 15 | C | 3.21348800 | 1.57863600 | 0.22271500 |
| 16 | C | 3.58501700 | 2.08646200 | 1.40128200 |
| 17 | C | 2.71931100 | 2.49249100 | -0.89398100 |
| 18 | C | 1.39444500 | 3.23428600 | -0.57713900 |
| 19 | C | 0.22439200 | 2.29555800 | -0.51757500 |
| 20 | C | -0.18697800 | 2.29747500 | 1.97939500 |
| 21 | O | -2.20946700 | -1.43803700 | -0.01732700 |
| 22 | O | -1.81892000 | -1.96545100 | -2.18432200 |
| 23 | C | -2.49739300 | -2.04970500 | -1.18701400 |
| 24 | C | -3.76513100 | -2.85305500 | -1.06749600 |
| 25 | O | -2.39832900 | 1.15947800 | -0.74686800 |
| 26 | O | -3.60381100 | 2.57523800 | 0.53251600 |
| 27 | C | -3.38357800 | 2.05813700 | -0.53811600 |
| 28 | C | -4.14165900 | 2.33371200 | -1.80938000 |
| 29 | H | 4.40582100 | -2.16875700 | -2.40791100 |
| 30 | H | 4.42840400 | 0.51382400 | -1.93050600 |
| 31 | H | 5.38525400 | -0.35202400 | -0.74203200 |
| 32 | H | 3.70722900 | -0.37260000 | 0.87734500 |
| 33 | H | 1.39429700 | -0.04257400 | -1.05114200 |
| 34 | H | 1.94331100 | -1.70529300 | 1.46517600 |
| 35 | H | -0.59835300 | -0.55030200 | -0.93965700 |
| 36 | H | -1.30808300 | -2.69968800 | 1.80553300 |
| 37 | H | -0.06019000 | -3.37844000 | 3.78642900 |
| 38 | H | 0.98129400 | -3.49918800 | 2.36643000 |
| 39 | H | 1.17870700 | -2.15558800 | 3.50346100 |
| 40 | H | -1.80139500 | -1.73389600 | 4.01599800 |
| 41 | H | -0.76975000 | -0.34242400 | 3.67214100 |
| 42 | H | -2.24873600 | -0.61720700 | 2.73553300 |
| 43 | H | -2.17308200 | 0.81135900 | 1.27336900 |
| 44 | H | 1.43133900 | -3.56775700 | -0.92218700 |
| 45 | H | 0.79938200 | -2.60532000 | -2.24353000 |
| 46 | H | 2.25796100 | -3.58813100 | -2.48977000 |
| 47 | H | 3.54212100 | 3.15180000 | 1.60643100 |
| 48 | H | 3.94759600 | 1.45234500 | 2.20360100 |
| 49 | H | 2.59046900 | 1.92543200 | -1.82036400 |
| 50 | H | 3.48682800 | 3.24441300 | -1.10387700 |
| 51 | H | 1.50996500 | 3.79667200 | 0.35082300 |
| 52 | H | 1.22350700 | 3.96810500 | -1.37325000 |
| 53 | H | -0.08420500 | 1.90213500 | -1.48465900 |
| 54 | H | 0.16359300 | 1.46138400 | 2.59131400 |
| 55 | H | 0.55874000 | 3.08781200 | 2.04535600 |
| 56 | H | -1.11806800 | 2.65808600 | 2.42917300 |
| 57 | H | -3.62085200 | -3.65847100 | -0.34345300 |
| 58 | H | -4.57639800 | -2.22152900 | -0.70118500 |
| 59 | H | -4.02474700 | -3.27387500 | -2.03641200 |
| 60 | H | -4.34567400 | 1.40796800 | -2.34816000 |
| 61 | H | -5.06942200 | 2.85244400 | -1.57663500 |
| 62 | H | -3.52956900 | 2.96764900 | -2.45682300 |

B3LYP/6-311G(d,p) Energy = -1236.2256592 a.u.; Population = 2.59% .

| Compound **9**  Conformer 6 | | Standard Orientation  (Ångstroms) | | |
| --- | --- | --- | --- | --- |
| I | atom | X | Y | Z |
| 1 | C | 4.22428500 | -1.46871600 | -0.87352900 |
| 2 | C | 4.60316200 | -0.25034400 | -0.08055500 |
| 3 | C | 3.25074500 | 0.48086400 | 0.19429800 |
| 4 | C | 2.12697100 | -0.52879000 | -0.29059200 |
| 5 | C | 2.90689100 | -1.62956800 | -1.00503100 |
| 6 | C | 1.32372500 | -1.08506500 | 0.86043400 |
| 7 | C | -0.00335100 | -1.13021600 | 1.04218400 |
| 8 | C | -0.97353600 | -0.57910600 | 0.01376700 |
| 9 | C | -0.61932800 | -1.79256500 | 2.28184800 |
| 10 | C | -0.67924600 | -3.32525200 | 2.12082000 |
| 11 | C | 0.08796600 | -1.42910200 | 3.59791100 |
| 12 | C | -1.68608200 | 0.71898900 | 0.43551900 |
| 13 | C | -0.73062000 | 1.87317100 | 0.70790700 |
| 14 | C | 2.19709600 | -2.72651800 | -1.73893700 |
| 15 | C | 3.12089100 | 1.85152500 | -0.46274200 |
| 16 | C | 3.57932500 | 2.09710300 | -1.69321500 |
| 17 | C | 2.44262500 | 2.95596000 | 0.34372100 |
| 18 | C | 1.06801000 | 3.43533800 | -0.19135800 |
| 19 | C | 0.03572000 | 2.34128800 | -0.28371200 |
| 20 | C | -0.73355000 | 2.40100900 | 2.11804000 |
| 21 | O | -2.02339900 | -1.56935800 | -0.21085700 |
| 22 | O | -1.51024700 | -1.76117200 | -2.40825500 |
| 23 | C | -2.19886800 | -2.05589000 | -1.45909500 |
| 24 | C | -3.35366900 | -3.02187200 | -1.49542300 |
| 25 | O | -2.57823800 | 1.02777300 | -0.67723500 |
| 26 | O | -3.98673400 | 2.12642100 | 0.70234300 |
| 27 | C | -3.69009400 | 1.74194700 | -0.40540400 |
| 28 | C | -4.48420500 | 2.00039900 | -1.65829200 |
| 29 | H | 4.96508300 | -2.14294500 | -1.29246500 |
| 30 | H | 5.30592300 | 0.39196000 | -0.61966200 |
| 31 | H | 5.10033100 | -0.52278200 | 0.85837300 |
| 32 | H | 3.13951400 | 0.62348700 | 1.27046800 |
| 33 | H | 1.47197300 | -0.02617800 | -1.00402800 |
| 34 | H | 1.94693900 | -1.51445600 | 1.64358200 |
| 35 | H | -0.48233100 | -0.40904200 | -0.93953300 |
| 36 | H | -1.65224700 | -1.43901700 | 2.36158200 |
| 37 | H | -1.19240600 | -3.78105500 | 2.97322400 |
| 38 | H | -1.21075600 | -3.60753100 | 1.21122400 |
| 39 | H | 0.33051200 | -3.74371400 | 2.06965700 |
| 40 | H | -0.45224600 | -1.86376700 | 4.44375700 |
| 41 | H | 1.10942100 | -1.81777900 | 3.62904700 |
| 42 | H | 0.13564800 | -0.34815000 | 3.74786600 |
| 43 | H | -2.30844700 | 0.53520400 | 1.31138100 |
| 44 | H | 1.56206000 | -3.30837500 | -1.06245400 |
| 45 | H | 1.53489800 | -2.31927500 | -2.51087600 |
| 46 | H | 2.90421500 | -3.40837700 | -2.21597000 |
| 47 | H | 3.47542000 | 3.07611200 | -2.15090000 |
| 48 | H | 4.06928200 | 1.33636500 | -2.29133500 |
| 49 | H | 3.10529400 | 3.82751000 | 0.37611400 |
| 50 | H | 2.31488500 | 2.62530600 | 1.37743000 |
| 51 | H | 0.71940800 | 4.24210700 | 0.46072600 |
| 52 | H | 1.21022600 | 3.87605000 | -1.18190500 |
| 53 | H | -0.06764700 | 1.87970700 | -1.26225500 |
| 54 | H | -1.75411500 | 2.65447100 | 2.42094200 |
| 55 | H | -0.37004200 | 1.64500100 | 2.82198200 |
| 56 | H | -0.10789400 | 3.28615700 | 2.23224200 |
| 57 | H | -3.10012600 | -3.91592800 | -0.92038100 |
| 58 | H | -4.23605700 | -2.57321800 | -1.03659100 |
| 59 | H | -3.56403000 | -3.30147000 | -2.52551400 |
| 60 | H | -5.44783300 | 2.43312300 | -1.39804200 |
| 61 | H | -3.93294900 | 2.69705600 | -2.29514600 |
| 62 | H | -4.62212700 | 1.07659900 | -2.22187700 |

B3LYP/6-311G(d,p) Energy =-1236.2242436a.u.; Population =0.58% .

| Compound **9**  Conformer 7 | | Standard Orientation  (Ångstroms) | | |
| --- | --- | --- | --- | --- |
| I | atom | X | Y | Z |
| 1 | C | 4.04904600 | -1.89717300 | -0.75386900 |
| 2 | C | 4.49484500 | -0.93304900 | 0.31121200 |
| 3 | C | 3.28199300 | 0.03842000 | 0.48837800 |
| 4 | C | 2.08034700 | -0.68430100 | -0.24326900 |
| 5 | C | 2.76165300 | -1.76616600 | -1.07840300 |
| 6 | C | 1.13832600 | -1.31021000 | 0.75915100 |
| 7 | C | -0.19713600 | -1.24949400 | 0.85867200 |
| 8 | C | -1.02894500 | -0.46227500 | -0.15216500 |
| 9 | C | -0.95483000 | -2.00807600 | 1.95515700 |
| 10 | C | -0.11769100 | -3.07588100 | 2.67257300 |
| 11 | C | -1.60393700 | -1.07286100 | 2.99806500 |
| 12 | C | -1.30971700 | 1.02235600 | 0.25579400 |
| 13 | C | -0.18880800 | 1.95815500 | -0.14913900 |
| 14 | C | 1.99734200 | -2.55762400 | -2.09540000 |
| 15 | C | 3.55292200 | 1.44857700 | -0.02743800 |
| 16 | C | 4.08548000 | 1.67444000 | -1.23192100 |
| 17 | C | 3.21275600 | 2.61321700 | 0.89166900 |
| 18 | C | 1.84906300 | 3.29349900 | 0.60352800 |
| 19 | C | 0.65875100 | 2.39704200 | 0.78974300 |
| 20 | C | -0.13250700 | 2.32415500 | -1.61319900 |
| 21 | O | -2.30242300 | -1.13522300 | -0.32485900 |
| 22 | O | -2.00366700 | -1.31896700 | -2.56188700 |
| 23 | C | -2.67185000 | -1.50671300 | -1.57245500 |
| 24 | C | -4.00973500 | -2.19588400 | -1.54588500 |
| 25 | O | -2.51209500 | 1.46343900 | -0.43542500 |
| 26 | O | -3.73188900 | 1.47685100 | 1.47125200 |
| 27 | C | -3.64215100 | 1.65506000 | 0.27874300 |
| 28 | C | -4.75673200 | 2.14036600 | -0.61016400 |
| 29 | H | 4.71979700 | -2.62728200 | -1.19637800 |
| 30 | H | 5.40301500 | -0.39124700 | 0.03154600 |
| 31 | H | 4.72431900 | -1.45565400 | 1.24826700 |
| 32 | H | 3.04076700 | 0.12063600 | 1.54904700 |
| 33 | H | 1.55522400 | 0.02214600 | -0.88642200 |
| 34 | H | 1.66386700 | -1.90779000 | 1.49886600 |
| 35 | H | -0.53811000 | -0.46856500 | -1.12162400 |
| 36 | H | -1.77224500 | -2.53292200 | 1.45137300 |
| 37 | H | -0.76226100 | -3.64921800 | 3.34395700 |
| 38 | H | 0.34186600 | -3.77480800 | 1.96924200 |
| 39 | H | 0.67694700 | -2.63512300 | 3.28232700 |
| 40 | H | -2.11780500 | -1.66843500 | 3.75852900 |
| 41 | H | -0.84334200 | -0.46913600 | 3.50388100 |
| 42 | H | -2.34036400 | -0.40038200 | 2.55688400 |
| 43 | H | -1.49339400 | 1.08682500 | 1.32367800 |
| 44 | H | 1.14076500 | -3.06667600 | -1.64071000 |
| 45 | H | 1.59320200 | -1.90461300 | -2.87759700 |
| 46 | H | 2.62919200 | -3.30995300 | -2.57205900 |
| 47 | H | 4.28549300 | 2.68303100 | -1.58055100 |
| 48 | H | 4.33872900 | 0.86721100 | -1.91113700 |
| 49 | H | 3.98478700 | 3.38260800 | 0.79612500 |
| 50 | H | 3.23125600 | 2.27725300 | 1.93348600 |
| 51 | H | 1.75480300 | 4.14219300 | 1.29249800 |
| 52 | H | 1.87484300 | 3.71244200 | -0.40364100 |
| 53 | H | 0.49213600 | 2.06272600 | 1.81265300 |
| 54 | H | -0.95085200 | 3.00349100 | -1.86856600 |
| 55 | H | 0.80650000 | 2.80347000 | -1.88360300 |
| 56 | H | -0.25499000 | 1.44315400 | -2.25030200 |
| 57 | H | -3.93088700 | -3.12313300 | -0.97338400 |
| 58 | H | -4.75010700 | -1.56546300 | -1.05052300 |
| 59 | H | -4.32655900 | -2.41881400 | -2.56231400 |
| 60 | H | -4.51424700 | 3.13797900 | -0.98439600 |
| 61 | H | -4.86761700 | 1.48264200 | -1.47365200 |
| 62 | H | -5.68505000 | 2.17909900 | -0.04441900 |

B3LYP/6-311G(d, p) Energy =-1236.2230105a.u.; Population =0.16% .

| Compound **9**  Conformer 8 | | Standard Orientation  (Ångstroms) | | |
| --- | --- | --- | --- | --- |
| I | atom | X | Y | Z |
| 1 | C | 4.08468200 | -2.05896800 | -0.59642200 |
| 2 | C | 4.59487700 | -0.99086400 | 0.32972400 |
| 3 | C | 3.37901800 | -0.02580800 | 0.52809500 |
| 4 | C | 2.14766200 | -0.77713700 | -0.12608400 |
| 5 | C | 2.78232300 | -1.95325900 | -0.86438400 |
| 6 | C | 1.18546100 | -1.25819500 | 0.93328500 |
| 7 | C | -0.14992500 | -1.16195200 | 1.02015500 |
| 8 | C | -0.96482400 | -0.46997400 | -0.06378900 |
| 9 | C | -0.83850300 | -1.80517000 | 2.23218400 |
| 10 | C | -1.87513600 | -0.91948300 | 2.94450700 |
| 11 | C | -1.43513100 | -3.19208400 | 1.91221600 |
| 12 | C | -1.25173200 | 1.03895600 | 0.22909300 |
| 13 | C | -0.09959200 | 1.93361900 | -0.18196100 |
| 14 | C | 1.96541000 | -2.83975800 | -1.75451000 |
| 15 | C | 3.61004000 | 1.37482200 | -0.02989500 |
| 16 | C | 4.09566500 | 1.57253600 | -1.25870700 |
| 17 | C | 3.28507000 | 2.56004500 | 0.86992000 |
| 18 | C | 1.94119400 | 3.26851100 | 0.55748400 |
| 19 | C | 0.72753500 | 2.40630400 | 0.75824100 |
| 20 | C | 0.01537000 | 2.21792500 | -1.66118400 |
| 21 | O | -2.23542100 | -1.15613200 | -0.20956000 |
| 22 | O | -1.97189300 | -1.33094200 | -2.45189700 |
| 23 | C | -2.62994200 | -1.51144200 | -1.45428100 |
| 24 | C | -3.97963500 | -2.17690600 | -1.41626000 |
| 25 | O | -2.41613300 | 1.42508000 | -0.55422400 |
| 26 | O | -3.59188500 | 2.00937300 | 1.28993400 |
| 27 | C | -3.50633400 | 1.89840600 | 0.08954200 |
| 28 | C | -4.57970900 | 2.28059200 | -0.89513500 |
| 29 | H | 4.72908600 | -2.83455700 | -0.99906800 |
| 30 | H | 5.45890200 | -0.46331400 | -0.08543300 |
| 31 | H | 4.92467900 | -1.41467900 | 1.28608700 |
| 32 | H | 3.18313700 | 0.07864000 | 1.59602700 |
| 33 | H | 1.64428800 | -0.11833400 | -0.83527200 |
| 34 | H | 1.68659400 | -1.77261200 | 1.75347500 |
| 35 | H | -0.45598400 | -0.54501100 | -1.02100600 |
| 36 | H | -0.03191400 | -1.97941200 | 2.95190400 |
| 37 | H | -2.24703700 | -1.43754900 | 3.83336400 |
| 38 | H | -1.43718700 | 0.02631300 | 3.27488600 |
| 39 | H | -2.73268300 | -0.69647400 | 2.30728100 |
| 40 | H | -1.75179800 | -3.68206100 | 2.83840600 |
| 41 | H | -2.30213300 | -3.11292000 | 1.25635500 |
| 42 | H | -0.69454300 | -3.83363200 | 1.42758300 |
| 43 | H | -1.49544200 | 1.17764200 | 1.27806300 |
| 44 | H | 1.14222900 | -3.30523700 | -1.20229200 |
| 45 | H | 1.50985700 | -2.26494600 | -2.56905800 |
| 46 | H | 2.57403200 | -3.63233400 | -2.19471300 |
| 47 | H | 4.26770500 | 2.57206500 | -1.64586400 |
| 48 | H | 4.33575600 | 0.74790800 | -1.92193300 |
| 49 | H | 4.07671000 | 3.30955700 | 0.77421800 |
| 50 | H | 3.28372700 | 2.23839900 | 1.91613000 |
| 51 | H | 1.86504600 | 4.13519200 | 1.22569300 |
| 52 | H | 1.98087400 | 3.66344600 | -0.45907500 |
| 53 | H | 0.52366300 | 2.13321600 | 1.79246000 |
| 54 | H | -0.74365100 | 2.94130800 | -1.97388000 |
| 55 | H | 0.99441200 | 2.61082400 | -1.92906700 |
| 56 | H | -0.15875500 | 1.31585700 | -2.25453000 |
| 57 | H | -3.90638300 | -3.12273200 | -0.87384500 |
| 58 | H | -4.69757900 | -1.54794400 | -0.88745300 |
| 59 | H | -4.32155800 | -2.36589300 | -2.43150100 |
| 60 | H | -4.23357900 | 3.12097000 | -1.50171400 |
| 61 | H | -4.78807400 | 1.45041100 | -1.57193300 |
| 62 | H | -5.48282600 | 2.56457200 | -0.35931800 |

B3LYP/6-311G(d, p) Energy =-1236.2218936a.u.; Population =0.05% .
